# Effect of P2Y12 Inhibitors on Organ Support–Free Survival in Critically Ill Patients Hospitalized for COVID-19: A Randomized Clinical Trial

**DOI:** 10.1001/jamanetworkopen.2023.14428

**Published:** 2023-05-25

**Authors:** Jeffrey S. Berger, Matthew D. Neal, Lucy Z. Kornblith, Michelle N. Gong, Harmony R. Reynolds, Mary Cushman, Andrew D. Althouse, Patrick R. Lawler, Bryan J. McVerry, Keri S. Kim, Lisa Baumann Kreuziger, Scott D. Solomon, Mikhail N. Kosiborod, Scott M. Berry, Grant V. Bochicchio, Marco Contoli, Michael E. Farkouh, Joshua D. Froess, Sheetal Gandotra, Yonatan Greenstein, Erinn M. Hade, Nicholas Hanna, Kristin Hudock, Robert C. Hyzy, Fátima Ibáñez Estéllez, Nicole Iovine, Ashish K. Khanna, Pooja Khatri, Bridget-Anne Kirwan, Matthew E. Kutcher, Eric Leifer, George Lim, Renato D. Lopes, Jose L. Lopez-Sendon, James F. Luther, Lilia Nigro Maia, John G. Quigley, Lana Wahid, Jennifer G. Wilson, Ryan Zarychanski, Andrei Kindzelski, Mark W. Geraci, Judith S. Hochman

**Affiliations:** 1 Center for the Prevention of Cardiovascular Disease, NYU Grossman School of Medicine, New York, New York; 2 Department of Surgery, University of Pittsburgh, Pittsburgh, Pennsylvania; 3 Department of Surgery, University of California, San Francisco, San Francisco; 4 Department of Laboratory Medicine, University of California, San Francisco, San Francisco; 5 Albert Einstein College of Medicine, Bronx, New York; 6 Division of Cardiology, Department of Medicine, NYU Grossman School of Medicine, New York, New York; 7 University of Vermont College of Medicine, Burlington; 8 Department of Epidemiology, School of Public Health, University of Pittsburgh, Pittsburgh, Pennsylvania; 9 Now with Medtronic, Minneapolis, Minnesota.; 10 Peter Munk Cardiac Centre, Toronto, Ontario, Canada; 11 Department of Medicine, University of Pittsburgh, Pittsburgh, Pennsylvania; 12 College of Pharmacy, University of Illinois at Chicago, Chicago; 13 Versity Blood Research Institute, Milwaukee, Wisconsin; 14 Cardiovascular Medicine Division, Brigham and Women’s Hospital, Harvard Medical School, Boston, Massachusetts; 15 Saint Luke's Mid America Heart Institute, University of Missouri, Kansas City; 16 Berry Consultants, LLC, Austin, Texas; 17 Washington University School of Medicine, St Louis, Missouri; 18 Department of Translational Medicine, Università di Ferrara, Ferrara, Italy; 19 Division of Pulmonary, Allergy, and Critical Care Medicine, Department of Medicine, University of Alabama, Birmingham; 20 Rutgers New Jersey Medical School, Newark; 21 Division of Biostatistics, Department of Population Health, NYU Grossman School of Medicine, New York, New York; 22 Ascension St John Clinical Research Institute, Tulsa, Oklahoma; 23 University of Cincinnati Medical Center, Cincinnati, Ohio; 24 Division of Pulmonary and Critical Care, Department of Medicine, University of Michigan, Ann Arbor; 25 Hospital Emergencias Enfermera Isabel Zendal, Madrid, Spain; 26 Division of Infectious Diseases and Global Medicine, Department of Medicine, University of Florida, Gainesville; 27 Perioperative Outcomes and Informatics Collaborative, Wake Forest University School of Medicine, Winston-Salem, North Carolina; 28 Outcomes Research Consortium, Cleveland, Ohio; 29 Socar Research SA, Nyon, Switzerland; 30 University of Mississippi Medical Center, Jackson; 31 National Heart Lung and Blood Institute, National Institutes of Health, Bethesda, Maryland; 32 Ronald Reagan UCLA Medical Center, Los Angeles, California; 33 Division of Cardiology and Duke Clinical Research Institute, Duke University Medical Center, Durham; 34 IdiPaz Research Institute, Hospital Universitario La Paz, Madrid, Spain; 35 Fundação Faculdade Regional De Medicina De São José Do Rio Preto, São José do Rio Preto, Brazil; 36 Division of Hematology and Oncology, Department of Medicine, University of Illinois at Chicago; 37 Division of Internal Medicine, Department of Medicine, Duke University Medical Center, Durham, North Carolina; 38 Stanford University Medical Center, Stanford, California; 39 Department of Medicine, University of Manitoba, Winnipeg, Manitoba, Canada

## Abstract

**Importance:**

Platelet activation is a potential therapeutic target in patients with COVID-19.

**Objective:**

To evaluate the effect of P2Y12 inhibition among critically ill patients hospitalized for COVID-19.

**Design, Setting, and Participants:**

This international, open-label, adaptive platform, 1:1 randomized clinical trial included critically ill (requiring intensive care–level support) patients hospitalized with COVID-19. Patients were enrolled between February 26, 2021, through June 22, 2022. Enrollment was discontinued on June 22, 2022, by the trial leadership in coordination with the study sponsor given a marked slowing of the enrollment rate of critically ill patients.

**Intervention:**

Participants were randomly assigned to receive a P2Y12 inhibitor or no P2Y12 inhibitor (usual care) for 14 days or until hospital discharge, whichever was sooner. Ticagrelor was the preferred P2Y12 inhibitor.

**Main Outcomes and Measures:**

The primary outcome was organ support–free days, evaluated on an ordinal scale that combined in-hospital death and, for participants who survived to hospital discharge, the number of days free of cardiovascular or respiratory organ support up to day 21 of the index hospitalization. The primary safety outcome was major bleeding, as defined by the International Society on Thrombosis and Hemostasis.

**Results:**

At the time of trial termination, 949 participants (median [IQR] age, 56 [46-65] years; 603 male [63.5%]) had been randomly assigned, 479 to the P2Y12 inhibitor group and 470 to usual care. In the P2Y12 inhibitor group, ticagrelor was used in 372 participants (78.8%) and clopidogrel in 100 participants (21.2%). The estimated adjusted odds ratio (AOR) for the effect of P2Y12 inhibitor on organ support–free days was 1.07 (95% credible interval, 0.85-1.33). The posterior probability of superiority (defined as an OR > 1.0) was 72.9%. Overall, 354 participants (74.5%) in the P2Y12 inhibitor group and 339 participants (72.4%) in the usual care group survived to hospital discharge (median AOR, 1.15; 95% credible interval, 0.84-1.55; posterior probability of superiority, 80.8%). Major bleeding occurred in 13 participants (2.7%) in the P2Y12 inhibitor group and 13 (2.8%) in the usual care group. The estimated mortality rate at 90 days for the P2Y12 inhibitor group was 25.5% and for the usual care group was 27.0% (adjusted hazard ratio, 0.96; 95% CI, 0.76-1.23; *P* = .77).

**Conclusions and Relevance:**

In this randomized clinical trial of critically ill participants hospitalized for COVID-19, treatment with a P2Y12 inhibitor did not improve the number of days alive and free of cardiovascular or respiratory organ support. The use of the P2Y12 inhibitor did not increase major bleeding compared with usual care. These data do not support routine use of a P2Y12 inhibitor in critically ill patients hospitalized for COVID-19.

**Trial Registration:**

ClinicalTrials.gov Identifier: NCT04505774

## Key Points

**Question:**

What is the effect of P2Y12 inhibition, a proposed therapeutic target and preventive strategy, on clinical outcomes in critically ill patients hospitalized for COVID-19?

**Findings:**

In this randomized clinical trial that included 949 participants, use of a P2Y12 inhibitor did not result in a greater number of days alive and free of cardiovascular or respiratory organ support up to day 21 of the index hospitalization.

**Meaning:**

These data do not support routine use of a P2Y12 inhibitor in critically ill patients hospitalized for COVID-19.

## Teaser

This randomized clinical trial of patients hospitalized for COVID-19 examines whether P2Y12 inhibition increased days alive and free of organ support.

## Introduction

The COVID-19 pandemic has resulted in significant morbidity and mortality, with critically ill patients being at highest risk.^1,2,3^ Critically ill patients with COVID-19 are at particularly high risk for vascular dysfunction and microvascular and macrovascular thrombotic events.^2^ In addition to mediating thrombosis, platelets influence inflammation and immune function.^4,5^

Autopsy and clinical data highlight the potential role of platelets in the pathogenesis of COVID-19.^6,7^ Platelets isolated from patients with COVID-19 are hyperreactive, have a prothrombotic genetic expression profile, and induce activation of myeloid and endothelial cells.^8,9,10^ Thus, platelets are a therapeutic target of interest because of the potential to modulate thrombosis, inflammation, and vasculopathy. In the antiplatelet domain of the REMAP-CAP (Randomized Embedded Multifactorial Adaptive Platform for Community-acquired Pneumonia) platform, antiplatelet therapy with a P2Y12 inhibitor or aspirin did not improve the need for organ support, although there was a 97% probability that antiplatelet therapy (including both aspirin and P2Y12 inhibitors) improved survival to hospital discharge in critically ill patients with COVID-19.^11^ The ACTIV-4a (A Multicenter, Adaptive, Randomized Controlled Platform Trial of the Safety and Efficacy of Antithrombotic and Additional Strategies in Hospitalized Adults With COVID-19) platform domain evaluating the effects of P2Y12 inhibition in patients hospitalized with COVID-19 identified no clinical benefit in noncritically ill patients.^12^ Because the benefit of other therapies in COVID-19 may vary according to disease severity,^13,14,15^ we continued to study P2Y12 inhibitors in critically ill patients. Accordingly, we conducted an international, adaptive, controlled platform randomized clinical trial to evaluate whether P2Y12 inhibition increased days alive and free of organ support, the composite of survival to hospital discharge and days free of cardiovascular or respiratory organ support through day 21, in critically ill patients hospitalized for COVID-19.

## Methods

### Trial Design and Oversight

The ACTIV-4a trial, supported by the National Institutes of Health, was designed to evaluate the effects of antithrombotic therapies in patients hospitalized with COVID-19. The trial, coordinated at NYU Grossman School of Medicine and the University of Pittsburgh, enrolls participants across a collaborative consortium of clinical trial networks. The trial is an adaptive platform trial that uses a master protocol to longitudinally study multiple investigational treatments.^16,17^ The study received Central Institutional Review Board approval and was registered at ClinicalTrials.gov (NCT04505774). After completion of the initial testing of prophylactic-dose and therapeutic-dose heparins as part of the multiplatform randomized clinical trials,^13,14^ the ACTIV-4a platform has continued to evaluate other investigational treatments. Concurrent randomization was permitted into the P2Y12 and sodium-glucose cotransporter 2 (SGLT2) inhibitor domain but not the P2Y12 and crizanlizumab domain. We report the results of P2Y12 inhibitor vs no P2Y12 inhibitor (usual care) in critically ill patients.

ACTIV-4a Investigators and collaborators are listed in eAppendix 1 in Supplement 2, and additional supplemental methods are detailed in eAppendix 2 in Supplement 2. Written informed consent was obtained from participants or their legally authorized representatives if the participants were unable to provide consent. The trial was conducted in accordance with the principles of the Good Clinical Practice guidelines of the International Conference on Harmonization. The protocol and statistical analysis plan are available in Supplement 1. This study followed the Consolidated Standards of Reporting Trials (CONSORT) reporting guideline.

### Enrollment

**Figure 1.**
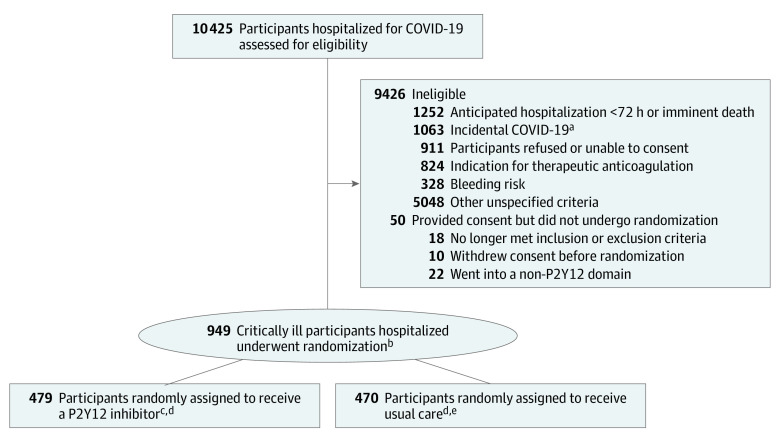
Eligibility, Randomization, and Follow-up in the ACTIV-4a Trial of P2Y12 Inhibitors in Critically Ill Patients With COVID-19 ^a^COVID-19 confirmed by positive test result, but reason for hospitalization was for an unrelated condition. ^b^Randomization was stratified by site and by severity of illness. ^c^Four hundred seventy-five included in the organ support–free days end point. ^d^Six patients withdrew on day 1 or 2 after randomization and are excluded from the primary outcome analysis in accordance with the statistical analysis plan because they cannot be assigned a value for the 21-day analysis. However, these 6 patients are included in the safety analyses, so if these patients had any adverse events, they would be included. ^e^Four hundred sixty-eight included in the organ support–free days end point.

The first participant was randomly assigned to a study group on February 26, 2021. Enrollment was discontinued in the critically ill group on June 22, 2022, by the trial leadership in coordination with the study sponsor given a marked slowing of the enrollment rate of critically ill patients. At that time, 479 participants were randomly assigned to receive a P2Y12 inhibitor, and 470 participants were randomly assigned to usual care (Figure 1).

### Participants

Patients were eligible for the trial if they had laboratory-confirmed SARS-CoV-2 infection and were hospitalized for COVID-19. Confirmation of SARS-CoV-2 positivity was determined by site-specific laboratory standards. The investigators hypothesized that the benefits and risks of a P2Y12 inhibitor varied according to disease severity. As such, the ACTIV-4a design stratified participants as critically ill (requiring intensive care–level support) and noncritically ill (hospitalized but not receiving intensive care–level support) at enrollment. Intensive care–level support was defined by use of respiratory or cardiovascular organ support (oxygen via high-flow nasal cannula ≥20 L/min, noninvasive or invasive mechanical ventilation, vasopressors, inotropes, or extracorporeal membrane oxygenation support).^13^ This report describes the results of the analyses for critically ill participants hospitalized for COVID-19. Results for noncritically ill participants were previously published.^12^ Patients were ineligible for enrollment into this domain of the platform if more than 72 hours had elapsed from the later date of hospital admission for COVID-19 or SARS-CoV-2 confirmation, if discharge was expected within 72 hours, or if they had a contraindication to a P2Y12 inhibitor or a clinical requirement for dual antiplatelet therapy (see the study protocol in Supplement 1 for detailed eligibility criteria). This trial collected self-reported race and ethnicity data from either the participants or their surrogates via fixed categories appropriate to their region per National Institutes of Health guidelines.

### Randomization

Participants were randomly assigned to receive a P2Y12 inhibitor or no P2Y12 inhibitor (usual care) in open-label fashion by a 1:1 ratio in permuted block sizes of 2 or 4 and stratified by site and severity of illness using a web-based system (eSOCDAT, Socar Research). Because of its fast onset with consistent and potent platelet inhibitory effects,^18^ ticagrelor was the preferred P2Y12 inhibitor; however, clopidogrel and prasugrel were permitted. When ticagrelor was used, 60 mg twice daily was the recommended dose without a loading dose. When clopidogrel was used, a loading dose of 300 mg was recommended. Additional details of dosing are available in the study protocol (Supplement 1). Duration of P2Y12 inhibitor treatment was 14 days or until hospital discharge, whichever came first. All critically ill participants were recommended to receive prophylactic-dose heparin based on reported findings in COVID-19 as part of their standard care.^14^ Adherence to study medication (P2Y12 inhibitor) and heparin dose was measured at the end of study day 1.

### Outcome Measures

The composite primary outcome was organ support–free days, an ordinal scale composed of survival to hospital discharge and, in survivors, the number of days free of intensive care–level respiratory or cardiovascular organ support during the index hospitalization, through day 21. Any death during the index hospitalization through 90 days was assigned the worst outcome (−1). This end point reflects both use of intensive care–level therapies and hospital survival, with higher values indicating better outcomes.

A key secondary outcome was major thrombotic events or death (a composite of myocardial infarction, pulmonary embolism, ischemic stroke, systemic arterial embolism, and in-hospital death). Other secondary efficacy outcomes included survival to hospital discharge, survival without receipt of respiratory or cardiovascular organ support, and all thrombotic events (major thrombotic events plus deep venous thrombosis) or death. The primary safety outcome was major bleeding, as defined by the International Society on Thrombosis and Hemostasis. A secondary safety outcome was the composite of major bleeding or death. All reported bleeding and thrombotic events were adjudicated in an anonymized fashion by the clinical end points committee using consensus definitions (Supplement 1).

### Statistical Analysis

Trial results were analyzed in a bayesian framework, as described in the statistical analysis plan and study protocol (Supplement 1). The design was adaptive in sample size with a range of 200 to 2000 patients. Adaptive analyses were planned to occur no more often than monthly, with a minimum of 200 patients randomly assigned between analyses. Randomization was set to continue until a statistical threshold for superiority (defined as >99% posterior probability of proportional odds ratio [OR] > 1) or futility (>95% posterior probability of proportional OR < 1.2) was met. The overall adaptive sample size yields more than 80% power for an OR of 1.25 in the organ-support-free-days outcome. There is approximately 90% power for an OR of 1.5 with 400 patients per treatment group.

The primary analysis used a bayesian cumulative logistic regression model (presented in the statistical analysis plan in Supplement 1) that calculated the posterior probability distribution for the proportional OR for the P2Y12 inhibitor compared with no P2Y12 inhibitor (usual care) on organ support–free days. An OR greater than 1 indicates a higher number of organ support–free days with the active treatment. The model estimates distinct treatment effects in critically ill and noncritically ill participants but permits data borrowing between illness severity groups on the basis of similarity in effect estimates. The primary model adjusts for age, sex, site, mechanical ventilation at enrollment, history of cardiovascular disease (composite of hypertension, heart failure, diabetes, coronary artery disease, peripheral artery disease, and cerebrovascular disease), SGLT2 inhibitor assignment, and period (in 2-week intervals). The primary model incorporated a weakly informative Dirichlet prior distribution for the outcome of organ support–free days. The model was fit using a Markov Chain Monte Carlo algorithm with 100 000 samples from the joint posterior distribution, allowing calculation of the posterior distributions for the proportional ORs, including medians and 95% credible intervals (CrIs), and the posterior probabilities of superiority and futility for P2Y12 inhibitor compared with usual care.

A post hoc sensitivity analysis was performed without the use of dynamic borrowing and after excluding patients who received therapeutic-dose heparin because this was deemed to be an inferior concurrent treatment in critically ill patients with COVID-19.^13^ Similar bayesian analyses were performed for the 3-level (death, alive with mechanical ventilation, and alive with organ support without mechanical ventilation) and 4-level (death, alive with invasive mechanical ventilation, alive with noninvasive mechanical ventilation, and alive with organ support without mechanical ventilation) derivatives of the primary outcome with a cumulative logistic model and a flat Dirichlet prior.

Consistent with our prior approach,^12^ we also used frequentist logistic and cumulative logistic models for the outcome of organ support–free days and its components. As noted in eTable 1 in Supplement 2, the results obtained from the bayesian and frequentist models were consistent. Thus, we used the frequentist approach for secondary outcomes and the prespecified subgroup analyses.

Logistic and cumulative logistic models were used for binary and ordinal outcomes in the secondary and subgroup analyses. The ORs and 95% CIs were estimated through a random-effects logistic regression model that accounted for the same covariates in the primary analysis model. For time remaining alive through 90 days, hazard ratios and 95% CIs were estimated through a frailty proportional hazards model that accounted for the same covariates in the primary analysis model.

The proportional hazards assumptions were tested using 2 approaches: (1) comparison of survival curves and log[-log] curves by treatment and (2) including parameters for covariate × log(time) interaction. Both approaches suggested proportional hazards. For the subgroup analyses, we included a treatment × group interaction in the model and set up contrasts to compare use of a P2Y12 inhibitor with usual care within each subgroup.

All frequentist analyses were run in SAS software, version 9.4 (SAS Institute Inc) with 2-sided tests at the significance level of *P* < .05. Because of the potential for type I error due to multiple comparisons, the findings for the secondary and subgroup analyses should be interpreted as exploratory.

## Results

### Participants

The median (IQR) age of the 949 randomly assigned participants was 56 (46-65) years; 346 (36.5%) were female and 603 (63.5%) were male; 13 (1.4%) were American Indian or Alaska Native, 32 (3.4%) were Asian, 190 (20.0%) were Black, 12 (1.3%) were Native Hawaiian or Pacific Islander, 561 (59.1%) were White, 8 (0.8%) reported being of multiple races, and 53 (5.6%) reported other race or ethnicity. The baseline characteristics were balanced between the groups (Table 1). At randomization, 733 (77.2%) were receiving high-flow nasal cannula, 108 (11.5%) were receiving noninvasive ventilation, 102 (10.8%) were receiving mechanical ventilation, and 64 (6.7%) were receiving vasopressors or inotropes. Concomitant baseline therapies included corticosteroids (792 [83.5%]), remdesivir (574 [60.5%]), and interleukin 6 receptor antagonists (147 [15.5%]). Consistent with the study recommendations, most participants received prophylactic-dose heparin (482 [92.6%] in the P2Y12 inhibitor group and 410 [91.9%] in the usual care group) following randomization.

**Table 1.** Baseline Participant Characteristics^a^

Characteristic	P2Y12 inhibitor (n = 479)	Usual care (n = 470)
Age, mean (SD), y	55.1 (13.5)	54.7 (14.2)
Sex		
Female	171/479 (35.7)	175/470 (37.2)
Male	308/479 (64.3)	295/470 (62.8)
Race and ethnicity^b^		
American Indian or Alaska Native	3/442 (0.7)	10/427 (2.3)
Asian	13/442 (2.9)	19/427 (4.4)
Black	100/442 (22.6)	90/427 (21.1)
Hispanic	138/465 (29.7)	132/453 (29.1)
Native Hawaiian or Pacific Islander	6/442 (1.4)	6/427 (1.4)
White	288/442 (65.2)	273/427 (63.9)
Multiple races	2/442 (0.5)	6/427 (1.4)
Other^c^	30/442 (6.8)	23/427 (5.4)
Hospital location		
Brazil	21/479 (4.4)	20/470 (4.3)
Italy	18/479 (3.8)	17/470 (3.6)
Mexico	2/479 (0.4)	4/470 (0.9)
Spain	45/479 (9.4)	45/470 (9.6)
US	393/479 (82.0)	384/470 (81.7)
BMI, median (IQR)	32.6 (27.9-38.6) (n = 460)	31.8 (27.7-37.9) (n = 458)
BMI>30	286/460 (62.2)	269/458 (58.7)
Cardiovascular disease	241/479 (50.3)	218/470 (46.4)
Hypertension	236/479 (49.3)	209/470 (44.5)
Heart failure	20/479 (4.2)	24/470 (5.1)
Coronary artery disease	22/479 (4.6)	34/470 (7.2)
Peripheral arterial disease	3/479 (0.6)	2/470 (0.4)
Cerebrovascular disease	11/479 (2.3)	9/470 (1.9)
Other diseases and chronic conditions^d^	162/479 (33.8)	149/470 (31.7)
Diabetes	141/479 (29.4)	126/470 (26.8)
Chronic kidney disease	35/479 (7.3)	33/470 (7.0)
Liver disease	15/479 (3.1)	14/470 (3.0)
Respiratory disease^d^^,^^e^	61/479 (12.7)	79/470 (16.8)
Asthma	36/479 (7.5)	51/470 (10.9)
COPD	29/479 (6.1)	34/470 (7.2)
Baseline treatment at enrollment		
Corticosteroids	399/478 (83.5)	393/470 (83.6)
Anticoagulant		
None	156/470 (33.2)	159/458 (34.7)
Prophylactic	284/470 (60.4)	260/458 (56.8)
Therapeutic	30/470 (6.4)	39/458 (8.5)
Remdesivir	289/478 (60.5)	285/458 (60.6)
Aspirin	79/477 (16.6)	91/458 (19.4)
Baricitinib	86/478 (18.0)	88/458 (18.7)
IL-6 inhibitors	75/478 (15.7)	72/458 (15.3)
Organ support	479/479 (100)	470/470 (100)
Respiratory support		
High-flow nasal cannula (>20 L/min)	369/478 (77.2)	364/469 (77.6)
Noninvasive ventilation	56/478 (11.7)	52/469 (11.1)
Invasive mechanical ventilation	51/478 (10.7)	51/469 (10.9)
Vasopressors or inotropes	29/478 (6.1)	35/469 (7.4)
Laboratory values, median (IQR)^f^		
D-dimer, μg/mL (FEU)	1.2 (0.8-2.2) (n = 400)	1.0 (0.7-1.8) (n = 383)
D-dimer ≥2 × the ULN	222/400 (55.5)	183/383 (47.8)
C-reactive protein, mg/dL	12.5 (7.3-19.1) (n = 332)	10.7 (5.6-17.9) (n = 328)
Creatinine, mg/dL	0.90 (0.71-1.17); (n = 476)	0.87 (0.70-1.13); (n = 468)
Hemoglobin, g/dL	13.4 (12.3-14.5); (n = 475)	13.3 (12.3-14.6); (n = 468)
Lymphocyte count, /μL	720 (500-1020) (n = 399)	710 (500-1100) (n = 410)
Platelet count, ×10^3^/μL	236 (176-297) (n = 476)	232 (177-300) (n = 468)

Abbreviations: BMI, body mass index (calculated as weight in kilograms divided by height in meters squared); COPD, chronic obstructive pulmonary disease; FEU, fibrinogen equivalent units; IL-6, interleukin 6; ULN, upper limit of normal.

SI conversion factors: To convert D-dimer to nanomoles per liter, multiply by 5.476; C-reactive protein to milligrams per liter, multiply by 10; creatinine to micromoles per liter, multiply by 88.4; hemoglobin to grams per liter, multiply by 10; lymphocytes to ×10^9^/L, multiply by 0.001; and platelets to ×10^9^/L, multiply by 1.

^a^ Data are presented as number (percentage) or number/total number (percentage) of patients unless otherwise indicated.

^b^ This trial collected self-reported race and ethnicity data from either the participants or their surrogates via fixed categories appropriate to their region.

^c^ Not further specified.

^d^ Data were systematically collected for each patient using predefined questions in the electronic case report form. If this information was not available and/or not reported in the hospital records, the site personnel ticked either “not reported” or “information not available” (as appropriate).

^e^ Includes home oxygen therapy, asthma, COPD, bronchiectasis, interstitial lung disease, lung cancer, pulmonary hypertension, and tuberculosis.

^f^ Each site provided the local reference ranges for the assays: C-reactive protein, creatinine, hemoglobin, lymphocyte count, and platelet count. The reference ranges differed per site.

Initial adherence to protocol-assigned P2Y12 inhibitor was high, with 472 of 479 participants (98.5%) receiving at least 1 dose of a P2Y12 inhibitor, either ticagrelor (372 [78.8%]) or clopidogrel (100 [21.2%]) (eTable 2 in Supplement 2). Of participants receiving clopidogrel, 82 (82.0%) received a loading dose in accordance with study protocol recommendations. The median (IQR) duration of study drug treatment was 9 (6-14) days. The median (IQR) percentage of index hospital days during which participants received study drug after randomization was 100% (100%-100%). In the usual care group, 2 participants received a P2Y12 inhibitor, 1 on the day of discharge and 1 on day 13.

### Primary Outcome

**Figure 2.**
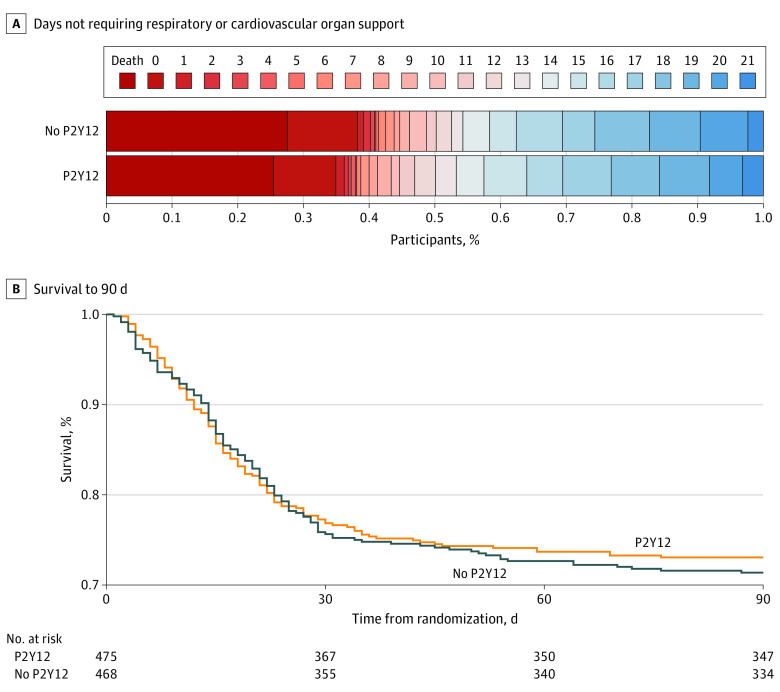
Effect of Randomization to a P2Y12 Inhibitor on the Number of Days Not Requiring Respiratory or Cardiovascular Organ Support and Survival Through 90 Days in Critically Ill Patients With COVID-19 (A) Data are shown as the number of days not requiring respiratory or cardiovascular organ support (eg, oxygen via high-flow nasal cannula ≥20 L/min, noninvasive or invasive mechanical ventilation, vasopressors, inotropes, or extracorporeal membrane oxygenation) as horizontally stacked proportions of patients in the 2 treatment groups, with the following possible outcomes: in-hospital death with or without organ support (dark red, the worst possible outcome, corresponding to a score of −1 on the ordinal scale), survival with organ support (red to blue gradient shading based on the number of days alive without organ support, corresponding to a score of 0-21 on the ordinal scale), and survival until hospital discharge without organ support (dark blue, the best possible outcome, corresponding to a score of 21 on the ordinal scale). (B) Kaplan-Meier curve showing survival through 90 days in critically ill patients. The 90-day survival in patients assigned to a P2Y12 inhibitor vs usual care was not significantly different in both a bayesian piecewise exponential model (adjusted hazard ratio, 0.99; 95% CI, 0.77-1.27; posterior probability of superiority, 53%) and a frequentist Cox proportional hazards regression model (adjusted hazard ratio, 0.96; 95% CI, 0.76-1.23; *P* = .77).

The median (IQR) number of organ support–free days was 12 (−1 to 17) in the P2Y12 inhibitor group and 11 (−1 to 18) in the usual care group. The median adjusted OR (AOR) for the effect of P2Y12 inhibitor on organ support–free days was 1.07 (95% CrI, 0.85-1.33; posterior probability of superiority, 72.9%) (Figure 2A and Table 2).

**Table 2.** Primary Outcome of 21-Day Organ Support–Free Days and Survival to Hospital Discharge

Outcome	P2Y12 inhibitor (n = 475)	Usual care (n = 468)	Absolute difference (95% CI)	Median adjusted odds ratio (95% CrI)	Probability, %^a^		
					Futility (OR < 1.2)	Superiority (OR > 1)	Inferiority (OR < 1)
Organ support–free days (primary end point), median (IQR)^a^^,^^b^^,^^c^	12.0 (−1.0 to 17.0)	11.0 (−1.0 to 18.0)	NA	1.07 (0.85 to 1.33)^d^	83.4	72.9	27.1
Survival to hospital discharge (up to 90 d), No. (%)^e^	354 (74.5)	339 (72.4)	2.1 (−3.6 to 7.7)	1.15 (0.84 to 1.55)	63.3	80.8	19.2

Abbreviations: CrI, credible interval; NA, not applicable; OR, odds ratio.

^a^ Composite ordinal scale consisting of survival to hospital discharge and days free of organ support to day 21. An OR greater than 1 indicates a benefit from treatment. Probabilities of superiority (proportional OR >1), inferiority (proportional OR <1), and futility (proportional OR <1.2) are computed from the posterior distribution of the proportional OR for P2Y12 inhibitor compared with no P2Y12 inhibitor on the outcome.

^b^ The model incorporated dynamic borrowing from 562 noncritically ill participants in the original ACTIV-4a study. The mean treatment effect in each group was assumed to follow a hierarchical normal distribution with the same mean, which created a dynamic amount of borrowing, depending on the similarity across groups.

^c^ Effect of P2Y12 inhibitor on organ support–free days without dynamic borrowing from the critically ill cohort yielded an adjusted OR of 1.09 (95% CrI, 0.86-1.38), with a posterior probability of superiority of 76.8%.

^d^ Adjusted for age, sex, enrollment epoch, cardiovascular disease (composite of hypertension, heart failure, coronary artery disease, peripheral artery disease, and cerebrovascular disease), sodium-glucose cotransporter 2 assignment, and baseline mechanical ventilation; study country and site are treated as nested random effects.

^e^ Effect of P2Y12 inhibitor on survival to hospital discharge without dynamic borrowing from the critically ill cohort yielded an adjusted OR of 1.16 (95% CrI, 0.84-1.62), with a posterior probability of superiority of 81.1%.

### Sensitivity and Subgroup Analyses

Results of the frequentist cumulative logistic model for the primary outcome were similar (eTable 1 in Supplement 2). After excluding 84 participants who received therapeutic-dose heparin, the estimated AOR for the effect of a P2Y12 inhibitor on organ support–free days was 1.00 (95% CrI, 0.78-1.31) (eTable 3 in Supplement 2). In subgroup analyses, the treatment effect did not vary meaningfully by sex, race, mechanical ventilation at enrollment, or site preference for ticagrelor or clopidogrel (eTable 4 in Supplement 2). We observed a significant interaction between age and treatment effect of P2Y12 inhibitors (*P* = .02), with a potential benefit of P2Y12 inhibitors on the primary end point of organ support–free days in adults younger than 65 years (AOR, 1.23; 95% CrI, 0.95-1.60) and fewer organ support–free days among adults 65 years or older (AOR, 0.66; 95% CrI, 0.41-1.04).

### Secondary Outcomes

Overall, 354 participants (74.5%) in the P2Y12 inhibitor group and 339 participants (72.4%) in the usual care group survived to hospital discharge (median aOR, 1.15; 95% CrI, 0.84-1.55; posterior probability of superiority, 80.8%). Data on thrombotic, bleeding, and other key secondary outcomes are provided in Table 3. The composite of major thrombotic event or in-hospital death did not differ between the 135 participants (28.2%) in the P2Y12 inhibitor group and the 121 participants (25.7%) in the usual care group (AOR, 1.10; 95% CI, 0.81-1.50) (Table 3; eTable 5 in Supplement 2). Major bleeding occurred in 13 participants (2.7%) in the P2Y12 inhibitor group and 13 participants (2.8%) in the usual care group (AOR, 0.99; 95% CI, 0.44-2.22) (Table 3; eTable 6 in Supplement 2). Fatal bleeding or symptomatic bleeding in a critical area or organ occurred in 5 participants (1.0%) in the P2Y12 inhibitor group and 2 participants (0.4%) in the usual care group (AOR, 2.33; 95% CI, 0.45-12.2). The effect of P2Y12 inhibitor on survival for 90 days is shown in Figure 2B. The estimated mortality rates at 90 days were 25.5% for the P2Y12 inhibitor group and 27.0% for the usual care group (adjusted hazard ratio, 0.96; 95% CI, 0.76-1.23; *P* = .77).

**Table 3.** Key and Other Secondary Outcomes at 28 Days

Outcome^a^	P2Y12 inhibitor (n = 479)	Usual care (n = 470)	Absolute difference (95% CI)	Adjusted odds ratio (95% CI)^b^^,^^c^
Major thrombotic event or in-hospital death	135 (28.2)	121 (25.7)	2.4 (−3.2 to 8.1)	1.10 (0.81 to 1.50)
Major thrombotic event^d^	45 (9.4)	30 (6.4)	3.0 (−0.4 to 6.4)	1.51 (0.92 to 2.46)
In-hospital death	104 (21.7)	105 (22.3)	−0.6 (−5.9 to 4.6)	0.91 (0.65 to 1.27)
Any thrombotic event or in-hospital death	152 (31.7)	142 (30.2)	1.5 (−4.4 to 7.4)	1.04 (0.78 to 1.40)
Any thrombotic event^e^	73 (15.2)	64 (13.6)	1.6 (−2.8 to 6.1)	1.14 (0.78 to 1.66)
Major bleeding event or in-hospital death	115 (24.0)	112 (23.8)	0.2 (−5.2 to 5.6)	0.96 (0.69 to 1.33)
Major bleeding event^f^	13 (2.7)	13 (2.8)	−0.1 (−2.1 to 2.0)	0.99 (0.44 to 2.22)
Fatal bleeding event	3 (0.6)	2 (0.4)	0.2 (−0.7 to 1.1)	Too few events

^a^ Data expressed as number (percentage).

^b^ Odds ratio less than 1 corresponds to treatment benefit (eg, treatment is associated with fewer events).

^c^ Adjusted for age, sex, enrollment epoch, cardiovascular disease (composite of hypertension, heart failure, coronary artery disease, peripheral artery disease, and cerebrovascular disease), sodium-glucose cotransporter 2 assignment, and baseline mechanical ventilation; study country and site are treated as nested random effects.

^d^ Major thrombotic events include pulmonary embolism, myocardial infarction, ischemic stroke, and other arterial or venous thromboembolic events.

^e^ Any thrombotic events include major thrombotic events and deep vein thrombosis.

^f^ Major bleeding events are defined accordingly by the International Society on Thrombosis and Hemostasis: fatal bleeding and/or symptomatic bleeding in a critical area or organ, such as intracranial, intraspinal, intraocular, retroperitoneal, intra-articular or pericardial, or intramuscular with compartment syndrome, and/or bleeding causing a decrease in hemoglobin level of 2 g/dL or more (to convert to grams per liter, multiply by 10) or leading to transfusion of 2 or more units of whole blood or red cells.

## Discussion

In this open-label, international, multicenter, controlled platform randomized clinical trial of critically ill patients hospitalized for COVID-19, P2Y12 inhibitors did not significantly improve the composite of survival to hospital discharge or the number of days free of cardiovascular or respiratory organ support. The posterior probability of superiority for the primary end point was 73%, which did not meet the predefined criteria for superiority (>99% probability of OR >1). Survival to hospital discharge and through 90 days did not significantly differ with treatment assignment. Major bleeding complications were infrequent and balanced between the treatment groups.

Platelets are hyperreactive and proinflammatory in COVID-19.^7,8,9,10,19^ We hypothesized that platelet inhibition would reduce microthrombotic and macrothrombotic complications of COVID-19 because of their known regulation of thrombosis, inflammation, and vascular homeostasis and therefore could improve survival free of organ support. The findings of previous studies of antiplatelet therapy in hospitalized patients with COVID-19 have been inconsistent. In the RECOVERY (Randomized Evaluation of COVID-19 Therapy) trial, randomization to aspirin was not associated with a reduction in 28-day mortality or the risk of progressing to invasive mechanical ventilation or death.^20^ Although a higher proportion of participants randomized to aspirin were discharged from the hospital alive within 28 days (75% vs 74%; rate ratio, 1.06; 95% CI, 1.02-1.10; *P* = .006),^20^ the clinical relevance of this difference is debatable. In the REMAP-CAP trial, treatment with an antiplatelet agent (aspirin or clopidogrel) had a low likelihood of improving the number of organ support–free days within 21 days (95.7% posterior probability of futility).^11^ In contrast, similar to the RECOVERY trial, more patients survived to hospital discharge in the antiplatelet group than the control group (71.5% vs 67.9%; adjusted absolute difference, 5%; 95% CrI, −0.2% to 9.5%; posterior probability of efficacy of 97.0% for antiplatelet therapy compared with control) and at day 90 (median adjusted hazard ratio, 1.22; 95% CrI, 1.06-1.40; 99.7% posterior probability of improved survival of the pooled antiplatelet group compared with control).^11^ Our study found no significant benefit for the outcome of survival to hospital discharge. Most recently, data from COVID-PACT (Prevention of Arteriovenous Thrombotic Events in Critically Ill COVID-19 Patients Trial) found no clinical benefit from clopidogrel therapy in intensive care unit–level patients with COVID-19.^21^ In summary, findings across these trials suggest that platelet antagonism is unlikely to benefit patients with COVID-19.

The lack of demonstrable benefit of antiplatelet therapies in patients hospitalized for COVID-19 in previous trials could be partially explained by the testing of platelet inhibitors of multiple pathways (aspirin and P2Y12 inhibitors). We hypothesized that a focus on antagonism of the P2Y12 receptor pathway alone would be beneficial in patients with COVID-19 by modulating not only platelet aggregation and thrombosis but also platelet-mediated inflammation.^22,23^ As such, the ACTIV-4a platform focused antiplatelet investigations on potent P2Y12 inhibitors and specifically ticagrelor. Contrary to our hypothesis, we found no benefit in critically ill and noncritically ill patients.^12^ Specifically, P2Y12 inhibition did not increase the probability of survival to hospital discharge or the number of days free of cardiovascular or respiratory organ support in critically ill patients by the prespecified criteria. The reported rates of major bleeding were both low and similar between groups, and the rates of fatal or symptomatic bleeding in a critical area or organ were numerically higher in the P2Y12 inhibitor group. Importantly, the trial was stopped early because of a decrease in the number of hospitalized critically ill patients with recent Omicron variants and therefore a marked slowing of the enrollment rate over time. Although no definitive conclusion was demonstrated for the a priori defined end points of superiority (OR >1) or futility (OR <1.2), our results have clinical utility in that they do not support the routine use of a P2Y12 inhibitor in critically ill patients hospitalized for COVID-19.

An interesting finding in our subgroup analyses was that that participants 65 years or older appeared to have a worse outcome with P2Y12 inhibitors. In contrast, a potential benefit was observed in critically ill participants younger than 65 years. Increasing age is a major risk factor for bleeding^24^ and whether this excess bleeding risk observed in older individuals attenuates any benefit requires additional study. Moreover, the differential effect of P2Y12 inhibitors in patients with COVID-19 requires further validation.

### Strengths and Limitations

This study has several strengths. Despite the challenges of enrolling critically ill patients hospitalized for COVID-19 in clinical trials, participating sites recruited nearly 1000 high-risk patients and achieved high levels of adherence and follow-up, thereby ensuring that our hypotheses were rigorously tested. We tested therapies in the context of usual care, including the use of corticosteroids and remdesivir and over the course of several waves of the pandemic during which patients were likely exposed to different SARS-CoV-2 viral variants.

This study also has several limitations. First, the trial was open label, so ascertainment, performance, and reporting biases and the differential use of other therapies was possible. However, the primary outcome of organ support–free days and death was chosen to reduce bias given that these are ascertained directly from the medical record. We also found no evidence of differential use of heparin dosing according to treatment allocation (prophylactic-dose heparin was used in >90% of trial participants in both groups). Second, the assessment of P2Y12 inhibitors in critically ill patients occurred during approximately 16 months during which different SARS-CoV-2 viral variants emerged, potentially conferring different susceptibility to interventions. Although variants differed over time and therapies for COVID-19 evolved during the trial, we found no evidence of time-dependent effects of treatments evaluated in the trial. Third, many patients in the P2Y12 inhibitor group received clopidogrel. It is possible that the effect of P2Y12 inhibitors on critically ill patients with COVID-19 varies according to the type of P2Y12 inhibitor used. However, there was no evidence of a meaningful difference in the treatment effect according to hospital site proclivity for use of clopidogrel or ticagrelor. We tested treatment during 14 days or at hospital discharge, whichever came first. We therefore cannot assess the potential effect of longer durations of therapy on 90-day outcomes. Fourth, the ACTIV-4a trial was stopped early because of a marked decrease in the rate of enrollment of critically ill patients over time, and as such no definitive conclusion was demonstrated for the a priori defined end points. Despite these limitations, these trial results contribute meaningful findings to the body of literature indicating that antiplatelet therapy in patients hospitalized for COVID-19 does not have a compelling benefit, regardless of disease severity.

## Conclusions

In this randomized clinical trial among critically ill patients hospitalized for COVID-19, an initial strategy of P2Y12 inhibition did not result in a higher probability of survival to hospital discharge or a greater number of days free of cardiovascular or respiratory organ support. These results do not support the routine use of a P2Y12 inhibitor in critically ill patients hospitalized for COVID-19.

## Notes

Supplement 1.
Trial Protocol and Statistical Analysis Plan

Supplement 2. **eTable 1.** Comparison Between Bayesian and Frequentist Analyses **eTable 2.** Participants’ P2Y12 Inhibitor Type and Dosage Used **eTable 3.** Sensitivity Analyses of the Primary Outcome **eTable 4.** Subgroup Analysis on the Primary Endpoint (Organ Support Free Days to Day 21) **eTable 5.** Thrombotic Events Confirmed by Adjudication by Treatment Group **eTable 6.** Component Criteria on Which Major Bleeding Events Were Confirmed as Meeting the Definition of a Major Bleed Proposed by the International Society on Thrombosis and Hemostasis by Treatment Arm **eAppendix 1.** Investigators and Collaborators **eAppendix 2.** Supplemental Methods
eReferences

Supplement 3.
Nonauthor Collaborators

Supplement 4.
Data Sharing Statement
