# Supplementary material for: Effect of P2Y12 Inhibitors on Organ Support–Free Survival in Critically Ill Patients Hospitalized for COVID-19: A Randomized Clinical Trial
Source: JAMA Netw Open. 2023 May 25;6(5):e2314428. doi: 10.1001/jamanetworkopen.2023.14428 (PMC10214036; doi:10.1001/jamanetworkopen.2023.14428)
Supplement: Supplement 1. — Trial Protocol and Statistical Analysis Plan [file jamanetwopen-e2314428-s001.pdf]

A Multicenter, Adaptive, Randomized Controlled Platform Trial of the Safety and Efficacy of Antithrombotic Strategies in Hospitalized Adults with COVID-19

Short Title: ACTIV-4 ACUTE (AC-INPT)

ClinicalTrials.gov Number: NCT04505774

Supported by:
National Heart Lung and Blood Institutes (NHLBI), National Institute of Neurological Disorders (NINDS), ACTIV (ACTIV IV), National Institutes of Health (NIH), Biomedical Advanced Research and Development Authority (BARDA), Operation Warp Speed (OWS), U.S. Department of Health & Human Services (HHS)

Version Number: 1.1

01 FEB 2021

Protocol Revision History:

Version Number	Status/Summary of Revisions Made	Version Date
1.0	Appendix 1 outlined possible example scenarios for adaptive design. Arms A and B included.	August 21, 2020
1.1	• Section 5.1 Inclusion Criteria: Broadened type of SARSCoV2 tests, in accordance with NIH recommendations<br>• Appendix 1: Revised to reflect the master protocol current state of arms<br>• Appendix 1.1: Adds arm C<br>• Appendix 1.2: Adds arm D<br>• Appendix 2: Neuroimaging detailed criteria for hemorrhagic stroke conversion removed to be refined in the event charter<br>• Appendix 2: The method of calculation of ventilator-free days was removed from the description of the primary endpoint, where it is not relevant. The definition will appear in a manual.<br>• Appendices 1 and 3: New exclusion for Arm A for patients who require ICU level of care at screening, based on DSMB review and NHLBI Determination, as of Dec 19. 2020<br>• New exclusion for Arm B for patients who do not require ICU level of care at screening, based on DSMB review and NHLBI Determination, as of Jan 21. 2021<br>• Appendix 3: Added recommendation to enroll patients with elevated d-dimer<br>• Appendices 3, 4: Clarified quality of life assessment on schedule of assessments<br>• Appendix 5: Clarification on blood collection window<br>• Appendix 7 Added for arm C<br>• Section13.3.2: Added investigator *"designee"* to consent process<br>• Appendix 8: Added for arm D	February 01, 2021

**Study Chair**<br>**Clinical Coordinating Center**	Judith Hochman, MD<br>Harold Snyder Family Professor & Associate Director of Cardiology<br>Senior Associate Dean for Clinical Sciences<br>Co-Director, NYU-HHC Clinical and Translational Science Institute<br>NYU School of Medicine<br>530 First Avenue, Skirball 9R<br>New York, NY 10016<br>Tel 212-263-6927<br>Email: judith.hochman@nyumc.org
**Coordinating Center,**<br>**Statistics, Data and Clinical**	Stephen Wisniewski, PhD<br>Professor of Epidemiology<br>Vice Provost for Budget and Analytics<br>University of Pittsburgh<br>Email: STEVEWIS@pitt.edu<br><br>Matthew Neal, MD<br>Roberta G. Simmons Assistant Professor of Surgery<br>Attending Surgeon, Division of Trauma and Acute Care Surgery<br>Assistant Professor of Clinical and Translational Science and Critical Care Medicine<br>Departments of Surgery, Critical Care Medicine, and the Clinical and Translational Science Institute (CTSI), University of Pittsburgh<br>University of Pittsburgh Medical Center<br>F1271.2 PUH 200 Lothrop Street<br>Pittsburgh, PA 15213<br>Tel: 412-647-1158<br>Fax: 412-647-1448<br>Email: nealm2@upmc.edu
**Trial Biostatisticians**	Scott Berry, PhD<br>President, Senior Statistical Scientist<br>Berry Consultants<br>Tel 979-575-6280<br>Email: scott@berryconsultants.com<br><br>Eric Leifer, PhD<br>NHLBI<br>leifere@nhlbi.nih.gov
**NHLBI Representative**	Andrei Kindzelski, MD<br>Email: kindzleskial@nhlbi.nih.gov
**IND**	Waiver
**ClinicalTrials.gov Identifier**	NCT04505774
**WIRB study number**	20202415

Statement of Compliance

In the United States this study will be conducted in accordance with the Code of Federal Regulations on the Protection of Human Subjects (45 CFR Part 46), 21 CFR Parts 50, 56, 312, and 812 as applicable, any other applicable US government research regulations, and institutional research policies and procedures. The International Conference on Harmonisation (“ICH”) Guideline for Good Clinical Practice (“GCP”) (sometimes referred to as “ICH-GCP” or “E6”) and the General Data Protection Regulations (GDPR) will be applied only to the extent that it is compatible with FDA and DHHS regulations.

Outside of the United States this study will be conducted according to local legal and regulatory requirements and regulations, ICH guidelines, and GDPR guidelines as applicable.

The Principal Investigator will assure that no deviation from, or changes to, the protocol will take place without prior agreement from the sponsor and documented approval from the Institutional Review Board (IRB), except where necessary to eliminate an immediate hazard(s) to the trial participants. All personnel involved in the conduct of this study have completed Human Subjects Protection Training.

The signature below provides the necessary assurance that this study will be conducted according to all stipulations of the protocol including statements regarding confidentiality, and according to local legal and regulatory requirements, US federal regulations (if applicable), and ICH E6(R2) GCP guidelines.

Version Date: Feb 01.2021

________________________________ _________________________

Signature of Principal Investigator Date

____

Printed Name of Principal Investigator

____

Name of Facility

____

Location of Facility (City, Country)

Table of Contents

MASTER PROTOCOL SUMMARY 11
1 INTRODUCTION, BACKGROUND INFORMATION AND SCIENTIFIC RATIONALE 13
1.1 BACKGROUND INFORMATION, SIGNIFICANCE AND RELEVANT LITERATURE 13
1.1.1 Adaptive Design 14
1.2 POTENTIAL RISKS & BENEFITS 14
2 STUDY DESIGN 15
2.1 OVERALL STUDY DESIGN 15
2.2 RANDOMIZATION 15
3 OBJECTIVES AND PURPOSE 15
4 STUDY DESIGN AND ENDPOINTS 16
4.1 DESCRIPTION OF STUDY DESIGN 16
4.2 STUDY ENDPOINTS 16
4.2.1 Primary Study Endpoint 16
4.2.2 Secondary Endpoints 16
4.2.3 Additional Study Endpoints 17
4.2.4 Safety Endpoints 17
5 STUDY ENROLLMENT 17
5.1 INCLUSION CRITERIA 17
5.2 EXCLUSION CRITERIA 17
5.3 VULNERABLE SUBJECTS 17
5.4 STRATEGIES FOR RECRUITMENT AND RETENTION 18
5.5 DURATION OF STUDY PARTICIPATION 18
5.6 PARTICIPANT WITHDRAWAL OR TERMINATION 18
5.6.1 Reasons for Withdrawal or Termination 18
5.7 PREMATURE TERMINATION OR SUSPENSION OF STUDY 18
6 STUDY AGENT AND PROCEDURAL INTERVENTION 19
6.1 STUDY AGENTS 19
6.2 DURATION OF THERAPY 19
7 STUDY PROCEDURES AND SCHEDULE 19
7.1 STUDY SCHEDULE 19
7.1.1 Visit 1 and Hospitalization Visits (see arm-specific appendices for details) 20
7.1.2 28 days and/or Date of Hospital Discharge 21
7.2 CONCOMITANT MEDICATIONS, TREATMENTS, AND PROCEDURES 21
7.3 EXPEDITED CRITICAL AND MAJOR EVENT REPORTING 21
7.4 DATA AND SAFETY MONITORING PLAN AND STUDY HALTING RULES 21
8 STATISTICAL CONSIDERATIONS 21
8.1 STATISTICAL AND ANALYTICAL PLANS (SAP) 21
8.2 STATISTICAL MODELING FOR THE PRIMARY ANALYSIS 22
8.3 MODEL PRIORS 23

8.4 ASSESSING EFFECTIVENESS 24
8.5 ANALYSIS DATASETS 24
8.5.1 Safety Analyses 25
8.5.2 Adherence and Retention Analyses 25
8.5.3 Baseline Descriptive Statistics 25
8.5.4 Planned Interim Analysis 25
8.5.5 Safety Review 25
8.5.6 Tabulation of Individual Response Data 25
8.5.7 Exploratory Analyses 26
8.6 SAMPLE SIZE 26
9 MEASURES TO MINIMIZE BIAS 26
9.1 ENROLLMENT/RANDOMIZATION 26
10 RANDOMIZATION 26
11 SOURCE DOCUMENTS AND ACCESS TO SOURCE DATA/DOCUMENTS 26
12 QUALITY ASSURANCE AND QUALITY CONTROL 27
13 ETHICS/PROTECTION OF HUMAN SUBJECTS 27
13.1 ETHICAL STANDARD 27
13.2 INSTITUTIONAL REVIEW BOARD 27
13.3 INFORMED CONSENT PROCESS 27
13.3.1 Consent/Assent and Other Informational Documents Provided to Participants 27
13.3.2 Consent Procedures and Documentation 27
13.4 POSTING OF CLINICAL TRIAL CONSENT FORM 28
13.5 PARTICIPANT AND DATA CONFIDENTIALITY 28
14 DATA HANDLING AND RECORD KEEPING 28
14.1 DATA COLLECTION AND MANAGEMENT RESPONSIBILITIES 28
14.2 STUDY RECORDS RETENTION 28
14.3 PROTOCOL DEVIATIONS 28
14.4 PUBLICATION AND DATA SHARING POLICY 28
15 STUDY FINANCES 28
15.1 FUNDING SOURCE 28
15.2 COSTS TO THE PARTICIPANT 29
16 CONFLICT OF INTEREST POLICY 29
17 REFERENCES 29
APPENDIX 1: MASTER PROTOCOL STATE OF ARMS 31
APPENDIX 1.1 ICU LEVEL OF CARE (SEVERE) COHORT 32
OVERVIEW OF ADDITION OF P2Y12 ANTIPLATELET AGENT ARM C TO EXISTING ARM B FOR ICU LEVEL OF CARE (SEVERE) COHORT 32
APPENDIX 1.2. NON-ICU LEVEL OF CARE (MODERATE) COHORT 33
OVERVIEW OF ADDITION OF P2Y12 ANTIPLATELET AGENT ARM D TO EXISTING ARM A FOR NON-ICU LEVEL OF CARE (MODERATE) COHORT 33
APPENDIX 1.3. CURRENT STATISTICAL MODELING AND ADAPTIONS 34

APPENDIX 2: DEFINITION AND DETERMINATION OF OUTCOMES 36
A2.1 APPROACH TO ASCERTAINMENT AND VERIFICATION OF OUTCOMES 36
A2.2 OUTCOME DEFINITIONS 36
APPENDIX 3: THERAPEUTIC-DOSE ANTICOAGULATION (ARM A) 39
A3.1 THERAPEUTIC DOSE ANTICOAGULATION** 39
A3.2 DISCONTINUATION OF STUDY INTERVENTION: 40
A3.3 STUDY SCHEDULE 40
A3.4 POTENTIAL RISKS & BENEFITS 41
A3.4.1 Known Potential Risks 41
A3.4.2 Known Potential Benefits 41
A3.5 STUDY ENROLLMENT 42
A3.5.1 Inclusion Criteria 42
A3.6 EVENT ADJUDICATION 42
A3.7 SAFETY ANALYSES 42
A3.8 STATISTICAL ANALYSES 42
APPENDIX 4: PROPHYLACTIC DOSE ANTICOAGULATION (ARM B) 44
A4.1 PROPHYLACTIC DOSE ANTICOAGULATION* 44
A4.2 DISCONTINUATION OF STUDY INTERVENTION 44
A4.3 STUDY SCHEDULE 45
A4.4 POTENTIAL RISKS & BENEFITS 46
A4.4.1 Known Potential Risks 46
A4.4.2 Known Potential Benefits 46
A4.5 STUDY ENROLLMENT 46
A4.5.1 Inclusion Criteria 46
A4.5.2 Exclusion Criteria 46
A4.6 EVENT ADJUDICATION 47
A4.7 SAFETY ANALYSES 47
A4.8 STATISTICAL ANALYSES 47
A4.9 REFERENCES 47
APPENDIX 5: ACTIV-4 BLOOD SAMPLING – PROPOSED SAMPLES AND TIMES FOR SITES PARTICIPATING IN MECHANISTIC STUDIES AND BIOREPOSITORY 48
A5.1 INPATIENT SAMPLING 48
A5.2 SAMPLE PROCESSING 48
A5.3 BIOREPOSITORY/CENTRAL LAB 49
APPENDIX 6: ADDITIONAL DATA INCLUSION FROM OTHER TRIALS MERGED UNDER ACTIV-4 PLATFORM 50
APPENDIX 7: BACKGROUND AND RATIONALE FOR ARM C: PROPHYLACTIC-DOSE ANTICOAGULATION, PLUS P2Y12 INHIBITOR FOR ICU LEVEL OF CARE (SEVERE) COHORT 51
A7.1 BACKGROUND AND RATIONALE FOR ARM C 51
A7.2. ELIGIBILITY CRITERIA FOR ARM C 51
A7.2.1 Inclusion Criteria for Arm C 51
A7.2.2 Exclusion Criteria for Arm C 51
A7.3 STUDY AGENTS 52
A7.3.1. Prophylactic Dose Anticoagulation 52*

A7.3.2. P2Y12 Inhibitor *53*
A7.3.3. Participants previously taking aspirin before randomization *53*
A7.4 DURATION OF TREATMENT 53
A7.5 DISCONTINUATION OF STUDY INTERVENTION 54
A7.6 STUDY SCHEDULE 54
A7.7 POTENTIAL RISKS & BENEFITS 55
A7.7.1 Known Potential Risks *55*
A7.7.2 Known Potential Benefits *55*
A7.8 EVENT ADJUDICATION 56
A7.8 SAFETY ANALYSES 56
A7.9 Statistical Analyses *56*
A7.13 REFERENCES 56

APPENDIX 8: THERAPEUTIC-DOSE ANTICOAGULATION, PLUS P2Y12 INHIBITOR (ARM D) FOR NON-ICU LEVEL OF CARE (MODERATE) COHORT 58

A8.1 BACKGROUND AND RATIONALE FOR ARM D 58
A8.2 ARM D ELIGIBILITY 58
A8.2.1 Inclusion Criteria *58*
A8.2.2 Exclusion Criteria for Arm D *59*
A8.3 STUDY AGENTS 59
*A8.3.1. Therapeutic Dose Anticoagulation*** *60*
A8.3.2. P2Y12 Inhibitor *60*
A8.3.3. Participants previously taking aspirin before randomization *61*
A8.4 DISCONTINUATION OF STUDY INTERVENTION: 61
A8.5 STUDY SCHEDULE 61
A8.6 POTENTIAL RISKS & BENEFITS 62
A8.6.1 Known Potential Risks *62*
A8.6.2 Known Potential Benefits *63*
A8.7 EVENT ADJUDICATION 63
A8.8 SAFETY ANALYSES 63
A8.9 STATISTICAL ANALYSES 63
A8.10 REFERENCES 63

List of Abbreviations

AE	Adverse Event/Adverse Experience
ARDS	Acute Respiratory Distress Syndrome.
AT	Arterial Thrombosis
BMI	Body mass index
CFR	Code of Federal Regulations
CHF	Congestive Heart Failure
CrCl	Creatinine Clearance
COVID-19	Coronavirus Disease
CRF	Case Report Form
CSOC	Clinical Study Oversight Committee
DCC	Data Coordinating Center
DHHS	Department of Health and Human Services
DIC	Disseminated Intravascular Coagulation
DSMB	Data and Safety Monitoring Board
DVT	Deep Vein Thrombosis
ECMO	Extracorporeal Membrane Oxygenation
eGFR	Estimated Glomerular Filtration Rate
FDA	Food and Drug Administration
FFR	Federal Financial Report
FWA	Federal Wide Assurance
GCP	Good Clinical Practice
GI	Gastrointestinal
HFNO	High-flow Nasal Oxygen
HIPAA	Health Insurance Portability and Accountability Act
HIT	Heparin Induced Thrombocytopenia
ICF	Informed Consent Form
ICH	International Conference on Harmonisation
ICMJE	International Committee of Medical Journal Editors
IRB	Institutional Review Board
ISM	Independent Safety Monitor
ISTH	International Society on Thrombosis and Haemostasis
ITT	Intent to Treat
IV	Invasive Ventilation

KDIGO	Kidney Disease Improving Global Outcomes
LAR	Legally Authorized Representative
LOS	Length of Stay
MI	Myocardial Infarction
MOP	Manual of Procedures
N	Number (typically refers to participants)
NIH	National Institutes of Health
NIV	Non-invasive ventilation
OHRP	Office for Human Research Protections
OHSR	Office of Human Participants Research
OSFD	Organ Support Free Days
PE	Pulmonary Embolism
PI	Principal Investigator
PRBC	Packed Red Blood Cells
PTT	Partial Thromboplastin Time
QA	Quality Assurance
QC	Quality Control
SAE	Serious Adverse Event/Serious Adverse Experience
sICH	Symptomatic Intracranial or Intracerebral Hemorrhage
SOC	Standard of Care
SOP	Standard Operating Procedure
US	United States
VTE	Venous thromboembolism
WHO	World Health Organization

Master Protocol Summary

Title	A Multicenter, Adaptive, Randomized, Open Label Controlled Platform Trial of the Safety and Efficacy of Antithrombotic Strategies in Hospitalized Adults with COVID-19
Short Title	ACTIV-4 ACUTE
Brief Summary	This is a randomized, open label, adaptive platform trial to compare the effectiveness of antithrombotic strategies for prevention of adverse outcomes in COVID-19 positive inpatients
Objectives	**1.** To determine the most effective antithrombotic strategy for increasing the number of days free of organ support and reducing death.<br><br>**2.** To determine the most effective antithrombotic strategy on the composite endpoint of death, deep vein thrombosis (DVT), pulmonary embolism (PE), myocardial infarction (MI), ischemic stroke, or other systemic arterial thrombosis (AT).<br><br>**3**. To assess the safety of antithrombotic strategies through the endpoint of major bleeding as defined by ISTH.<br><br>**4.** To compare the effect of antithrombotic strategies on the endpoint of all-cause mortality in the study population.<br><br>Assessment of efficacy and safety will yield information of the net clinical benefit of different antithrombotic strategies in the study population. It will also yield information on outcomes specific to under-represented minority populations, specifically African- and Hispanic-descent persons.
Methodology	Adaptive Randomized Platform Trial

Endpoints	Primary Endpoint: 21 Day Organ Support Free Days, which is defined as the number of days that a patient is alive and free of organ support through the first 21 days after trial entry. Organ Support is defined as receipt of invasive or non-invasive mechanical ventilation, high flow nasal oxygen, vasopressor therapy, or ECMO support, with death at any time (including beyond 21 days) during the index hospitalization assigned -1 days.<br><br>Key Secondary Endpoint: Composite endpoint of death, pulmonary embolism, systemic arterial thromboembolism, myocardial infarction, or ischemic stroke at hospital discharge or 28 days, whichever occurs first.<br><br>Other Secondary Endpoints: Composite endpoint of death, deep vein thrombosis, pulmonary embolism, systemic arterial thromboembolism, myocardial infarction, or ischemic stroke at hospital discharge or 28 days, whichever occurs first. Acute kidney injury defined by KDIGO criteria, Individual endpoints comprising the key secondary endpoint, death during hospitalization, 28 Day Ventilator-Free Days, 28 Day Vasopressor Free Days, 28 Day Renal Replacement Free Days, WHO clinical scale, 28 Day Hospital Free Days, 28 day organ support free days, and all-cause mortality at 90 days.<br><br>Primary Safety Endpoint: Major bleeding (as defined by the ISTH)<br>Secondary Safety Endpoint: Confirmed heparin induced thrombocytopenia (HIT)
Study Duration	Approximately 1 year
Participant Duration	Hospital duration with periodic contact at post-discharge, including at 90 days, with potential contact up to 1 year
Duration of assigned treatment strategy	During hospitalization (unless otherwise specified in description of arm)
Population	Adult patients hospitalized for COVID-19
Study Sites	Approximately 400 sites
Number of participants	The sample size is described in each arm-specific appendix.
Description of Study Agents	Randomized arms- see appendix<br><br>This platform trial allows for multiple therapies to be investigated in this trial over time. The trial is governed by a Master Protocol that describes the trial design, endpoint collection, primary endpoint, and inclusion/exclusion criteria. Different therapies, referred to as arms, are detailed in arm-specific appendices. These arm-specific appendices work in a modular fashion as arms are removed and added to the platform trial.
Key Procedures	Observation during hospitalization, contact at 90 days post-enrollment, and collection of standard of care laboratory results. Ancillary biobanking will be completed in consenting patients at capable centers.

Statistical Analysis	Inferences in this trial are based on a Bayesian statistical model, which considers the variation in outcomes by site, disease state, time, and arm of the trial. The specific analyses for each arm, including interim analysis schedule, are specified in each arm-specific appendix.

1 Introduction, Background Information and Scientific Rationale

1.1 Background Information, Significance and Relevant Literature

The severe acute respiratory syndrome coronavirus 2, which causes the highly contagious coronavirus disease 2019 (COVID-19), has resulted in a global pandemic.

The clinical spectrum of COVID-19 infection is broad, encompassing asymptomatic infection, mild upper respiratory tract illness, and severe viral pneumonia with respiratory failure and death. The risk of thrombotic complications is increased, even as compared to other viral respiratory illnesses, such as influenza (1-4). A pro-inflammatory cytokine response as well as induction of procoagulant factors associated with COVID-19 has been proposed to contribute to thrombosis as well as plaque rupture through local inflammation (5). Patients with COVID-19 are at increased risk for arterial and vein thromboembolism(6), with high rates observed despite thromboprophylaxis (7). Autopsy reports have noted micro and macro vascular thrombosis across multiple organ beds consistent with an early hypercoagulable state (8).

Notably, in COVID-19, data in the U.K. and U.S. document that infection and outcomes of infection are worse in African and Hispanic descent persons than in other groups. The reasons for this are uncertain.

Viral Infection and Thrombosis

A large body of literature links inflammation and coagulation; altered hemostasis is a known complication of respiratory viral infections (9-11). Procoagulant markers are severely elevated in viral infections. Specifically, proinflammatory cytokines in viral infections upregulate expression of tissue factor, markers of thrombin generation, platelet activation, and down-regulate natural anticoagulant proteins C and S (11).

Studies have demonstrated significant risk of deep venous thrombosis (DVT), pulmonary embolism (PE), and myocardial infarction (MI) associated with viral respiratory infections (10,12). In a series of patients with fatal influenza H1N1, 75% had pulmonary thrombi on autopsy (a rate considerably higher than reported on autopsy studies among the general intensive care unit population (13). Incidence ratio for acute myocardial infarction in the context of Influenza A is over 10 (14). Severe acute respiratory syndrome coronavirus-1 (SARS CoV-1) and influenza have been associated with disseminated intravascular coagulation (DIC), endothelial damage, DVT, PE, and large artery ischemic stroke (11,15). Obi et al. found that patients with Influenza H1N1 and acute respiratory distress syndrome (ARDS) had a 23.3-fold higher risk for pulmonary embolism, and a 17.9-fold increased risk for deep vein thrombosis (16). Compared to those treated with systemic anticoagulation, those without treatment were 33 times more likely to suffer a VTE (16).

Thrombosis, both microvascular and macrovascular, is a prominent feature in multiple organs at autopsy in fatal cases of COVID-19 (8). Thrombosis may thus contribute to respiratory failure, renal failure, and hepatic injury in COVID-19. The number of megakaryocytes in tissues is higher than in other forms of ARDS, and thrombi are platelet-rich based on specific staining. Thrombotic stroke has been reported in young COVID-19 patients with no cardiovascular risk factors (17). Both arterial

and venous thrombotic events have been seen in increasing numbers of hospitalized patients infected with COVID-19. The incidence of thrombosis has ranged from 10 to 30% in hospitalized patients; however, this varies by type of thrombosis captured (arterial or vein) and severity of illness (ICU level care, requiring mechanical ventilation, etc.).

D-dimer, a biomarker of fibrin formation and degradation, is elevated in conditions associated with thrombosis, and has been strongly associated with increased mortality among patients with COVID-19 (1, 2, 3, 6, 7). In a retrospective analysis of 191 patients with COVID-19, Zhou et al. found that non-survivors were more likely to have D-dimer levels > 1 ug/mL than survivors (81% v 24%) (5). Similarly, in a study of 183 patients, Tang et al. noted that non-survivors had significantly higher D-dimer values on admission than survivors (2.12 v 0.61 ug/mL, P < 0.001) (6). In a retrospective study, patients with COVID-19 and D-dimer values > 6-fold upper limit of normal had lower 28-day mortality when treated with prophylactic anticoagulation compared with no anticoagulation (32.8% v 52.4%, p=0.017) (8). Data suggest a strong association between D-dimer and the outcomes of ICU intubation and all-cause mortality, and the association between D-dimer and (1) mortality, (2) critical illness, (3) acute kidney injury, and (4) thrombotic risk is increased at a D-dimer between 1X to 2X the upper limit of normal. Thrombosis is also increased in those with elevated inflammation indexed by C-reactive protein level (20). Preliminary data suggest that platelet activity is increased in COVID-19(18) and that biomarkers of platelet activity correlate with the incidence of death or thrombosis in hospitalized patients with COVID-19. Platelet-fibrin thrombi have been observed in alveolar capillaries, where they may affect gas exchange (8), and in the renal peri-tubular capillaries, where they may contribute to acute tubular necrosis and renal dysfunction. Consistently, autopsy findings demonstrate an increase in the number of circulating megakaryocytes outside the bone marrow and lung. Finally, thrombotic events have been noted – even among patients treated with full dose anticoagulation.

There may be racial and ethnic differences in response to COVID 19 infection. It is hypothesized that antithrombotic interventions being tested will benefit all patients, including those who are disproportionately affected. (21–25, 26).

The ACTIV-4 ACUTE investigators postulate that an antithrombotic regimen will improve clinical outcomes in COVID-19 patients. This protocol intends to define the optimal regimen in an adaptive randomized trial of patients hospitalized with COVID-19 at risk for adverse clinical outcomes. The primary outcome will be the number of days free of organ support within 21 days after randomization. This primary outcome was selected because thrombosis is thought to contribute to the pathogenesis of multi-organ failure in COVID-19, because it is pragmatic and yet clinically relevant, and to align with ongoing studies that may or may not involve antithrombotic therapy, in a time frame relevant to acute illness. Organ support free days is defined by days in which patient is not on invasive or non-invasive mechanical ventilation, high flow nasal oxygen, vasopressor therapy, or ECMO support (see Appendix 2), with death assigned the value of –1 days.

1.1.1 Adaptive Design

This platform trial will have multiple arms, which may be dropped or added as the platform trial progresses. Sample size will be flexible: the trial will be stopped for efficacy or futility based on pre-determined statistical thresholds as defined in the arm-specific appendices. Each arm will have an adaptive component for determinations of futility or success.

1.2 Potential Risks & Benefits

See arm-specific Appendices for details

2 Study Design

2.1 Overall Study Design

This trial design is built as a process – with the possibility of multiple interventions being investigated. The trial is designed to be flexible, and these flexible aspects are planned as part of the protocol. This trial may incorporate a flexible number of interventions, and the number of interventions may evolve as the science evolves. Intervention arms will be added or dropped based on criteria defined in arm-specific appendices. Co-enrollment in other trials is permitted as long as the other trial does not test agents with antithrombotic properties and there is no other scientific contraindication.

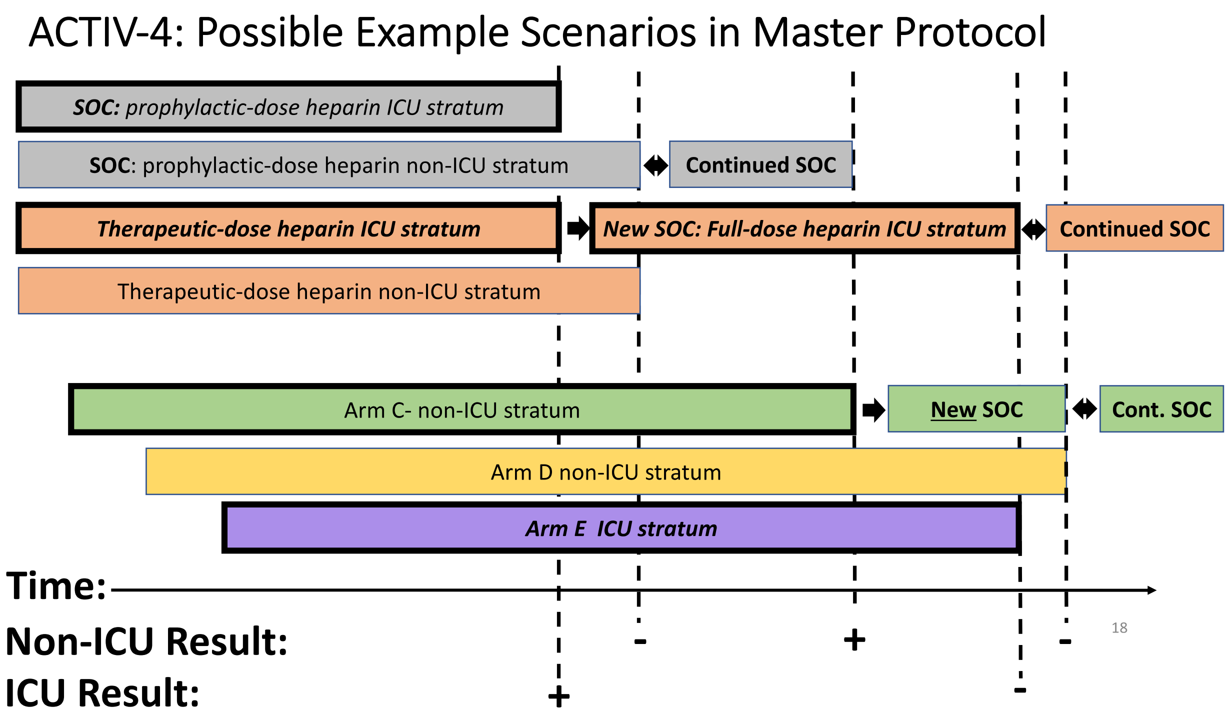

2.2 Randomization

Randomization assignments are at the participant level and are assigned at baseline. Randomization will be stratified by enrolling site and may also be stratified by severity of illness and/or other arm-specific criteria. In general, allocation will be equally distributed across arms for which the participant is eligible, but may be altered with future arm-specific appendices.

3 Objectives and Purpose

The overarching objective of this adaptive platform design is to iteratively learn which antithrombotic strategy is the best for reducing the primary, secondary, and safety outcomes. Additional alternative strategy(-ies) will be compared to the current standard of care arm, which may trigger new standard of care designated arms as appropriate based on interim analysis results and evolving literature. This process will continue until no new strategies replace the standard of care or potential options for additional antithrombotic interventions are exhausted.

4 Study Design and Endpoints

4.1 Description of Study Design

This trial design is built as a process – with the possibility of multiple interventions being investigated. This is an open label randomized trial of patients hospitalized for COVID-19 who are assigned to different antithrombotic regimens.

4.2 Study Endpoints

4.2.1 Primary Study Endpoint

21 Day Organ-Support free-days. The primary endpoint is the number of days that a patient is alive and free of organ support through 21 days after trial entry. Organ support is defined by receipt of invasive or non-invasive mechanical ventilation, high flow nasal oxygen, vasopressor therapy, or ECMO support. If the patient dies at any time (including beyond 21 days) during the index hospital stay, they are assigned the worst possible score of –1.

4.2.2 Secondary Endpoints

- **Key Secondary Endpoint**: A composite endpoint of death, pulmonary embolism, systemic arterial thromboembolism, myocardial infarction, or ischemic stroke during hospitalization or at 28 days after enrollment (whichever is earlier)

Other Secondary Endpoints:

- A composite endpoint of death, deep vein thrombosis, pulmonary embolism, systemic arterial thromboembolism, myocardial infarction, or ischemic stroke during hospitalization or at 28 days after enrollment (whichever is earlier)
- 28 Day Hospital free days (non-ICU level patients)
- 28 Day Ventilator-Free Days (ICU level patients)
- 28 Day Vasopressor-Free Days (ICU level patients)
- 28 Day Renal Replacement Free Days
- Hospital readmission within 28 days
- Acute kidney injury as defined by KDIGO criteria
- Deep vein thrombosis
- Pulmonary embolism
- Systemic arterial thrombosis or embolism
- Myocardial infarction
- Ischemic stroke
- Use of extracorporeal membrane oxygenation (ECMO) support
- Mechanical circuit (dialysis or ECMO) thrombosis
- All-cause mortality at 28 days
- Organ support free days at 28 days
- All-cause mortality during initial hospitalization (includes death after 28 days)
- WHO ordinal scale (peak scale over 28 days, scale at 14 days, and proportion with improvement by at least 2 categories compared to enrollment, at 28 days)
- All-cause mortality at 90 days

4.2.3 Additional Study Endpoints

- Individual endpoints of the thrombotic endpoint
- Length of Hospital stay
- Exploratory endpoints (subset of sites)
  - Cardiac injury (e.g., troponin)
  - Trajectories of biomarkers related to COVID-19
  - DIC

See arm-specific Appendices for additional tertiary endpoints of interest specific to arm.

4.2.4 Safety Endpoints

- Major Bleeding (as defined by the ISTH)
- Symptomatic intracranial or intracerebral hemorrhage (evaluated as a separate endpoint from other major bleeding) (19)
- Confirmed Heparin induced thrombocytopenia (laboratory confirmed by anti-PF4 test or Serotonin Release Assay (SRA))

5 Study Enrollment

5.1 Inclusion Criteria

In order to be eligible to participate in this study, an individual must meet all of the following criteria:

- ≥ 18 years of age
- Hospitalized for COVID-19*
- Enrolled within 72 hours of hospital admittance or 72 hours of positive COVID test
- Expected to require hospitalization for > 72 hours
- See arm-specific Appendices for additional criteria and details

*It is strongly recommended to confirm SARSCoV2 with a positive PCR or other commercial or public health assay prior to randomization. At centers where there is a delay in confirming the diagnosis, a sufficiently high clinical suspicion is sufficient to proceed with randomization as long as confirmation is expected within 24 hours.

5.2 Exclusion Criteria

- Imminent death
- Requirement for chronic mechanical ventilation via tracheostomy prior to hospitalization
- Pregnancy
- See arm-specific appendices for additional criteria and details.

5.3 Vulnerable Subjects

Critically ill patients with COVID-19 may not have capacity to provide consent. This trial will include participants who have no capacity to consent only if their legal proxy is able to consent on their behalf. It has become increasingly apparent that individuals with COVID-19 are at risk for thrombotic (and bleeding) events. Patients without the capacity to consent for themselves will have a potential for direct benefit by being part of the trial.

Participation in this trial is expected to facilitate careful monitoring of both thrombotic and bleeding endpoints, which may benefit participants.

Capacity assessment will be conducted by the treating physician or an independent medical provider with appropriate expertise based on the standard clinical assessment of capacity and communicated to the study team. Surrogate consent will be provided by the subject's Legally Authorized Representative as defined by local policies and state/country regulations.

Consent will be obtained from the LAR before any study related procedures begin. Participants' capacity will be monitored throughout the study by working with the treatment team. Once the participant regains the capacity to consent, they will be informed of their participation in the study and will have an opportunity to withdraw from further participation in the study. The enrollment of patients without capacity is important because critically ill patients, especially those who are not ambulatory, are at higher risk of developing clotting complications.

5.4 Strategies for Recruitment and Retention

Listings of patients admitted to the participating sites with COVID-19 may be reviewed for eligibility by the study team, to identify and recruit potential participants, until study enrollment goals have been met. The study team should communicate with the inpatient care team. All treating physicians will be informed of the study and will have the option to advise of any conditions that would preclude any individual patient being approached.

5.5 Duration of Study Participation

Duration of study participation is, 90 days from enrollment. Participants may be contacted for follow-up for approximately one year.

Total Number of Participants

The total sample size for the Platform trial is not pre-determined. The sample size for each arm will be set in the arm-specific appendix and will incorporate an adaptive design. There will be interim monitoring to allow early stopping for futility, efficacy, or safety. If one strategy proves to be efficacious, then this strategy may become the reference arm for comparison(s) with new experimental treatment(s). New arms can be introduced according to scientific and public health needs. Some arms may not relate solely to antithrombotic therapy.

5.6 Participant Withdrawal or Termination

5.6.1 Reasons for Withdrawal or Termination

Participants are free to withdraw from participation in the study at any time upon request. Discontinuation of a study agent, regardless of the reason, e.g. patient or physician request, or adverse event, does not constitute study withdrawal. Patient data will still be collected as planned and analyzed as intent to treat unless the participant withdraws consent for continued follow-up. An investigator may terminate participation in the study if:

- Any situation occurs such that continued participation in the study would not be in the best interest of the participant

5.7 Premature Termination or Suspension of Study

All deaths and DSMB-specified severe adverse events within the study period will be reviewed by the DSMB. The decision to stop or suspend the study, or an arm of the study, will be made by the DSMB after considering the totality of the data and the benefit-risk of continuing the study.

This study, or an arm of the study, may be temporarily suspended or prematurely terminated if there is sufficient reasonable cause.

Circumstances that may warrant termination or suspension include, but are not limited to:

- Determination of unexpected, significant, or unacceptable risk to participants in a strategy, such as excess mortality and/or major bleeding (this will be determined by the oversight data safety monitoring plan)
- Demonstration of efficacy or lack thereof that would warrant stopping
- Insufficient compliance to protocol requirements
- Data that are not sufficiently complete and/or evaluable
- Determination of futility

The study may resume once concerns about safety, protocol compliance, data quality are addressed and satisfy the sponsor, IRB and/or FDA.

6 Study Agent and Procedural Intervention

6.1 Study Agents

Each arm in this platform trial will include different treatment strategies. Information about the treatment strategies for a given arm can be found in the arm -specific appendices.

6.2 Duration of Therapy

Once participants are randomized to a treatment strategy (arm), they will remain on treatment for the duration specified by the relevant appendix. However, if a participant randomized to one arm develops an indication for a different strategy (e.g., thrombotic event, worsening clinical status), the participant will be treated based on institutional guidelines with any measures required by local clinical judgment.

7 Study Procedures and Schedule

7.1 Study Schedule

Activity	Screening/ Enrollment	Hospital Duration	28 days and/or hospital discharge***	90-days post randomization
**Eligibility**				
Consent	X			
Demographic and Medical History	X			
Assessment of Inclusion/Exclusion criteria	X			
Self-reported race/ethnicity and gender	X			
**Study Drug Administration**				
Randomization	X			
Study treatment	X	X		

**Study Procedures**				
Height	X			
Weight	X			
Vital signs	X			
Concomitant medications	X	X		
WHO ordinal assessment	X	X	X	X
Outcomes Assessment		X	X	X
**SOC Laboratory Assessments**				
Chemistry panel	X	X		
Hematology panel	X	X		
D-dimer*	X			
Blood Group**	X			

See arm-specific appendices for additional measures

*D-dimer is strongly recommended for measurement in all participants as close to the time of randomization as feasible.
**Blood group will come from hospital record or self report if available. Biospecimens see appendix 4.
***Assessments indicated in the table above will be ascertained at discharge, or at 28 days, whichever comes first. Participants must be followed for vital status until discharged from the hospital or another care facility (if transferred on organ support) up to 90 days. To maximize retention, participants will be contacted intermittently (e.g. at one and two months post-discharge)

Laboratory Procedures/Evaluations
See arm specific appendices.

All analyses will be performed on SOC labs and procedures done for usual care. The standard operating procedures for samples to be collected for research purposes are included as Appendix 5. All research samples will be timed with clinical lab draws to limit provider exposure. Collection of research samples as outlined in Appendix 5 is strongly encouraged where safe and feasible.

7.1.1 Visit 1 and Hospitalization Visits (see arm-specific appendices for details)

Visit 1 (Screening and Randomization)

1. Informed consent obtained
2. Assessment of inclusion/exclusion criteria assessed
3. Screening, consisting of reviewing participant medical history and information in their chart such as height, weight, vital signs, and normal clinically performed laboratory assessments, including pregnancy test for all women of childbearing age.
4. If confirmed eligible, following randomization, initiation of treatment with the assigned strategy

Hospitalization Visits

1. Recording of specifics of study treatment according to assigned arm
2. Laboratory assessments as part of standard of care
3. Daily WHO ordinal assessment
4. Ongoing daily outcomes and safety assessment

7.1.2 28 days and/or Date of Hospital Discharge

1. Recording of outcomes and safety assessments as reported by participant or observed by investigator
2. WHO Ordinal Assessment
3. Recording of vital status and ascertainment of events
4. Recording of participant's adherence to treatment strategy, if patient is in hospital

These assessments will be ascertained at discharge, or at 28 days, whichever comes first. Participants must be followed for vital status until discharged from the hospital or another care facility (if transferred on organ support) up to 90 days.

Participants may be contacted by a research contact and/or by the participating hospital study team periodically for longer term follow-up for approximately a year. To maximize retention, participants will be contacted intermittently (e.g. at one and two months post-discharge). Discharge visits must be completed.

7.2 Concomitant Medications, Treatments, and Procedures

Concomitant medications taken during study participation will be recorded on the case report forms (CRFs). Concomitant medications to be recorded are:

- Other antithrombotics (e.g., aspirin and other antiplatelet agents)
- Any medications used for the treatment of COVID-19 infection (e.g., remdesivir, steroids, IL-6 inhibitor such as tocilizumab)
- Others specified in arm-specific appendices

7.3 Expedited Critical and Major Event Reporting

All efficacy and safety outcome events will be assessed and documented in the participants' study records. The ACTIV-4 Platform will have a uniform policy for reporting adverse events to ensure that all events are assessed quickly and are submitted to the DSMB, IRB(s), and other groups as needed (e.g., FDA), following each group's reporting guidelines and timelines. Events meeting the independent DSMB-specified criteria will be reported immediately and within the time frames specified by the DSMB.

Sites are required to follow their local reporting guidelines.

7.4 Data and Safety Monitoring Plan and Study Halting Rules

The ACTIV-4 Platform will have a uniform Data and Safety Monitoring Plan, encompassing all research carried out within the Platform.

8 Statistical Considerations

8.1 Statistical and Analytical Plans (SAP)

There will be a formal Statistical Analysis Plan (SAP) and each arm added to the trial will have its own arm-specific SAP. This will include the primary analysis, the primary comparison, futility and success rules, and interim analysis schedule. The SAP will be created prior to the first interim analysis for the study and each arm-specific SAP will be created before the first interim analysis for that arm.

8.2 Statistical Modeling for the Primary Analysis

Inferences in this trial are based on a Bayesian statistical model for the ordinal primary outcome, organ-support free-days (OSFD). There is a single Bayesian model for the primary outcome across each arm and subpopulation. The Bayesian model is an ordinal cumulative logistic regression model described below.

Let $Y_i = \{-1, 0, 1, \ldots, 21, 22\}$ denote the ordinal outcome (OSFD) for patient i. The probability of patient i observing y OSFD or less is denoted as $\pi_{iy} = \Pr(Y_i \leq y)$. The parameters in the model are structured so that a value > 0 implies treatment benefit, and hence an odds-ratio > 1 implies treatment benefit. In this section we describe the generic model for the study, but arm-specific appendices may vary in its modeling assumptions. The generic primary analysis model is formulated as follows:

$$\log\left(\frac{\pi_{iy}}{1-\pi_{iy}}\right) = \alpha_{y,s} - \left[\nu_{Site,s} + \lambda_{Time,s} + \theta_{a,s:d} + \beta_{Age,s} + \beta_{Sex,s} + \beta_{d}\right]$$

1. The "subtype" variable, *s*, corresponds to the two patient subgroups defined by disease severity:
    a. subtype = 1 is non-ICU level care
    b. subtype = 2 is ICU-level care
2. The d-dimer level for a patient, d, is classified for a patient as
    a. d=1 is a low or unknown d-dimer level
    b. d=2 is a high d-dimer

The d-dimer level is only used for non-ICU (s=1) patients. We use the notation s:d to imply the parameterization would be s=1, d=1 (non-ICU level care, low d-dimer); s=1, d=2 (non-ICU level care, high d-dimer); and s=2 (ICU care).

3. The "site" variable is the clinical site within the trial. These will be site effects estimated separately within the non-ICU and ICU level of case disease states, but not varying by d-dimer levels.
4. The "time" variable is an indicator of the month of enrollment in the trial, numbered decreasing from the first enrollment to the last enrollment for the analysis. The time effects will be estimated separately within the non-ICU and ICU level of case disease states, but not varying by d-dimer levels.
5. The "arm" the patient is randomized to is labeled as *a*. The effects of arm are modeled by both the disease state and the d-dimer level.
6. The "age" variable is a categorical classification of age as ≤ 39, 40-49, 50-59, 60-69, 70-79, and 80+. The age effects will be estimated separately within the non-ICU and ICU level of case disease states, but not varying by d-dimer levels.
7. The "sex" variable is sex at birth. The sex effects will be estimated separately within the non-ICU and ICU level of case disease states, but not varying by d-dimer levels.

If additional covariates (e.g. race and ethnicity) are added to the model they will by default, unless otherwise specified, vary by disease state, but not d-dimer levels.

The $\alpha_{y,subtype}$ parameters are the baseline rates of the ordinal outcome, which are modeled separately by disease subtype. The additive effects of d-dimer levels are modeled with the β_d.

8.3 Model Priors

The treatment effects for arm *a*, within disease subtype *s* and d-dimer level *d* are modeled with the $\theta_{a,s,d}$ parameters. The β parameters model any covariate effects included in the model. The λ parameters model the effect of time within the pandemic.

The ordinal endpoint rates are modeled using an inverse Dirichlet model where the individual probabilities for the 24 outcomes are based on 1 patient's weight on real-world evidence-based outcomes. The weight for each of the outcomes will be assumed equal.

$$logit(\alpha_{y,s}) \sim Dirichlet(\mathbf{1} * P), where \text{ P} \propto 1$$

The site effects, ν_{Site}, are modeled using a hierarchical model where site is nested within the country of the site:

$$\nu_{Site,s} \sim N(\mu_{country,s}, \tau^2_{country,s}), site = 2, \ldots, N_{Site}$$

$$\mu_{country,s} \sim N(0,1);\ \tau^2_{country,s} \sim IG(0.25, 0.1), s = 1,2$$

A referent site, expected to be the largest enrolling site, will be set such that $\nu_{Site} \equiv 0$. The hyper-parameters of the site hierarchical model are separate by disease state *s*.

The effect of time (*T*) is modeled using a second-order normal dynamic linear model separately by disease state, *s*. The most recent two time periods are modeled as the referent time epochs with the time parameters set to 0. The preceding time epochs are modeled as a normal dynamic linear model as:

$$\lambda_1 = \lambda_2 \equiv 0$$

$$\lambda_3 \sim N(0, 0.15^2)$$

$$\lambda_T - 2\lambda_{T-1} + \lambda_{T-2} \sim N(0, \tau^2_{Time}), T \geq 4$$

The treatment effect parameters are set against a control arm, which will be labeled in the arm-specific appendix. The treatment effect for the control arm, labeled as arm a = 1, will be set to 0 for each of the disease subtype and d-dimer level:

$$\theta_{1,subtype,d} \equiv 0$$

The effect of each treatment arm introduced will typically be modeled hierarchically across disease subtypes and/or d-dimer levels. The modeling of the treatment arms will be specified in appendices.

Any additional covariates included in the model will have independent N(0,1) priors unless otherwise specified.

8.4 Assessing Effectiveness

The treatment effect parameters, θ, represent the log-odds ratio, of the treatment, for the cumulative logistic for the ordinal model. In this parametrization an odds ratio > 1, or a log-odds ratio > 0, signifies improved outcomes relative to the referent control treatment. The odds-ratio parameter $\exp(\theta)$, labeled OR, will be used to summarize the treatment effect relative to control or $\exp(\theta_{a_1} - \theta_{a_2})$ for the odds-ratio between arms a_1 and a_2. The posterior mean, median, standard deviation, and 95% credible intervals for the odds-ratio will be used to summarize relative treatment effects.

The posterior probability that an arm, a_1, is superior to another arm, say, a_2, is:

$$\Pr(\theta_{a_1} > \theta_{a_2}).$$

This probability will be used for triggers of superiority of one arm to another arm.

The posterior probability that an arm, a_1, is superior to another arm, say, a_2, by a specified difference on the odds-ratio scale is:

$$\Pr(\exp(\theta_{a_1}) > \exp(\theta_{a_1}) + \delta).$$

This probability will typically be used for futility. If the probability is small that a treatment has benefit above a control of some specified amount (δ), the arm may be dropped for futility.

8.5 Analysis Datasets

The intention-to-treat (ITT) analysis dataset will be the source of data for primary analyses. This will include all randomized participants regardless of actual receipt or compliance with therapy. The safety analysis set will consist of all participants who received at least one dose of study

medication. The per protocol analysis will be conducted based on adherence to assigned treatment; this dataset will support sensitivity analyses to complement the primary ITT analyses.

The ITT group for an arm consists of the participants that were randomized in the platform that were eligible to be randomized to that arm. This may vary from the platform ITT population, which consists of all participants randomized.

Participants who are randomized to receive one strategy may in fact be treated with another strategy based on health status and provider discretion. Exploratory analyses will estimate the causal effect of the treatment for these participants using marginal structural modelling techniques. These techniques use inverse probability weighting methods that are based on patient-level covariates to create comparable groups for the analysis.

8.5.1 Safety Analyses

Monitoring for safety will be conducted continuously. For each arm-specific appendix potential adverse events of importance will be identified. A Bayesian monitoring rule will be used to summarize the adverse event rates across all arms for the adverse events of importance within each arm-specific appendix. A Bayesian prior distribution of a beta (0.1, 0.9) will be used to model the likelihood of each adverse event of importance. For each adverse event of importance, the posterior mean event rates, the posterior mean of the difference between each arm, and the 95% credible intervals for the risk-difference and odds-ratio will be summarized.

8.5.2 Adherence and Retention Analyses

The primary analysis is by intention to treat. Per protocol analysis will be conducted based on adherence to assigned treatment. For any scheduled follow-up post hospital discharge every effort will be made to recontact participants who are unreachable. Due to the short trial participation timeline, excellent patient retention is anticipated.

8.5.3 Baseline Descriptive Statistics

All variables will be summarized using mean, median, standard deviation, and range (for continuous variables) and frequency (for categorical variables). Treatment groups will be compared with respect to baseline characteristics to verify randomization balance.

8.5.4 Planned Interim Analysis

An independent data safety and monitoring board (DSMB) will review all interim analyses prepared by an unblinded statistical analysis committee.

8.5.5 Safety Review

Monitoring for safety will be conducted continuously. The DSMB will be monitoring safety for each arm-specific appendix. The DSMB monitoring plan includes guidance on stopping specific arms for safety concerns.

8.5.6 Tabulation of Individual Response Data

The composite outcome evaluated will be tabulated and broken down by component (e.g., death, pulmonary embolus, symptomatic DVT, myocardial infarction, etc.). Note that some participants may experience more than one component of the primary endpoint.

8.5.7 Exploratory Analyses

Exploratory analyses will be conducted in a subset of participants on whom additional clinical and basic science assays are performed. These will be descriptive and hypothesis-generating.

8.6 Sample Size

Sample size for the platform trial is not pre-determined. The platform trial will run as long as there is a need and there are investigational arms enrolling. The sample size for each arm will be specified in the arm-specific appendix. Interim analyses for each arm will take place in the platform trial and detailed in the arm-specific appendix. Conclusions of futility or superiority may be drawn specific to a patient subtype. Effort will be taken to conduct all interim analyses at the same time in the platform trial since there is a single Bayesian model of the efficacy of all arms conducted. If one strategy proves to be efficacious, then this strategy may become the reference arm for comparison(s) with new experimental treatment(s). New arms can be introduced according to scientific and public health needs.

Generic sample size calculations for an ordinal endpoint of 21-day OSFD with a maximum sample size of 1000 for an investigational arm, compared to a second control arm with 1000 participants (2000 participants total), yields over 80% power for an odds-ratio change of 1.25 on the OSFD endpoint. An odds ratio of 1.5 has approximately 90% power for 400 participants per arm. An odds-ratio of 2 results in more than 90% power for the first interim analysis of 200 participants per arm. An updated sample size estimate for additional arms is provided in Appendix 1.3.

9 Measures to Minimize Bias

9.1 Enrollment/Randomization

Enrollment

1. Patients hospitalized for COVID-19 are screened daily within the eligibility time window for inclusion/exclusion criteria. Any patient who meets all inclusion criteria and no exclusion criteria will be approached for enrollment.

2. Patients remain in the intention-to-treat group if they meet the criterion for another treatment strategy after randomization.

10 Randomization

Randomization assignments are performed for participants at baseline. Randomization will be equal across all arms a patient is eligible. Randomization stratification will be done by site, and disease subtype (ICU and non-ICU level care) and/or other arm-specific criteria.

11 Source Documents and Access to Source Data/Documents

The ACTIV-4 Platform will have uniform policies describing what source documents are, how to make corrections, and who can access them.

12 Quality Assurance and Quality Control

The ACTIV-4 Platform will have uniform policies for quality assurance at the data entry level and site monitoring.

13 Ethics/Protection of Human Subjects

13.1 Ethical Standard

The investigator will ensure that this study is conducted in full conformity with Regulations for the Protection of Human Subjects of Research codified in 45 CFR Part 46, 21 CFR Part 50, 21 CFR Part 56, and/or the ICH E6.

13.2 Institutional Review Board

The protocol, informed consent form(s), recruitment materials, and all participant materials will be submitted to the IRB for review and approval. Approval of both the protocol and the consent form must be obtained before any participant is enrolled. Any amendment to the protocol will require review and approval by the IRB before the changes are implemented to the study. All changes to the consent form will be IRB approved; a determination will be made regarding whether previously consented participants need to be re-consented.

13.3 Informed Consent Process

13.3.1 Consent/Assent and Other Informational Documents Provided to Participants

Consent forms describing in detail the study agent, study procedures, and risks are given to the participant, and written documentation of informed consent is required prior to starting intervention/administering study product.

A written consent will be sought from every participant via a face to face consenting process or remotely by using an e-consent option as per IRB approved method.

13.3.2 Consent Procedures and Documentation

Informed consent is a process that is initiated prior to the individual's agreeing to participate in the study and continues throughout the individual's study participation. Informed consent will be obtained following institutional COVID policy to protect study staff.

An extensive discussion of risks and possible benefits of participation will be provided to the participants and their families. Consent forms will be IRB-approved and the participant will be asked to read and review the document. The investigator or designee will explain the research study to the participant and answer any questions that may arise. Participants will have the opportunity to carefully review the written consent form and ask questions prior to signing. The participants should have the opportunity to discuss the study with their surrogates or think about it prior to agreeing to participate. The participant will sign the consent document prior to any procedures being done specifically for the study. The participants may withdraw consent at any time throughout the course of the trial. A copy of the signed informed consent document will be provided to participants. The rights and welfare of the participants will be protected by emphasizing to them that the quality of their medical care will not be adversely affected if they decline to participate in this study.

Participants who have no capacity to consent for themselves will have a surrogate consenting process via legally authorized representative.

13.4 Posting of Clinical Trial Consent Form

The informed consent form will be posted on the Federal website after the clinical trial is closed to recruitment, and no later than 60 days after the last study visit by any subject, as required by the protocol.

13.5 Participant and Data Confidentiality

The ACTIV-4 Platform will have uniform policies for protecting the privacy of participants and maintaining confidentiality. These policies will adhere to the requirements of the Health Insurance Portability and Accountability Act of 1996 (HIPAA).

14 Data Handling and Record Keeping

14.1 Data Collection and Management Responsibilities

The ACTIV-4 Platform will have uniform policies for data management.

14.2 Study Records Retention

The ACTIV-4 Platform will have uniform policies for records retention.

14.3 Protocol Deviations

A protocol deviation is any noncompliance with the clinical trial protocol, GCP, or Manual of Procedures (MOP) requirements. The noncompliance may be either on the part of the participant, the investigator, or the study site staff. As a result of deviations, corrective actions are to be developed by the site and implemented promptly.

It is the responsibility of the site PI/study staff to use continuous vigilance to identify and report deviations.

Protocol deviations must be reported to the local IRB per their guidelines. The site PI/study staff is responsible for knowing and adhering to their IRB requirements. Further details about the handling of protocol deviations will be included in the MOP.

14.4 Publication and Data Sharing Policy

The ACTIV-4 Platform will have uniform policies for publications and data sharing.

15 Study Finances

15.1 Funding Source

National Institutes of Health

15.2 Costs to the Participant

Participant health insurance may be billed for the costs of medical care during this study since these expenses would have happened even if the participant were not in the study. If the participant's insurance does not cover these costs or the participant does not have insurance, these costs will be participant's responsibility.

16 Conflict of Interest Policy

The ACTIV-4 Platform will have uniform policies for identifying and disclosing potential conflicts of interest.

17 References

1. Bikdeli, B., et al., COVID-19 and Thrombotic or Thromboembolic Disease: Implications for Prevention, Antithrombotic Therapy, and Follow-Up: JACC State-of-the-Art Review. J Am Coll Cardiol, 2020. 75(23): p. 2950-2973.
2. Klok, F.A., et al., Incidence of thrombotic complications in critically ill ICU patients with COVID-19. Thromb Res, 2020. 191: p. 145-147.
3. Middeldorp, S., et al., Incidence of venous thromboembolism in hospitalized patients with COVID-19. J Thromb Haemost, 2020.
4. Poissy, J., et al., Pulmonary Embolism in COVID-19 Patients: Awareness of an Increased Prevalence. Circulation, 2020.
5. Zhou, F., et al., Clinical course and risk factors for mortality of adult inpatients with COVID-19 in Wuhan, China: a retrospective cohort study. Lancet, 2020. 395(10229): p. 1054-1062.
6. Tang, N., et al., Anticoagulant treatment is associated with decreased mortality in severe coronavirus disease 2019 patients with coagulopathy. J Thromb Haemost, 2020. 18(5): p. 1094-1099.
7. Xu J, W.L., Zhao L, et al. , Risk assessment of venous thromboembolism and bleeding in COVID-19 patients. BMC Pulmonary Medicine 2020.
8. Danzi, G.B., et al., Acute pulmonary embolism and COVID-19 pneumonia: a random association? Eur Heart J, 2020. 41(19): p. 1858.
9. Xie Y, W.X., Yang P, Zhang S, COVID-19 complicated by Acute Pulmonary Embolism. Radiology: Cardiothoracic Imaging, 2020. 2(2):e200067.
10. Beristain-Covarrubias, N., et al., Understanding Infection-Induced Thrombosis: Lessons Learned From Animal Models. Front Immunol, 2019. 10: p. 2569.
11. Clayton, T.C., M. Gaskin, and T.W. Meade, Recent respiratory infection and risk of venous thromboembolism: case-control study through a general practice database. Int J Epidemiol, 2011. 40(3): p. 819-27.
12. Goeijenbier, M., et al., Review: Viral infections and mechanisms of thrombosis and bleeding. J Med Virol, 2012. 84(10): p. 1680-96.
13. Smeeth, L., et al., Risk of deep vein thrombosis and pulmonary embolism after acute infection in a community setting. Lancet, 2006. 367(9516): p. 1075-1079.
14. Harms, P.W., et al., Autopsy findings in eight patients with fatal H1N1 influenza. Am J Clin Pathol, 2010. 134(1): p. 27-35.
15. Kwong, J.C., et al., Acute Myocardial Infarction after Laboratory-Confirmed Influenza Infection. N Engl J Med, 2018. 378(4): p. 345-353.
16. Wong, R.S., et al., Haematological manifestations in patients with severe acute respiratory syndrome: retrospective analysis. BMJ, 2003. 326(7403): p. 1358-62.

17. Obi, A.T., et al., Empirical systemic anticoagulation is associated with decreased venous thromboembolism in critically ill influenza A H1N1 acute respiratory distress syndrome patients. J Vasc Surg Venous Lymphat Disord, 2019. 7(3): p. 317-324.
18. Rapkiewicz, A., et al, Megakaryocytes and platelet-fibrin thrombi characterize multi-organ thrombosis at autopsy in 4 COVID-19: a case series. Lancet EClinical Medicine, 2020; Jun 25;24:100434.
19. Oxley, T.J., et al., Large-Vessel Stroke as a Presenting Feature of Covid-19 in the Young. N Engl J Med, 2020. 382(20): p. e60.
20. Al-Samkari, et al., COVID and Coagulation: Bleeding and Thrombotic Manifestations of SARS-CoV2 Infection. Blood, 2020. Online ahead of print.
21. Centers for Disease Control and Prevention. COVID-19 in Racial and Ethnic Minority Groups. 2020.
22. Price-Haywood EG, Burton J, Fort D and Seoane L. Hospitalization and Mortality among Black Patients and White Patients with Covid-19. *N Engl J Med*. 2020.
23. Robert W. Aldridge, Dan Lewer, Srinivasa Vittal Katikireddi, Rohini Mathur, Neha Pathak, Rachel Burns, Ellen B. Fragaszy, Anne M. Johnson, Delan Devakumar, Ibrahim Abubakar and Haywar A. Black, Asian and Minority Ethnic groups in England are at increased risk of death from COVID-19: indirect standardisation of NHS mortality data [version 1; peer review: 3 approved with reservations]. *Wellcome Open Res*. 2020;5.
24. Niedzwiedz CL, O'Donnell CA, Jani BD, Demou E, Ho FK, Celis-Morales C, Nicholl BI, Mair FS, Welsh P, Sattar N, Pell JP and Katikireddi SV. Ethnic and socioeconomic differences in SARS-CoV-2 infection: prospective cohort study using UK Biobank. *BMC Med*. 2020;18:160.
25. Millett GA, Jones AT, Benkeser D, Baral S, Mercer L, Beyrer C, Honermann B, Lankiewicz E, Mena L, Crowley JS, Sherwood J and Sullivan P. Assessing Differential Impacts of COVID-19 on Black Communities. *Ann Epidemiol*. 2020.
26. Kamin-Mukaz D, Gergi M, Koh I, Zakai NA, Judd SE, Sholzberg M, Bauman Kreuziger L, Freeman K, Colovos C, Cushman M. Biomarkers of COVID-19 coagulopathy and D-dimer in a biracial cohort study [abstract]. Res Pract Thromb Haemost 2020;4:Suppl2.

Appendix 1: Master Protocol State of Arms

This appendix will be updated as arms are added or dropped. The current version and version history appear below.

Version 21AUG2020 1AB: Outlined possible example scenarios for adaptive design. Arms A and B included.
Version 01FEB 2021: Adds Arms C and D (Appendices 7 and 8), implements suspension of Arm A in participants with ICU level of care (severe illness) at the time of randomization due to futility, and suspension of Arm B for non ICU level of care participants (moderate illness) at the time of randomization, due to superiority of Arm A.

See Appendix 1.1 for current state of arms in severely ill participants and 1.2 for current state of arms in moderately ill participants.

Randomization: Randomization assignments are at the participant level, stratified by enrolling site and by ICU level of care vs non-ICU level of care and/or other arm-specific criteria.

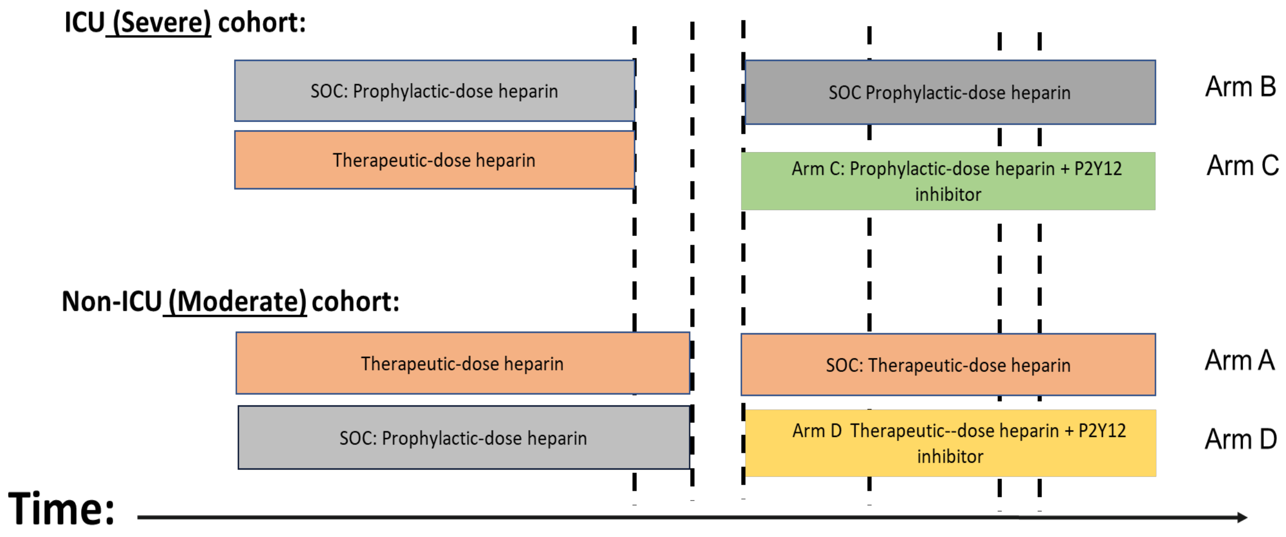

Appendix 1.1 ICU LEVEL OF CARE (SEVERE) COHORT

Overview of Addition of P2Y12 Antiplatelet Agent Arm C to existing Arm B for ICU Level of Care (Severe) Cohort

There are currently two arms in the master protocol for patients in the severe/ICU-level of care cohort.

For the ICU level of care cohort (severe), the treatment arms and arm-specific appendices are:

Arm B: Prophylactic-dose anticoagulation, no P2Y12 inhibitor, Appendix 4
Arm C: Prophylactic-dose anticoagulation, plus P2Y12 inhibitor, Appendix 7

Each participant is randomized equally among each of the arms for which they are eligible.

Participants who ARE receiving ICU level of care at the time of randomization **will be randomized to Arm B or Arm C**.

**Anti-thrombotic Drug**	**Arm B**	**Arm C**
Anticoagulation	Prophylactic-dose	Prophylactic-dose
Antiplatelet: P2Y12 inhibitor	No	Yes

Appendix 1.2. NON-ICU LEVEL OF CARE (MODERATE) COHORT

Overview of Addition of P2Y12 Antiplatelet Agent Arm D to existing Arm A for non-ICU Level of Care (Moderate) Cohort

There are currently two arms in the master protocol for the non-ICU-level of care/moderate cohort.

For the non-ICU level of care (moderate) cohort, the treatment arms and arm-specific appendices are:

Arm A: Therapeutic-dose anticoagulation, no P2Y12 inhibitor, Appendix 3
Arm D: Therapeutic-dose anticoagulation, plus P2Y12 inhibitor, Appendix 8

Each participant is randomized equally among each of the arms for which they are eligible.

Participants in the non-ICU level of care cohort at the time of randomization will be randomized to Arm A or D (See Appendix 8 for eligibility criteria):

**Anti-thrombotic Drug**	**Arm A**	**Arm D**
Anticoagulation	Therapeutic-dose	Therapeutic-dose
Antiplatelet: P2Y12 inhibitor	No	Yes

Appendix 1.3. Current Statistical Modeling and Adaptions

This section presents the current statistical modeling assumptions for the current status of the master protocol (as of 01 FEB 2021).

In the master protocol there will be two subtypes where arms will be compared. Arm C will be compared to Arm B in the ICU (severe) subtype (cohort) and Arm D will be compared to arm A in the non-ICU (moderate) subtype (cohort). In the moderate subtype there will not be analyses by D-dimer levels in the primary analysis.

Adaptations and Conclusions:
Interim analyses are expected to be carried out periodically in this platform with frequency potentially varying by enrollment. The expectation is that interim analyses would occur approximately every 200 patients enrolled to each subtype, no more frequently than monthly.

At each interim analysis the following conclusions could be triggered:

1. In the Moderate State, Arm D can be found superior to Arm A on the primary endpoint. If the posterior probability that Arm D is superior to Arm A on the primary endpoint is at least 99% then the conclusion of superiority of Arm D to Arm A will be made.
2. In the Moderate State, Arm D can be found futile compared to Arm A on the primary endpoint. If the posterior probability that Arm D has at least a 1.2 odds-ratio improvement on the primary endpoint compared to Arm A is less than 5% then the conclusion of futility of Arm D to Arm A will be made.
3. In the Moderate State, Arm D can be found inferior to Arm A on the primary endpoint. If the posterior probability that Arm D is superior to Arm A on the primary endpoint is less than 1% then the conclusion of inferiority of Arm D to Arm A will be made.
4. In the Severe State, Arm C can be found superior to Arm B on the primary endpoint. If the posterior probability that Arm C is superior to Arm B on the primary endpoint is at least 99% then the conclusion of superiority of Arm C to Arm B will be made.
5. In the Severe State, Arm C can be found futile compared to Arm B on the primary endpoint. If the posterior probability that Arm C has at least a 1.2 odds-ratio improvement on the primary endpointcompared to Arm B is less than 5% then the conclusion of futility of Arm C to Arm B will be made.
6. In the Severe State, Arm C can be found inferior to Arm B on the primary endpoint. If the posterior probability that Arm C is superior to Arm B on the primary endpoint is less than 1% then the conclusion of inferiority of Arm C to Arm B will be made.

The primary statistical model in the Master protocol will be used with the following alterations. There will be no modeling of the d-dimer subtypes in the moderate state. In the statistical mode there are two parameters of treatment effects, the effect of adding P2Y12 to therapeutic dose anticoagulation (effect of Arm D compared to Arm A) in the moderate state, $\theta_{D,1}$. In the severe state the effect of adding P2Y12 to the prophylactic-dose anticoagulation (effect of Arm C to Arm B) is $\theta_{C,2}$.
:
The two efficacy parameters, $\theta_{D,1}$ and $\theta_{C,2}$ are modeled hierarchically:

$\theta_{D,1}, \theta_{C,2} \sim N(\mu_{CD}, \tau^2_{CD})$
With hyperpriors

$\mu_{CD} \sim N(0,1)$
and

$$\tau_{CD}^2 \sim IG(0.25,0.10).$$

The start of Arms C and D will trigger the inferential comparison between Arm C to Arm B and Arm D to Arm A, thus the analysis for these comparisons will start when the randomization begins for these arms. No data from before the randomization of arms C and D will be used in the primary analysis for these comparisons. Sensitivity analyses will be conducted comparing patients on Arms A and B before and after the inclusion of arms C and D.

Sample Size Expectations

The relevant comparison with Arms A, B, C and D in the Master protocol is the effect of adding P2Y12 separately in moderate and severe patients in whom there is a different standard of care heparin dose. The sample size calculations present the power for the effect of adding the P2Y12 for an ordinal endpoint of 21-day OSFD with a maximum sample size of 1000 for an investigational arm (adding P2Y12), compared to a control arm with 1000 participants (2000 participants total). This yields over 80% power for an odds-ratio change of 1.25 on the OSFD endpoint. There will be approximately 90% power for an odds ratio of 1.5 with 400 participants per arm. An odds-ratio of 2 results in more than 90% power for the first interim analysis of 200 participants per arm.

Based on these estimates, we expect that 500 patients per arm will be sufficient to reach a conclusion. Thus the sample size is expected to be a maximum of 1000 patients enrolled to Arms C and D with 1000 enrolled to Arms A and B, for 2000 patients total across the 4 arms. If the enrollment is differential across the arms there may be a need for a larger total sample size.

Appendix 2: Definition and Determination of Outcomes

A2.1 Approach to ascertainment and verification of outcomes

Outcomes are assessed locally and will not be centrally adjudicated in this pragmatic trial platform, except as specified in the arm-specific appendix. Outcomes should be assessed by a local investigator or other qualified study team member who is blinded to treatment assignment, using the definitions below.

A2.2 Outcome definitions

21 Day Organ-Support Free-Days (OSFD)

Defined as the number of days that a patient is alive and free of organ support through 21 days after trial entry. Organ support is defined by receipt of invasive or non-invasive mechanical ventilation, high flow nasal oxygen, vasopressor therapy, or ECMO support. If the patient dies at any time (including beyond 21 days) during the index hospital stay, they are assigned the worst possible score of –1.

- Non-invasive mechanical ventilation is defined as BIPAP or CPAP when used for acute respiratory support (the use of BIPAP or CPAP at night or when sleeping for sleep apnea is not considered organ support).
- High Flow Nasal Cannula Oxygen is defined as delivery of oxygen through a system that typically delivers oxygen at 20 to 60 liters per with a titratable FiO2.
- Invasive mechanical ventilation is defined as positive pressure ventilation through endotracheal tube or tracheostomy.
- Vasopressor support includes infusion of any vasopressor or inotropic medication.
- Any patient dying in the acute hospital stay (even if beyond day 21) are assigned 21 Day Organ-Support Free Days of –1.
- If there is intervening time in which a patient is free of organ support but goes back on organ support the intervening time does not count toward the organ support free days endpoint. Only time before organ support and after the last use of organ support are counted as “free days.”
- If a patient was discharged alive without mechanical ventilation prior to Day 21, the patient is assumed to be free of organ support after hospital discharge for the remainder of the 21 days.
- If a patient was discharged alive on mechanical ventilation prior to Day 21, a call to the discharge facility is needed to confirm ventilation status on Day 21 and the last day on mechanical ventilation.

Primary Endpoint

Days free of organ support within 21 days after randomization. Organ support free days (OSFD) is defined as days in which patient is not on invasive or non-invasive mechanical ventilation, high flow nasal oxygen, or vasopressor therapy or ECMO support. If the patient dies at any time (including beyond 21 days) during the index hospital stay, they are assigned the worst possible score of –1.

To be specific about which organ support was affected, secondary outcomes include: ventilator free days, renal replacement free days, vasopressor free days.

Justification for use of OSFD:

- Pragmatic
- Can be calculated from WHO ordinal scores
- Incorporates clinically important need for organ support but also duration of organ support
- No additional data collection is necessary to calculate secondary outcomes of ventilator free days, renal replacement free days, and vasopressor free days to understand which organ support was most impacted
- Incorporates mortality as the worst possible outcome

Secondary Endpoints

Deep vein thrombosis
Deep vein thrombosis will be diagnosed by venous ultrasound or point-of-care ultrasound (POCUS) or other imaging modality and documented in a note, and performed for clinical indications. A positive ultrasound test is defined by a noncompressible or partially noncompressible venous segment and should be reported. Thrombosis may involve the cerebral venous sinus or any venous bed, including the upper extremities. Routine screening for deep vein thrombosis is not recommended. If deep vein thrombosis is diagnosed and treated without imaging due to imaging availability concerns or risk of exposure to SARS CoV-2, this will be classified as probable deep vein thrombosis. Later imaging is preferable in these cases when possible.

Pulmonary embolism
Pulmonary embolism will be confirmed by chest CT with PE protocol, pulmonary angiography or ventilation-perfusion scan. Events may also be defined without this imaging by the care team, as evidenced by, for example, “clot in transit” on echocardiogram. If PE is diagnosed and treated without imaging due to imaging availability concerns or risk of exposure to SARS CoV-2, this will be classified as probable PE. Later imaging is preferable in these cases when possible.

Stroke/ Peripheral Arterial Systemic Thromboembolism
Stroke or systemic embolism as diagnosed by imaging (i.e., head CT, lower extremity CT angiogram) or deemed “highly-likely” by the provider based on physical examination (i.e., acute hemiplegia thought to be due to stroke, acute distal lower extremity hypoperfusion). Systemic thromboembolism may involve the retinal artery, spinal cord or other vascular beds. Classification of ischemic vs. other etiologies is based on neuroimaging. Venous sinus thrombosis will be included in the category of vascular occlusion/ischemic stroke on the venous side. Primary CNS hemorrhage: intracerebral hemorrhage, subarachnoid hemorrhage, subdural hematoma, and rarely epidural hematoma or spinal hematoma. Secondary hemorrhagic stroke: blood associated with an ischemic infarct.

ICU Level of care disease state (severe illness)
Defined as receipt of organ support as defined in the 21-day organ support free days. ICU level of care is defined as being on invasive or non-invasive mechanical ventilation, high flow nasal oxygen, or vasopressor therapy or ECMO support.

Myocardial infarction
Myocardial infarction is defined according to the universal definition of MI, which excludes myocardial injury e.g., isolated elevation of cardiac troponin. MI must include rise and fall of cardiac

troponin above the 99th percentile with at least one of the following: symptoms of acute ischemia, ECG changes consistent with ischemia, new/presumed new wall-motion abnormalities or other imaging evidence of MI, abnormal coronary angiography (e.g. identification of a coronary thrombus).

Acute Kidney Injury
Acute kidney injury after enrollment is defined by KDIGO criteria for Acute Kidney Injury in the setting of not meeting these criteria upon enrollment:

THREE STAGES:

- Stage 1: Serum Cr 1.5–1.9 times baseline, OR ≥ 0.3 mg/dl increase in serum Cr
- Stage 2: Serum Cr 2.0–2.9 times baseline
- Stage 3: Serum Cr ≥ 3.0 times baseline, OR Increase in serum creatinine to ≥ 4.0mg/dl, OR Initiation of renal replacement therapy

Disseminated Intravascular Coagulation (DIC) (Overt) – DIC score ≥ 5

1. Platelet count ≥ 100 K (0); 50–100K (1 point); < 50K (2 points)
2. Elevated D-dimer: no increase (0 points); moderate increase (1 point); severe increase (3 points) according to local criteria.
3. Prolonged PT < 3 seconds (0 points); 3–6 seconds (1 point); ≥ 6 seconds (2 points)
4. Fibrinogen level ≥ 100 (0 points); < 100 (1 point) mg/dL

ISTH Defined Major Bleeding
Bleeding that:

1. Resulted in death,
2. Occurred in a critical organ (intracranial, intraspinal, intraocular, retroperitoneal, intraarticular, intramuscular with compartment syndrome, or pericardial), or
3. Associated with either a decrease in the hemoglobin level of at least 2 g per deciliter or a transfusion of at least 2 units of packed red cells or whole blood.

Symptomatic Intracranial or Intracerebral Hemorrhage (sICH)
sICH is defined as any acute extravasation of blood into the brain parenchyma, subarachnoid space, subdural space, or epidural space as demonstrated by imaging or autopsy, associated with any clinical deterioration or death

WHO ordinal scale for clinical improvement (https://www.who.int/blueprint/priority-diseases/key-action/COVID-19_Treatment_Trial_Design_Master_Protocol_synopsis_Final_18022020.pdf)

Patient State	Score	Descriptor
Uninfected	0	No clinical or virological evidence of infection
Ambulatory	1	No limitation of activities
	2	Symptomatic: Limitation of activities
Hospitalized: Mild disease	3	Hospitalized; no oxygen therapy
	4	Hospitalized; oxygen by mask or nasal prongs
Hospitalized: Severe disease	5	Non-invasive ventilation or high-flow oxygen
	6	Intubation & Mechanical ventilation
	7	Ventilation and additional organ support – pressors, RRT, ECMO
Death	8	Death

Appendix 3: Therapeutic-dose Anticoagulation (Arm A)

Based on DSMB review and NHLBI Determination, as of Dec 19, 2020 patients who require ICU level of care (severe illness) at screening/potential enrollment are NOT eligible for Arm A.

Any of the following strategies are recommended for therapeutic-dose anticoagulation:

A3.1 Therapeutic Dose Anticoagulation**

CrCl	BMI	Enoxaparin	Dalteparin	Tinzaparin	Heparin
≥30	<40	1 mg/kg SC q12h<br>OR<br>1.5 mg/kg SC q24h	200 units/kg SC q24h<br>OR<br>100 units/kg SC q12h	175 units/kg SC q24h	IV bolus, with continuous infusion to titrate to anti-Xa 0.3-0.7 IU/mL or corresponding aPTT values*
	≥40	1 mg/kg SC q12h	100 units/kg SC q12h		
<30	<40	Heparin IV bolus, with continuous infusion to titrate to anti-Xa 0.3-0.7 IU/mL or corresponding aPTT values*			
	≥40				

* Initial bolus dose determined by sites, encouraging use of dosing algorithm designed for treatment of VTE. UFH anti-Xa titration is preferred over aPTT if available because achieving a therapeutic aPTT may be challenging in patients with a pro-inflammatory state such as COVID-19.
Note: Tinzaparin commonly used in Canada
Note: Fondaparinux not advised in this setting due to its long half life
**These drugs are considered standard of care as an anticoagulant[1]. Different drugs are used in different regions, countries, and hospital formularies. In this pragmatic trial of antithrombotic therapy in COVID-19, sites will use the anticoagulant that they typically use in the hospital setting.

It is recommended that participants be given therapeutic-dose parenteral anticoagulation daily for at least 14 days or until hospital discharge, whichever comes first. Treatment may continue beyond 14 days at the discretion of the most responsible physician. At the time of treatment discontinuation, standard of care antithrombotic prophylaxis should be administered.

If aspirin is to be prescribed, the maximum dose permitted is 162 mg per day. If a P2Y12 was prescribed before randomization, it is stopped at the time of randomization. Note that requirement for P2Y12 inhibitor or for a dose of aspirin greater than 162 mg per day is an exclusion criterion. However, a P2Y12 inhibitor may be used if a clinical indication develops eg, coronary artery stenting. This is not a protocol deviation.

If there is a change in status such that the participant becomes severely ill, continue assigned treatment unless:
a) There are contraindications
b) Clinical judgment leads to a change in dose

Follow up continues through 90 days regardless of the change in status.

A3.2 Discontinuation of study intervention:

Patients randomized based on suspicion of COVID-19 whose tests do not confirm SARS CoV2 infection should not continue to receive study assigned therapeutic dose anticoagulation.

Anticoagulation should be discontinued if there is clinical bleeding or other complications sufficient to warrant cessation in the opinion of the treating clinician. Major bleeding, including death due to bleeding, is an SAE. Assigned treatment may be resumed if deemed appropriate by the treating clinician.

Occurrence of HIT must result in the cessation of UFH or LMWH without recommencement regardless of treatment assignment. The use of an acceptable alternative agent is required in this instance as clinically indicated. Occurrence of HIT is an SAE.

Study interventions can be discontinued at any time by the treating clinician if doing so is regarded as being in the best interests of the patient. Temporary cessation – for the shortest period of time possible, but not longer than 24 hours – such as to allow surgical or other procedures is not a protocol deviation.

Temporary or permanent cessation of study intervention for bleeding is not a protocol deviation.

A3.3 Study Schedule

**Activity**	**Screening/ Enrollment**	**Hospital Duration**	**28 days and/or hospital discharge+**	**90 days post randomization**
**Eligibility**				
Consent	X			
Demographic and Medical History	X			
Assessment of Inclusion/Exclusion criteria	X			
Self-reported race/ethnicity and sex	X			
Pregnancy Test, for women of childbearing potential	X			
**Study Drug Administration**				
Randomization	X			
Study treatment	X	X¥		
**Study Procedures**				
Height	X			
Weight	X			
Vital signs	X	X		
Concomitant medications	X	X		
WHO ordinal assessment	X	X	X	X
Quality of Life and Functional Status#	X			X

Outcomes assessment		X	X	X[++]
**SOC Laboratory Assessments**				
Chemistry panel	X	X	X^	
CBC with platelet count	X	X	X^	
Blood Group*	X			
PT, PTT if known	X	X		
Anticoagulation Monitoring (e.g., PTT/ Antifactor Xa level)**	X	X (site-specific)		
D-dimer***	X	X	X^	
Troponin****	X	X	X^	
Coagulation and inflammatory markers*****	X	X	X^	
Optional Biorepository	X	X		

*Blood group taken from hospital record or self report if that is not available.
** Frequency and mode (Anti-factor Xa/aPTT) of testing will be based on site routine. Anti-factor Xa monitoring is preferred over PTT
***Baseline D-dimer is strongly recommended (sample should be obtained prior to randomization, and results may need to be available at the time of randomization if the D-dimer value is needed to assess arm-specific eligibility). All values collected should be recorded
****Strongly recommended as part of routine care
***** Optional, listed in case report form
[+]Assessments indicated in the table above will be ascertained at discharge, or at 28 days, whichever comes first. Participants must be followed for vital status until discharged from the hospital or another care facility (if transferred on organ support) up to 90 days. To maximize retention, participants will be contacted intermittently (e.g. at one and two months post-discharge)
[¥]or 14 days, whichever is earlier
[#]Participants may be assessed for functional status and quality of life that reflects baseline status pre-COVID illness and functional status and quality of life at 90 days, when contacted to ascertain vital status. (Instruments detailed in the manual of operations).
[++]Participants may be contacted to ascertain vital status.
^ May be collected at hospital discharge and at 28 days in participants who remain in hospital at that time

A3.4 Potential Risks & Benefits

A3.4.1 Known Potential Risks

Participants are monitored as per standard of care to minimize the risk of bleeding or developing clots. The therapeutic dose anticoagulation group will receive potent anticoagulation and thus may be at higher risk of bleeding.

A3.4.2 Known Potential Benefits

A study from patients with COVID-19 hospitalized in China found that patients with elevated D-dimer had the benefit of prophylactic dose anticoagulation versus no anticoagulation. Thus, there is a direct benefit of decreased clotting events in patients treated with any anticoagulation. The therapeutic dose anticoagulation arm demonstrated superiority to the prophylactic-dose anticoagulation arm in moderate patients in version 1.0 of this study protocol. All participants will be closely monitored by the study team and any changes will be discussed with the treating physicians and/or clinical team. There is a potential direct benefit of identifying clots or bleeding more rapidly

based on this monitoring. This trial will contribute to the body of generalizable knowledge about the best antithrombotic strategy to use to minimize the risk of clotting in patients with COVID-19.

A3.5 Study Enrollment

A3.5.1 Inclusion Criteria

Same as the Master Protocol.
Based on the pathophysiology of COVID-19 associated thrombosis, we seek to primarily enroll patients with an elevated D-dimer. It is strongly recommended to enroll patients with a documented D-dimer above the upper limit of normal for the institutional range.

A3.5.2 Exclusion Criteria

In addition to the exclusion criteria noted in the master protocol, arm-specific exclusion criteria are as follows:

- Severe illness - requires ICU level of care at the time of randomization (receiving HFNO, NIV, IV, vasopressors or inotropes, or ECMO)
- Contraindication to anticoagulation, including but not limited to:
    - known bleeding within the last 30 days requiring emergency room presentation or hospitalization
    - known hypersensitivity to any of the study agents
    - known history of an inherited or active acquired bleeding disorder
    - known history of heparin induced thrombocytopenia
    - recent ischemic stroke
    - history of intracranial hemorrhage at any time
- Platelet count < 50x 10^9/L
- Hemoglobin < 8 g/dL
- Requirement for ASA >162 mg per day that it cannot be stopped safely
- Requirement for P2Y12 inhibitor that cannot be stopped safely

A3.6 Event Adjudication

A subset of thrombotic events will be centrally adjudicated, with the proportion adjusted as needed based on agreement between the site and the event committee.

A3.7 Safety Analyses

The safety event of importance for the therapeutic dose anticoagulation is major bleeding. The rates of ISTH major bleeding, ICH and fatal bleeds, and mortality will be monitored. For ISTH major bleeding, ICH and fatal bleeds, and all-cause mortality the DSMB will review the number of events, the event rates, and the posterior mean and 95% credible intervals for the event rates, difference between arms, and odds-ratios between arms will be summarized.

A3.8 Statistical Analyses

The therapeutic dose anticoagulation arm demonstrated superiority to the prophylactic-dose anticoagulation arm in moderate patients. This arm is continuing in the trial and will serve as a control arm for efficacy on the primary analysis and the secondary endpoints and safety analyses for additional arms.

The primary Bayesian statistical model (see Appendix 1.3) will be used for modeling this arm in comparing to additional arms. Appendix 1.3 presents the interim analysis schedule and adaptive decision rules.

A3.9 References

1. Garcia D, Baglin T, Weitz J, et al. Parenteral Anticoagulants: Antithrombotic Therapy and Prevention of Thrombosis, 9th ed: American College of Chest Physicians Evidence-Based Clinical Practice Guidelines. Chest 2012 Feb;141(2 Suppl):e24S–e43S.

Appendix 4: Prophylactic Dose Anticoagulation (Arm B)

Based on DSMB review and NHLBI Determination, as of Jan 21, 2021 patients who are non-ICU level of care at screening/potential enrollment (moderate illness) are NOT eligible for Arm B.

Any of the following strategies are recommended for prophylactic dose anticoagulation:

A4.1 Prophylactic Dose Anticoagulation*

CrCl	BMI	Enoxaparin	Dalteparin	Tinzaparin	Fondaparinux	Heparin
≥30	<40	40 mg SC q24h	5000 units SC q24h	4500 units SC q24h	2.5 mg SC q24h	5000 units SC q8-12h
	≥40	40 mg SC q12h	5000 units SC q12h	9000 units SC q24h	NA	7500 units SC q8h
<30	<40	Heparin 5000 units SC q8-12h				
	≥40	Heparin 7500 units SC q8h				

*All drugs are considered standard of care as an anticoagulant[1,2]. Different drugs are used in different regions, countries, and hospital formularies. As a pragmatic trial of antithrombotic therapy in COVID-19, sites will use the anticoagulant that they typically use in the hospital setting.

It is recommended that participants be given prophylactic-dose parenteral anticoagulation daily for at least 14 days or until hospital discharge, whichever comes first. Treatment may continue beyond 14 days at the discretion of the most responsible physician.

Full therapeutic dose anticoagulation (therapeutic dose UFH or LMWH) should be used for clinical indications including a thrombotic event, atrial fibrillation, acute coronary syndrome.

If aspirin is to be prescribed, the maximum dose permitted is 162 mg per day. If a P2Y12 was prescribed before randomization, it is stopped at the time of randomization. Note that requirement for P2Y12 inhibitor or for a dose of aspirin greater than 162 mg per day is an exclusion criterion. However, a P2Y12 inhibitor may be used if a clinical indication develops eg, coronary artery stenting. This is not a protocol deviation.

A4.2 Discontinuation of study intervention

Anticoagulation should be discontinued if there is clinical bleeding or another complication sufficient to warrant cessation in the opinion of the treating clinician. Major bleeding, including death due to bleeding, is an SAE. Assigned treatment may be resumed if deemed appropriate by the treating clinician.

Occurrence of HIT must result in the cessation of UFH or LMWH without recommencement regardless of treatment assignment. Use of an acceptable alternative agent is required in this instance as clinically indicated. Occurrence of HIT is an SAE.

Study interventions can be discontinued at any time by the treating clinician if doing so is regarded as being in the best interests of the patient. Temporary cessation – for the shortest period of time

possible, but not longer than 24 hours – such as to allow surgical or other procedures is not a protocol deviation.

Temporary or permanent cessation of study intervention for bleeding is not a protocol deviation.

A4.3 Study Schedule

Activity	Screening/ Enrollment	Hospital Duration	28 days and/or hospital discharge[+]	90 days post randomization[++]
**Eligibility**				
Consent	X			
Demographic and Medical History	X			
Assessment of Inclusion/Exclusion criteria	X			
Self-reported race/ethnicity and sex	X			
Pregnancy Test, for women of childbearing potential	X			
**Study Drug Administration**				
Randomization	X			
Study treatment	X	X[¥]		
**Study Procedures**				
Height	X			
Weight	X			
Vital signs	X	X		
Concomitant medications	X	X		
WHO ordinal assessment	X	X	X[^]	X
Quality of Life and Functional Status[#]	X			X
Outcomes assessment		X	X	X[++]
**SOC Laboratory Assessments**				
Chemistry panel	X	X	X[^]	
CBC with platelet count	X	X	X[^]	
Blood Group*	X			
PT, PTT if known	X	X		
Anticoagulation Monitoring (ex, PTT/ Antifactor Xa level)**	X	X (site-specific)		
D-dimer***	X	X	X[^]	
Troponin****	X	X	X[^]	
Coagulation and inflammatory markers*****	X	X	X[^]	
Optional Biorepository	X	X		

*Blood group taken from hospital record or self report if that is not available.
** Frequency and mode (Anti-factor Xa/PTT) of testing will be based on site routine. Anti-factor Xa monitoring is preferred over PTT
All values collected should be recorded.
****Strongly recommended as part of routine care, all values collected should be recorded
*****Optional, listed in case report form
+Assessments indicated in the table above will be ascertained at discharge, or at 28 days, whichever comes first. Participants must be followed for vital status until discharged from the hospital or another care facility (if transferred on organ support) up to 90 days. To maximize retention, participants will be contacted intermittently (e.g. at one and two months post-discharge)
¥or 14 days, whichever is earlier
#Participants may be assessed for functional status and quality of life that reflects baseline status pre-COVID illness and functional status and quality of life at 90 days, when contacted to ascertain vital status. (Instruments detailed in the manual of operations).
++Participants may be contacted to ascertain vital status.^ May be collected at hospital discharge and at 28 days in participants who remain in hospital at that time

A4.4 Potential Risks & Benefits

A4.4.1 Known Potential Risks

Participants are monitored as per standard of care to minimize the risk of bleeding or developing clots.

A4.4.2 Known Potential Benefits

A study from patients with COVID-19 hospitalized in China found that patients with elevated D-dimer had a benefit of prophylactic dose anticoagulation versus no anticoagulation. Thus, there is a direct benefit of decreased clotting events in patients treated with any anticoagulation. All participants will be closely monitored by the study team and any changes will be discussed with the treating physicians and/or clinical team. There is a potential direct benefit of identifying clots or bleeding more rapidly based on this monitoring. This trial will contribute to the body of generalizable knowledge about the best antithrombotic strategy to use to minimize the risk of clotting in patients with COVID-19.

A4.5 Study Enrollment

A4.5.1 Inclusion Criteria

Same as the Master Protocol.

A4.5.2 Exclusion Criteria

In addition to the exclusion criteria noted in the master protocol, arm-specific exclusion criteria are as follows:

- Moderate illness severity – non-ICU level of care at the time of randomization (not receiving HFNO, NIV, IV, vasopressors or inotropes, or ECMO)
- Contraindication to anticoagulation, including but not limited to
  - known bleeding within the last 30 days requiring emergency room presentation or hospitalization
  - known hypersensitivity to any of the study agents
  - known history of an inherited or active acquired bleeding disorder
  - known history of heparin induced thrombocytopenia

    - recent ischemic stroke
- Indication for therapeutic anticoagulation in the case that it cannot be stopped safely
- Platelet count < 50x 10^9/L
- Hemoglobin < 8 g/dL
- Requirement for ASA >162mg per day that cannot be stopped safely
- Requirement for P2Y12 inhibitor that cannot be stopped safely

A4.6 Event Adjudication
A subset of thrombotic events will be centrally adjudicated, with the proportion adjusted as needed based on agreement between the site and the event committee.

A4.7 Safety Analyses
The safety event of importance for the prophylactic dose anticoagulation is serious thrombotic events. The risk is that with a sub therapeutic dose there may be elevated thrombotic events. The rates of serious thrombotic events and mortality will be monitored. For serious thrombotic events the DSMB will review the number of events, the event rates, and the posterior mean and 95% credible intervals for the event rates, difference between arms, and odds-ratios between arms will be summarized.

A4.8 Statistical Analyses
The comparison of therapeutic to prophylactic dose anticoagulation was stopped due to futility and concern for harm in severely ill patients. This arm is continuing in the trial and will serve as a control arm for efficacy on the primary analysis and the secondary endpoints and safety analyses for additional arms.

The primary Bayesian statistical model (see Appendix 1.3) will be used for modeling this arm in comparing to other arms. Appendix 1.3 presents the interim analysis schedule and adaptive decision rules.

A4.9 References
1. Gould MK, Garcia DA, Wren SM, et al. Prevention of VTE in nonorthopedic surgical patients: antithrombotic therapy and prevention of thrombosis, 9th ed: American College of Chest Physicians evidence-based clinical practice guidelines. Chest. 2012;141(2 suppl):e227S-277S.

2. Garcia D, Baglin T, Weitz J, et al. Parenteral Anticoagulants: Antithrombotic Therapy and Prevention of Thrombosis, 9th ed: American College of Chest Physicians Evidence-Based Clinical Practice Guidelines. Chest 2012 Feb;141(2 Suppl):e24S–e43S.

Appendix 5: ACTIV-4 Blood Sampling – proposed samples and times for sites participating in mechanistic studies and biorepository

The goal of the Mechanistic Studies Center and the Biorepository/Central Lab is to add significant value to the clinical trials by collecting high-quality blood samples for studies aimed at elucidating underlying disease mechanisms and insights into how the therapy modifies these underlying disease processes. A goal is to identify biomarkers that can identify pathological mechanisms, predict outcomes, direct therapy, and/or identify higher-risk patient subpopulations.

A5.1 Inpatient sampling

Blood collection times for inpatients:

- Days 0 (time of enrollment), 3, 7 and 14. Samples should be obtained within 24 hours after the assigned time point (example: the day 3 sample should be obtained within 72-96 hours after randomization)
- Samples should be coordinated with clinical lab blood draws when possible.

Standard samples to be collected & volumes at each time point:

- Citrate plasma
  - Two 4.5 mL Citrate tubes (BD # 369714)
- EDTA plasma
  - One 10 mL EDTA tube (BD# 366643)
- Serum
  - One 5.0 mL Serum tube (BD # 367814)

Note 1: *We anticipate that some sites may not be able to collect & process all the samples and time points listed above. We plan to work with those sites to identify more limited time points and/or discard samples that could be collected, processed and sent to the biorepository.*

Note 2: *We anticipate that some high-functioning sites may, in addition to the sample collections noted above, also participate in enhanced collections & studies, which may include:*

- Additional blood collection tubes such as:
  - HTI SCAT-144 plasma
  - Paxgene RNA whole blood
  - Cell Prep Tube (CPT)
- Whole blood assays:
  - Viscoelastic assays (thromboelastography or thromboelastometry)
  - Platelet aggregometry
  - Whole blood genomics

A5.2 Sample processing

A detailed Manual of Operations (MOP) will provide instructions to clinical lab and research personnel regarding sample processing including centrifugation, processing, freezing, storing, & shipping samples. Also, the following will be provided: training materials; sample processing kits with prelabeled transport and/or storage vials; sample tracking software; shipping materials.

***A5.3* Biorepository/Central Lab**

The Biorepository will archive biosamples from the clinical sites, and distribute them to the labs doing ACTIV-4 approved mechanistic studies and other research. If ACTIV-4 biosamples cannot be shipped to the Biorepository for some reason, the information will be captured and used to form a "Virtual Biorepository", so that those samples can contribute to the mechanistic studies as well.

Appendix 6: Additional data inclusion from other trials merged under ACTIV-4 platform

There are several clinical trials that have been testing safety and efficacy of Arm A and B regimens. Data collected in these trials will be included in the data analysis under this protocol provided that the subjects consented for the data to be shared or a waiver of consent and authorization had been granted by the reviewing IRB. The data will be labeled with subject ID and only include dates which are necessary to assess safety and efficacy endpoint events. All other private health information (PHI) will be removed. The data will be stored at the study coordinating center, University of Pittsburgh, in HIPAA compliant electronic system and only coordinating center staff will have access to the data. The statistical analysis plan will account for this additional data.

Appendix 7: Background and Rationale for Arm C: Prophylactic-dose anticoagulation, plus P2Y12 inhibitor for ICU Level of Care (Severe) Cohort

A7.1 Background and Rationale for Arm C

Analysis of therapeutic vs prophylactic anticoagulation in severely ill patients demonstrated that therapeutic anticoagulation was not superior, and there was a trend toward harm. Therefore prophylactic dose anticoagulation is considered standard of care for severely ill patients at the time of this writing. The number of days with organ support or death over the first 21 days of the index hospitalization remained high despite treatment with prophylactic dose anticoagulants, and bleeding risk was < 2%. Therefore, additional antithrombotic strategies should be tested.

Autopsy and clinical data highlight the potential role of platelets and their precursors in the pathogenesis of COVID-19.[1-3] Platelets are shed into circulation by megakaryocytes, and during this process, megakaryocytes distribute their transcriptome into platelets. Once in the circulation, platelets can respond to local and systemic conditions and induce monocyte, macrophage and endothelial cell activation.[4-6] Prior to COVID-19, it was described that platelet-viral interactions alter the platelet transcriptome and induce a proinflammatory immune mediated platelet phenotype.[7] Consistently, platelets isolated from COVID-19 patients are hyperreactive and have an altered transcriptomic signature compared to disease-free controls.[3] Biomarkers of platelet activity are elevated in COVID-19 and are associated with thrombosis and all-cause mortality even after multivariable adjustment. These data suggest that platelets are activated in COVID-19 and represent a therapeutic target for improved clinical outcomes.

A7.2. Eligibility Criteria for Arm C

A7.2.1 Inclusion Criteria for Arm C

Same as the Master Protocol.

A7.2.2 Exclusion Criteria for Arm C

In addition to the exclusion criteria noted in the master protocol, arm-specific exclusion criteria are as follows:

- Moderate illness severity – non-ICU level of care at the time of randomization (not receiving HFNO, NIV, IV, vasopressors or inotropes, or ECMO)
- Contraindication to anticoagulation (heparin or LMWH) or P2Y12 inhibitor, including but not limited to
  - known bleeding within the last 30 days requiring emergency room presentation or hospitalization
  - known hypersensitivity to any of the study agents
  - known history of an inherited or active acquired bleeding disorder
  - known history of heparin induced thrombocytopenia
  - recent ischemic stroke
  - history of intracranial hemorrhage at any time
- Indication for therapeutic anticoagulation in the case that it cannot be stopped safely
- Platelet count < 50 x 10^9/L
- Hemoglobin < 8 g/dL
- Requirement for ASA >162mg per day that it cannot be stopped safely
- Requirement for P2Y12 inhibitor that cannot be stopped safely

A7.3 Study Agents

Arm C consists of the combination of prophylactic-dose anticoagulation, plus an antiplatelet agent in the P2Y12 inhibitor family.

A7.3.1. Prophylactic Dose Anticoagulation*

Any of the following strategies are recommended for prophylactic dose anticoagulation:

CrCl	BMI	Enoxaparin	Dalteparin	Tinzaparin	Fondaparinux	Heparin
≥30	<40	40 mg SC q24h	5000 units SC q24h	4500 units SC q24h	2.5 mg SC q24h	5000 units SC q8-12h
	≥40	40 mg SC q12h	5000 units SC q12h	9000 units SC q24h	NA	7500 units SC q8h
<30	<40	Heparin 5000 units SC q8-12h				
	≥40	Heparin 7500 units SC q8h				

*All drugs are considered standard of care as an anticoagulant.[8,9] Different drugs are used in different regions, countries, and hospital formularies. As a pragmatic trial of antithrombotic therapy in COVID-19, sites will use the anticoagulant that they typically use in the hospital setting.

It is recommended that participants be given prophylactic-dose parenteral anticoagulation daily for at least 14 days or until hospital discharge, whichever comes first. Treatment may continue beyond 14 days at the discretion of the most responsible physician.

Full therapeutic dose anticoagulation (therapeutic dose UFH or LMWH) should be used for clinical indications including a thrombotic event, atrial fibrillation, acute coronary syndrome.

A7.3.2. P2Y12 Inhibitor

The table shows the preferred dosing for P2Y12 inhibitor treatment. Ticagrelor is the preferred P2Y12 inhibitor, but any of the following strategies are acceptable.

Age	Weight	Ticagrelor#	Prasugrel*	Clopidogrel
<75 years	<60 kg	no load; 60 mg BID	no load, 5 mg daily	300 mg load**, then 75 mg daily
	≥60 kg		30 mg load**, 10 mg daily	
≥75 years	<60 kg	no load; 60 mg BID	not recommended	300 mg load**, then 75 mg daily
	≥60 kg		no load, 5 mg daily	

The preferred P2Y12 inhibitor is ticagrelor, which has a rapid onset of action without the need for a loading dose, unless there is a concern about drug-drug interactions (see Manual of Operations). The 60 mg twice daily dose is recommended ; *If ticagrelor 60 mg is not available, 90 mg dose, may be used.* If ticagrelor is not available or is not preferred locally, prasugrel or clopidogrel may be used, preferably with a loading dose, taking into account relevant drug-drug interactions (see Manual of Operations).

# if a participant will be continued on aspirin in addition the assigned P2Y12 inhibitor, aspirin dose must be ≤100 mg when administered with ticagrelor

* Prasugrel is NOT permitted in anyone with a prior stroke or TIA

**The loading dose is preferred because the average time to therapeutic effect with clopidogrel is 5 days without a loading dose, and for prasugrel is 3 days without a loading dose. A loading dose is not required.

A7.3.3. Participants previously taking aspirin before randomization

Participants taking aspirin before randomization are permitted to continue or stop aspirin therapy at the discretion of the treating physician. If a patient is randomized to Arm C and chronic ASA is continued per MD judgment, the recommended dose is 80-100 mg daily; the dose MUST be ≤100 mg daily when administered with ticagrelor.

A7.4 Duration of treatment

It is recommended that participants be given prophylactic-dose parenteral anticoagulation and a P2Y12 inhibitor daily for at least 14 days or until hospital discharge, whichever comes first. Treatment may continue beyond 14 days at the discretion of the most responsible physician. At the time of treatment discontinuation, standard of care antithrombotic prophylaxis should be administered.

A7.5 Discontinuation of study intervention

Anticoagulation and/or P2Y12 inhibitor should be discontinued if there is clinical bleeding or another complication sufficient to warrant cessation in the opinion of the treating clinician. Major bleeding, including death due to bleeding, is an SAE. Assigned treatment may be resumed if deemed appropriate by the treating clinician.

Occurrence of HIT must result in the cessation of UFH or LMWH without recommencement regardless of treatment assignment. Use of an acceptable alternative agent is required in this instance as clinically indicated. Occurrence of HIT is an SAE. If HIT occurs, the P2Y12 inhibitor may be continued, at the discretion of the treating physician, taking into account the platelet count.

Study interventions can be discontinued at any time by the treating clinician if doing so is regarded as being in the best interests of the patient. Temporary cessation – for the shortest period of time possible – such as to allow surgical or other procedures is not a protocol deviation.

Temporary or permanent cessation of study intervention for bleeding is not a protocol deviation.

A7.6 Study Schedule

Activity	Screening/ Enrollment	Hospital Duration	28 days and/or hospital discharge+	90 days post randomization++
**Eligibility**				
Consent	X			
Demographic and Medical History	X			
Assessment of Inclusion/Exclusion criteria	X			
Self-reported race/ethnicity and sex	X			
Pregnancy Test, for women of childbearing potential	X			
**Study Drug Administration**				
Randomization	X			
Study treatment	X	X¥		
**Study Procedures**				
Height	X			
Weight	X			
Vital signs	X	X		
Concomitant medications	X	X		
WHO ordinal assessment	X	X	X^	X
Quality of Life and Functional Status#	X			X
Outcomes assessment		X	X	X++
**SOC Laboratory Assessments**				
Chemistry panel	X	X	X^	

CBC with platelet count	X	X	X^	
Blood Group*	X			
PT, PTT if known	X	X		
Anticoagulation Monitoring (ex, PTT/ Antifactor Xa level)**	X	X (site-specific)		
D-dimer***	X	X	X^	
Troponin****	X	X	X^	
Coagulation and inflammatory markers*****	X	X	X^	
Optional Biorepository	X	X		

*Blood group taken from hospital record or self report if that is not available.
** Frequency and mode (Anti-factor Xa/PTT) of testing will be based on site routine. Anti-factor Xa monitoring is preferred over PTT
*** Baseline D-dimer is recommended. All values collected should be recorded.
****Strongly recommended as part of routine care, all values collected should be recorded
*****Optional, listed in case report form
[¥]or 14 days, whichever is earlier
[#]Participants may be assessed for functional status and quality of life that reflects baseline status pre-COVID illness and functional status and quality of life at 90 days, when contacted to ascertain vital status. (Instruments detailed in the manual of operations).
[+]Assessments indicated in the table above will be ascertained at discharge, or at 28 days, whichever comes first. Participants must be followed for vital status until discharged from the hospital or another care facility (if transferred on organ support) up to 90 days. To maximize retention, participants will be contacted intermittently (e.g. at one and two months post-discharge)
[++]Participants may be contacted to ascertain vital status.
^ May be collected at hospital discharge and at 28 days in participants who remain in hospital at that time

A7.7 Potential Risks & Benefits

A7.7.1 Known Potential Risks

Participants are monitored as per standard of care to minimize the risk of bleeding or developing clots. The prophylactic dose anticoagulation plus P2Y12 inhibitor group will receive both anticoagulation and antiplatelet therapy and thus may be at higher risk of bleeding.

A7.7.2 Known Potential Benefits

Accruing data suggest that platelets are hyperactive in the setting of COVID-19. The platelet transcriptome isolated from hospitalized patients with COVID-19 is more pro-inflammatory than the platelet transcriptome from matched controls without COVID-19. Additionally, biomarkers of platelet activity are correlated with incident thrombosis and all-cause mortality. This arm seeks to test the hypothesis that there is a benefit of antiplatelet therapy in addition to prophylactic dose anticoagulation for decreasing adverse events, including macro and micro-thrombosis. This potential benefit is hypothesized to offset an increase in bleeding risk. All participants will be closely monitored by the study team and any changes in antiplatelet therapy will be discussed with the treating physicians and/or clinical team. There is a potential direct benefit of identifying thrombus or bleeding more rapidly based on close study monitoring. This trial will contribute to the body of generalizable knowledge about the antiplatelet strategy to minimize the risk of thrombus and adverse events in patients with COVID-19.

A7.8 Event Adjudication

A subset of thrombotic events will be centrally adjudicated, with the proportion adjusted as needed based on agreement between the site and the event committee.

A7.8 Safety Analyses

The safety events of importance for the prophylactic dose anticoagulation plus P2Y12 inhibitor are serious thrombotic events and bleeding. The rates of serious thrombotic events and mortality will be monitored. The rates of serious thrombotic events will be compared to the prophylactic dose anticoagulation arm, as well as to any additional arms added to the platform trial subsequently. For serious thrombotic events the DSMB will review the number of events, the event rates, and the posterior mean and 95% credible intervals for the event rates, difference between arms, and odds-ratios between arms will be summarized.

Major bleeding is a safety event of importance for this arm. The rates of ISTH major bleeding, ICH and fatal bleeds, and mortality will be monitored. The rates of bleeding will be compared to the control arm (prophylactic dose anticoagulation, no P2Y12 inhibitor) as well as to additional arms. For ISTH major bleeding, ICH and fatal bleeds, and all-cause mortality the DSMB will review the number of events, the event rates, and the posterior mean and 95% credible intervals for the event rates, difference between arms, and odds-ratios between arms will be summarized.

A7.9 Statistical Analyses

The prophylactic dose anticoagulation arm was demonstrated as a therapeutic advantage compared to therapeutic anticoagulation in severe patients. This arm is testing the potential advantage of adding a P2Y12 to the prophylactic dose anticoagulation. This arm will be compared to the prophylactic dose anticoagulation arm for efficacy on the primary analysis and the secondary endpoints and safety analyses for additional arms.

The primary Bayesian statistical model (see Appendix 1.3) will be used for modeling this arm in comparing to other arms. Appendix 1.3 presents the interim analysis schedule and adaptive decision rules.

A7.13 References

1. Rapkiewicz AV, Mai X., Carsons SE, et al. Megakaryocytes and platelet-fibrin thrombi characterize multi-organ thrombosis at autopsy in COVID-19: A case series. *EClinical Medicine.* 2020; Jun 25;24:100434.
2. Barrett TJ, Lee A, Xia Y, et al. Biomarkers of Platelet Activity and Vascular Health Associate with Thrombosis and Mortality in Patients with COVID-19. *Circ Res.* 2020.
3. Manne BK, Denorme F, Middleton EA, et al. Platelet Gene Expression and Function in COVID-19 Patients. *Blood.* 2020.
4. Nhek S, Clancy R, Lee KA, et al. Activated Platelets Induce Endothelial Cell Activation via an Interleukin-1beta Pathway in Systemic Lupus Erythematosus. *Arterioscler Thromb Vasc Biol.* 2017;37(4):707-716.
5. Campbell RA, Schwertz H, Hottz ED, et al. Human megakaryocytes possess intrinsic antiviral immunity through regulated induction of IFITM3. *Blood.* 2019;133(19):2013-2026.
6. Barrett TJ, Schlegel M, Zhou F, et al. Platelet regulation of myeloid suppressor of cytokine signaling 3 accelerates atherosclerosis. *Sci Transl Med.* 2019;11(517).
7. Assinger A. Platelets and infection - an emerging role of platelets in viral infection. *Front Immunol.* 2014;5:649.

8. Gould MK, Garcia DA, Wren SM, et al. Prevention of VTE in nonorthopedic surgical patients: antithrombotic therapy and prevention of thrombosis, 9th ed: American College of Chest Physicians evidence-based clinical practice guidelines. Chest. 2012;141(2 suppl):e227S-277S.
9. Garcia D, Baglin T, Weitz J, et al. Parenteral Anticoagulants: Antithrombotic Therapy and Prevention of Thrombosis, 9th ed: American College of Chest Physicians Evidence-Based Clinical Practice Guidelines. Chest 2012 Feb;141

Appendix 8: Therapeutic-dose Anticoagulation, Plus P2Y12 Inhibitor (Arm D) for non-ICU Level of Care (Moderate) Cohort

Based on DSMB review and NHLBI Determination, as of Dec 19, 2020 patients who require ICU level of care at screening/potential enrollment are NOT eligible for Arm D.

Arm D consists of the combination of therapeutic-dose anticoagulation, plus an antiplatelet agent in the P2Y12 inhibitor family.

A8.1 Background and Rationale for Arm D

Analysis of therapeutic vs prophylactic anticoagulation in moderately ill patients demonstrated that therapeutic anticoagulation was superior. Therefore therapeutic dose anticoagulation is considered standard of care for moderately ill patients at the time of this writing. The number of days with organ support or death over the first 21 days of the index hospitalization, remained high despite treatment with therapeutic dose anticoagulants, particularly in certain subsets, and bleeding risk was < 2%. Therefore, additional antithrombotic strategies should be tested.

Autopsy and clinical data highlight the potential role of platelets and their precursors in the pathogenesis of COVID-19.[1-3] Platelets are shed into circulation by megakaryocytes, and during this process, megakaryocytes distribute their transcriptome into platelets. Once in the circulation, platelets can respond to local and systemic conditions and induce monocyte, macrophage and endothelial cell activation.[4-6] Prior to COVID-19, it was described that platelet-viral interactions alter the platelet transcriptome and induce a proinflammatory immune mediated platelet phenotype.[7] Consistently, platelets isolated from COVID-19 patients are hyperreactive and have an altered transcriptomic signature compared to disease-free controls.[3] Biomarkers of platelet activity elevated in COVID-19 and are associated with thrombosis and all-cause mortality even after multivariable adjustment. These data suggest that platelets are activated in COVID-19 and represent a therapeutic target for improved clinical outcomes.

A8.2 Arm D Eligibility

A8.2.1 Inclusion Criteria

In addition to the inclusion criteria noted in the master protocol:

D-dimer must be $\geq$ 2-fold elevated above the upper limit of normal.
If D-dimer is < 2-fold elevated or is missing at baseline the following criteria must be met:

- Age $\geq$60 OR
- If age < 60, 1 or more of the following criteria must be met:
  - Higher O2 requirement (e.g., >2L)
  - History of a comorbid condition:
    - Diabetes mellitus
    - Hypertension
    - Chronic kidney disease (eGFR < 60 mg/dL)
    - Cardiovascular disease e.g., prior MI, known coronary artery disease, heart failure, ejection fraction < 50%
    - BMI ≥ 35 kg/m^2

A8.2.2 Exclusion Criteria for Arm D

Exclusion criteria for Arm D include any condition that, in the opinion of the investigator, is associated with a risk of bleeding that precludes use of therapeutic dose anticoagulation, plus P2Y12 inhibitor, including those listed below.

Exclusion Criteria
In addition to the exclusion criteria noted in the master protocol, arm-specific exclusion criteria are as follows:

- Severe illness - requires ICU level of care at the time of randomization (receiving HFNO, NIV, IV, vasopressors or inotropes, or ECMO)
- Age $\geq$85
- Contraindication to anticoagulation (heparin or LMWH) or P2Y12 inhibitor, including but not limited to
  - known bleeding within the last 30 days requiring emergency room presentation or hospitalization
  - known hypersensitivity to any of the study agents
  - known history of an inherited or active acquired bleeding disorder
  - known history of heparin induced thrombocytopenia
  - recent ischemic stroke
  - history of intracranial hemorrhage at any time
- Any condition that, in the opinion of the investigator, is associated with a risk of bleeding that precludes use of therapeutic dose anticoagulation, plus P2Y12 inhibitor. Examples include:
  - Structural cerebrovascular lesion
  - Any history of stroke
  - Recent surgery
  - Severe, uncontrolled hypertension (e.g., BP >185/100)
- Platelet count < 50 x 10^9/L
- Hemoglobin < 8 g/dL
- Indication for P2Y12 inhibitor in the case that it cannot be stopped safely
- Indication for aspirin in the case that aspirin cannot be exchanged for a P2Y12 inhibitor (aspirin must be able to be discontinued if the participant is randomized to Arm D)

A8.3 Study Agents

It is recommended that participants be given therapeutic-dose parenteral anticoagulation and a P2Y12 inhibitor daily for at least 14 days or until hospital discharge, whichever comes first. Treatment may continue beyond 14 days at the discretion of the most responsible physician. At the time of treatment discontinuation, standard of care antithrombotic prophylaxis should be administered.

If there is a change in status such that the participant becomes severely ill, continue assigned treatment unless:

a) There are contraindications
b) Clinical judgment leads to a change in dose

Follow up continues through 90 days regardless of the change in status.

A8.3.1. Therapeutic Dose Anticoagulation**

Any of the following strategies are recommended for therapeutic-dose anticoagulation:

CrCl	BMI	Enoxaparin	Dalteparin	Tinzaparin	Heparin
≥30	<40	1 mg/kg SC q12h<br>OR<br>1.5 mg/kg SC q24h	200 units/kg SC q24h<br>OR<br>100 units/kg SC q12h	175 units/kg SC q24h	IV bolus, with continuous infusion to titrate to anti-Xa 0.3-0.7 IU/mL or corresponding aPTT values*
	≥40	1 mg/kg SC q12h	100 units/kg SC q12h		
<30	<40	Heparin IV bolus, with continuous infusion to titrate to anti-Xa 0.3-0.7 IU/mL or corresponding aPTT values*			
	≥40				

* Initial bolus dose determined by sites, encouraging use of dosing algorithm designed for treatment of VTE. UFH anti-Xa titration is preferred over aPTT if available because achieving a therapeutic aPTT may be challenging in patients with a pro-inflammatory state such as COVID-19.
Note: Tinzaparin commonly used in Canada
Note: Fondaparinux not advised in this setting due to its long half life
**These drugs are considered standard of care as anticoagulants. Different drugs are used in different regions, countries, and hospital formularies. In this pragmatic trial of antithrombotic therapy in COVID-19, sites will use the anticoagulant that they typically use in the hospital setting.

A8.3.2. P2Y12 Inhibitor

The table shows the preferred dosing for P2Y12 inhibitor treatment. Ticagrelor is the preferred P2Y12 inhibitor, but any of the following strategies are acceptable.

Age	Weight	Ticagrelor	Prasugrel*	Clopidogrel
<75 years	<60 kg	no load;<br>60 mg BID[#]	no load; 5 mg daily	300 mg load**,<br>then 75 mg daily
	≥60 kg		30 mg load**; 10 mg daily	
75-85 years	<60 kg	no load;<br>60 mg BID[#]	not recommended	300 mg load**,<br>then 75 mg daily
	≥60 kg		no load; 5 mg daily	

The preferred P2Y12 inhibitor is ticagrelor, which has a rapid onset of action without the need for a loading dose, unless there is a concern about drug-drug interactions (see Manual of Operations). The 60 mg twice daily dose is recommended[#]. [#]If ticagrelor 60 mg is not available, 90 mg dose, may be used. If ticagrelor is not available or is not preferred locally, prasugrel or clopidogrel may be used, taking into account relevant drug-drug interactions (see Manual of Operations).

* Prasugrel is NOT permitted in anyone with a prior stroke or TIA

**The loading dose is preferred because the average time to therapeutic effect with clopidogrel is 5 days without a loading dose, and for prasugrel is 3 days without a loading dose. A loading dose is not required.

A8.3.3. Participants previously taking aspirin before randomization

Aspirin may not be used in arm D.

A8.4 Discontinuation of study intervention:

Patients randomized based on suspicion of COVID-19 whose tests do not confirm SARS-CoV-2 infection should not continue to receive study assigned therapeutic dose anticoagulation, plus P2Y12 inhibitor.

Anticoagulation and/or P2Y12 inhibitor should be discontinued if there is clinical bleeding or other complications sufficient to warrant cessation in the opinion of the treating clinician. Major bleeding, including death due to bleeding, is an SAE. Assigned treatment may be resumed if deemed appropriate by the treating clinician.

Occurrence of HIT must result in the cessation of UFH or LMWH without recommencement regardless of treatment assignment. The use of an acceptable alternative agent is required in this instance as clinically indicated. Occurrence of HIT is an SAE. If HIT occurs, the P2Y12 inhibitor may be continued, at the discretion of the treating physician, taking into account the platelet count.

Study interventions can be discontinued at any time by the treating clinician if doing so is regarded as being in the best interests of the patient. Temporary cessation – for the shortest period of time possible, such as to allow surgical or other procedures is not a protocol deviation.

If there is a change in status such that the participant becomes severely ill, continue assigned treatment unless:

a) There are contraindications
b) Clinical judgment leads to a change in dose

Temporary or permanent cessation of study intervention for bleeding is not a protocol deviation.

A8.5 Study Schedule

**Activity**	**Screening/ Enrollment**	**Hospital Duration**	**28 days and/or hospital discharge+**	**90 days post randomization**
**Eligibility**				
Consent	X			
Demographic and Medical History	X			
Assessment of Inclusion/Exclusion criteria	X			
Self-reported race/ethnicity and sex	X			
Pregnancy Test, for women of childbearing potential	X			
**Study Drug Administration**				
Randomization	X			

Study treatment	X	X¥		
**Study Procedures**				
Height	X			
Weight	X			
Vital signs	X	X		
Concomitant medications	X	X		
WHO ordinal assessment	X	X	X	X
Quality of Life and Functional Status assessment#	X			X
Outcomes assessment		X	X	X++
**SOC Laboratory Assessments**				
Chemistry panel	X	X	X^	
CBC with platelet count	X	X	X^	
Blood Group*	X			
PT, PTT if known	X	X		
Anticoagulation Monitoring (e.g., PTT/ Antifactor Xa level)**	X	X (site-specific)		
D-dimer***	X	X	X^	
Troponin****	X	X	X^	
Coagulation and inflammatory markers*****	X	X	X^	
Optional Biorepository	X	X		

*Blood group taken from hospital record or self report if that is not available.
** Frequency and mode (Anti-factor Xa/aPTT) of testing will be based on site routine. Anti-factor Xa monitoring is preferred over PTT
***Baseline D-dimer is strongly recommended (sample should be obtained prior to randomization and results may need to be available at the time of randomization if the d-dimer value is needed to assess arm-specific eligibility). All values collected should be recorded
****Strongly recommended as part of routine care
***** Optional, listed in case report form
¥or 14 days, whichever is earlier
#Participants may be assessed for functional status and quality of life that reflects baseline status pre-COVID illness and functional status and quality of life at 90 days, when contacted to ascertain vital status. (Instruments detailed in the manual of operations).
+Assessments indicated in the table above will be ascertained at discharge, or at 28 days, whichever comes first. Participants must be followed for vital status until discharged from the hospital or another care facility (if transferred on organ support) up to 90 days. To maximize retention, participants will be contacted intermittently (e.g. at one and two months post-discharge)
++Participants may be contacted to ascertain vital status.
^ May be collected at hospital discharge and at 28 days in participants who remain in hospital at that time

A8.6 Potential Risks & Benefits

A8.6.1 Known Potential Risks

Participants are monitored as per standard of care to minimize the risk of bleeding or developing clots. The therapeutic dose anticoagulation, plus P2Y12 inhibitor group will receive potent anticoagulation and antiplatelet therapy and thus may be at higher risk of bleeding.

A8.6.2 Known Potential Benefits

Accruing data suggest that platelets are hyperactive in the setting of COVID-19. The platelet transcriptome isolated from hospitalized patients with COVID-19 is more pro-thrombotic and more pro-inflammatory than the platelet transcriptome from matched controls without COVID-19. Additionally, biomarkers of platelet activity are correlated with incident thrombosis and all-cause mortality. This arm seeks to test the hypothesis that there is a benefit of antiplatelet therapy in addition to therapeutic dose anticoagulation for decreasing adverse events, including macro and micro-vascular thrombosis. This potential benefit is hypothesized to offset an increase in bleeding risk in this subset of the trial population at lower risk for bleeding. All participants will be closely monitored by the study team and any changes in antiplatelet therapy will be discussed with the treating physicians and/or clinical team. There is a potential direct benefit of identifying thrombus or bleeding more rapidly based on close study monitoring. This trial will contribute to the body of generalizable knowledge about the antiplatelet strategy to minimize the risk of thrombus and adverse events in patients with COVID-19.

A8.7 Event Adjudication

A subset of thrombotic events will be centrally adjudicated, with the proportion adjusted as needed based on agreement between the site and the event committee.

A8.8 Safety Analyses

The safety event of importance for the therapeutic dose anticoagulation, plus P2Y12 inhibitor arm is major bleeding. The rates of ISTH major bleeding, ICH and fatal bleeding events, and mortality will be monitored. The rates of bleeding will be compared to the other arms. For ISTH major bleeding, ICH and fatal bleeding events, and all-cause mortality the DSMB will review the number of events, the event rates, and the posterior mean and 95% credible intervals for the event rates, difference between arms, and odds-ratios between arms will be summarized.

A8.9 Statistical Analyses

The therapeutic dose anticoagulation arm was demonstrated as superior to the prophylactic dose anticoagulation in moderate patients. This arm is testing the potential advantage of adding a P2Y12 to the therapeutic dose anticoagulation. This arm will be compared to the therapeutic dose anticoagulation arm without P2Y12 (Arm A) for efficacy on the primary analysis and the secondary endpoints and safety analyses for additional arms.

The primary Bayesian statistical model (see Appendix 1.3) will be used for modeling this arm in comparing to other arms. Appendix 1.3 presents the interim analysis schedule and adaptive decision rules.

A8.10 References

1. Rapkiewicz AV, Mai X, Carsons SE, et al. Megakaryocytes and platelet-fibrin thrombi characterize multi-organ thrombosis at autopsy in COVID-19: A case series. *EClinical Medicine.* 2020; Jun 25;24:100434.
2. Barrett TJ, Lee A, Xia Y, et al. Biomarkers of Platelet Activity and Vascular Health Associate with Thrombosis and Mortality in Patients with COVID-19. *Circ Res.* 2020.

3. Manne BK, Denorme F, Middleton EA, et al. Platelet Gene Expression and Function in COVID-19 Patients. *Blood.* 2020.
4. Nhek S, Clancy R, Lee KA, et al. Activated Platelets Induce Endothelial Cell Activation via an Interleukin-1beta Pathway in Systemic Lupus Erythematosus. *Arterioscler Thromb Vasc Biol.* 2017;37(4):707-716.
5. Campbell RA, Schwertz H, Hottz ED, et al. Human megakaryocytes possess intrinsic antiviral immunity through regulated induction of IFITM3. *Blood.* 2019;133(19):2013-2026.
6. Barrett TJ, Schlegel M, Zhou F, et al. Platelet regulation of myeloid suppressor of cytokine signaling 3 accelerates atherosclerosis. *Sci Transl Med.* 2019;11(517).
7. Assinger A. Platelets and infection - an emerging role of platelets in viral infection. *Front Immunol.* 2014;5:649.

Statistical Analysis Plan
for the
Randomized Clinical Trial of
Anti-Platelet Treatment for Covid-19

Version 1.0 June 16, 2021

Table of Contents

1. COVID-19 Anti-Platelet Treatment RCT State SAP Version 3
1.1. Version History 3
2. SAP Authors 3
3. Introduction 4
3.1. Reporting Strategy 4
4. Design Considerations 4
4.1. Patient Cohorts 4
4.2. Interventions 4
4.3. Primary Endpoint 5
4.4. Adaptive Design 5
4.5. Endpoint Adjudication 6
5. Unblinding 6
6. Analysis Populations 6
7. Endpoints 7
8. Graphical Data Summaries 12
9. Descriptive Statistics 12
10. Baseline Characteristics and Co-Interventions 12
11. Adherence 13
12. Analytic Approach 13
12.1. Primary Analysis of Primary Endpoint 13
12.2. Proportional Odds Assumption 14
12.3. Analytic Approach for Secondary Dichotomous Endpoints 14
12.4. Analytic Approach for Secondary Time-To-Event Endpoints 14
12.5. Analytical Approach for Cohort Analyses 14
12.6. Markov Chain Monte Carlo (MCMC) Model Stability 15
12.7. Model Outputs 15
12.8. Exploratory Analyses 155
12.9. Handling of Missing Data 155
12.10. Definition of Times 16
12.11. Post-randomization Analyses 16
12.12. Heterogeneity of Treatment Effect 16
12.13. Adaptive Analyses 17
13. Models Reporting Outlines 19
13.1. Primary Analysis of OSFDs 19

1. COVID-19 Anti-Platelet Treatment RCT State SAP Version

1.1. Version History

Version 1: June 6, 2021

2. SAP Authors

Jeffrey Berger, MD

Scott Berry, Phd

Yu Cheng, PhD

Erinn Hade, PhD

Judith Hochman, MD

Eric Leifer, PhD

Matthew Neal, MD

Harmony Reynolds, MD

3. Introduction

This is the statistical plan for the analysis of the ACTIV4A antiplatelet treatment trial. This plan has been pre-specified by the investigators prior to unblinding of the data.

3.1. Reporting Strategy

This SAP describes the planned analyses for the study. As outlined below, two cohorts (moderate illness, severe illness) may report at different times. Multiple reports describing the results of analysis may be prepared and published to ensure expedited dissemination of the study findings. It is anticipated that for each cohort, expedited, potentially preliminary reports will describe key primary and secondary findings and limited subgroup analyses. Endpoints to be reported in these reports include:

- Organ support-free days to day 21 (primary endpoint)
  - Categorization of the primary endpoint into a three-level outcome (No organ support without death, organ support without death, death) will be analyzed to aid in the clinical clarity of the primary endpoint among severe
- In-hospital mortality (key secondary endpoint)
- Major thrombotic events or death (key secondary endpoint)
- ISTH major bleeding (key safety endpoint)

Comprehensive reports providing complete characterization of trial results may subsequently be prepared and published.

4. Design Considerations

The study was designed with a Bayesian analysis as the primary analytic method for the trial. There is one overarching Bayesian model, pre-specified in the SAP, driving all adaptations, statistical triggers, and result summaries. The decision to use a Bayesian analysis was driven in part by the uncertainty of the extent of the pandemic. The required sample size could have been small or large. Given the expected evolution of the design and uncertain sample size, a Bayesian approach is more appropriate than a traditional frequentist approach.

In this section we describe the basic defined structure of the analysis plan. This includes definitions for the patient subtypes, referred to as cohorts, the interventions, the primary endpoint, and the adaptive design.

4.1. Patient Cohorts

There are two *illness states* defined for this analysis; the severe and moderate illness cohorts. The *severe illness state* is defined as a hospitalized patient on ICU-level organ-support at time of randomization. The *moderate illness state cohort* is defined as a hospitalized patient that is not in the severe illness state.

The primary analysis for the study creates two distinct populations to analyze potential differential benefit of the interventions. In this study these distinct groups are labeled as *cohorts*.

4.2. Interventions

The two interventions for this study are labeled as *antiplatelet agent* (P2Y12 inhibitor), which plays the inferential role of the investigational treatment and no antiplatelet agent

referred to as *control*, which plays the inferential role of the control arm. These interventions are defined as:

1. Antiplatelet agent (P2Y12 inhibitor) to achieve a high level of platelet inhibition in addition to usual care anticoagulation at a level recommended for each illness state.
2. No Antiplatelet agent: usual care strategies, including anticoagulation at a level recommended for the illness state.

4.3. Primary Endpoint

The primary endpoint for the study is *organ support-free days* (OSFD). The endpoint is the number of days, out of the first 21 days after randomization, that a patient is alive and free of ICU-level organ support. For the purposes of the calculation of OSFDs, if a patient dies during their index hospitalization they will be considered the status of dead even if the death occurs after the first 21 days, through 90 days. If a patient dies during their acute index hospitalization they are coded as having –1 OSFDs, which is the worst outcome for the measure. Any patient on ICU-level organ support for all of the first 21 days that does not die, will be labeled as 0 OSFDs, the second worst outcome. The number of days is rounded to the nearest day, creating an integer valued outcome. The values of 0, 1, …, 21 refer to the number of days alive and free of organ support, with smaller values being worse outcomes. Therefore, the primary outcome OSFDs is an ordinal outcome with 23 possible outcomes for each patient, –1, 0, 1, 2, …, 21.

ICU-level of organ support is defined as high flow nasal cannula, non-invasive ventilation, invasive ventilation, extracorporeal life support, vasopressors, and/or; high flow nasal cannula is considered to be organ support when applied inspiratory flow is ≥20 L/min and $FiO_2 \geq 0.4$. Due to the varying provision of organ-support in potentially repurposed areas during the pandemic, ACTIV-4a defines any hospitalized area able to deliver the above organ-support as an ICU.

This primary endpoint is an ordinal outcome and the primary analysis model analyzes the outcome as ordinal, with a cumulative logistic proportional odds model. The measure of relative efficacy for the interventions is an odds ratio (OR) which captures the effect of having improved outcomes in OSFDs across the scale between the two interventions. The model is structured so that for P2Y12 an OR > 1 implies improved outcomes on OSFDs for the P2Y12 intervention compared to the control.

4.4. Adaptive Design

There is a prospective adaptive analysis plan for the study. The plan is to have approximately monthly analyses of the study for potential adaptive conclusions. There are two potential prospective adaptive conclusions that can be reached for the comparison of the therapeutic anticoagulation to the prophylactic anticoagulation: *superiority* and *futility*. Prospective analyses have been created where statistical thresholds for claiming superiority or futility have been defined. These statistical thresholds are referred to as *statistical triggers*.

The study defines two statistical triggers within the trial that, at any analysis of the trial, would result in a declaration of superiority or futility as trial conclusions.

The following statistical triggers were defined at the onset of the trial before unblinding:

1. *Superiority*. If P2Y12 has at least a 99% posterior probability of OR>1 for organ support-free days this would trigger a claim of superiority for P2Y12.
2. *Futility*. If P2Y12 has less than 5% posterior probability of at least a 1.20 OR compared to usual care for organ support-free days, then a claim of futility of that intervention would be declared.

For the purpose of this analysis plan, *inferiority* (harm) for P2Y12 is defined as an OR<1 with a posterior probability of >=99%.

At each adaptive and final analysis of the study each statistical trigger will be separately checked for the two cohorts:

1. Severe state cohort
2. Moderate state cohort

The antiplatelet protocol appendix includes pre-specified subgroups/cohorts defined by their classification at baseline with the most recent data available about the patient state at baseline with potentially different outcomes.

4.5. Endpoint Adjudication

Thrombosis and bleeding endpoints will be centrally adjudicated. Preliminary reports may describe data available prior to completion of adjudication where necessary.

5. Unblinding

The development of the analysis plan was carried out prior to unblinding of study investigators. Using guidance from the Data and Safety Monitoring Board, the NHLBI will decide when the Investigators will be unblinded to study outcomes by treatment.

6. Analysis Populations

For the purpose of this SAP, several analysis populations are defined.

1. **Study Confirmed (Primary)**. The study's primary analysis population includes all enrolled patients with confirmed COVID-19 randomized to either intervention and analyzed according to the intention-to-treat principle (i.e. according to randomly assigned treatment status, irrespective of actual treatment receipt).

It is recognized that the primary analysis includes both patient cohorts, moderate and severe. This analysis population will remain primary even if one cohort has a trigger before the other cohort. This is because the primary analysis uses Bayesian borrowing in which the outcomes from both cohorts are used to estimate the separate ORs for each cohort. The primary analysis will be conducted by the unblinded statistician and the results for the cohorts with statistical triggers will be reported to the DSMB.

If both cohorts achieve a superiority or futility threshold at the same time, a sensitivity analysis including a common OR which assumes a single treatment effect irrespective of

illness severity, adjusted for severity, will be conducted, in addition to the primary analysis of each cohort.

The following analysis populations will be defined for each public disclosure corresponding to each cohort being unblinded.

1. **Study Confirmed Unblinded.** The subset of patients in the study confirmed population that belong to the cohort(s) being reported (i.e. those specific cohort(s) that have been unblinded for reporting). This population consists entirely of patients with laboratory-confirmed COVID-19 randomized to P2Y12 or usual care and analyzed according to the intention-to-treat principle.

2. **Study Confirmed and Suspected Unblinded** (exploratory sensitivity analyses only)**.** The study population including patients with suspected but unconfirmed COVID-19 who belong to the cohorts that are unblinded for reporting.

3. **Study per Protocol for P2Y12.** This consists of the patients in the study confirmed unblinded population who have been treated as per protocol. In this analysis that is defined as 1) patients randomized to P2Y12 and who received at least 1 dose by Day zero (day of randomization) or Day 1 (first full day after randomization, **and** 2) patients randomized to usual care who did not receive a P2Y12 dose on or by the end of first full study day after randomization (Day 1).

4. **Study per Protocol for P2Y12 and Heparin.** This consists of the patients in the study confirmed unblinded population who have been treated as per protocol for P2Y12 and with the suggested dose of heparin anticoagulation according to illness severity (moderate or severe) at the time of randomization. In this analysis that is defined as 1) patients randomized to P2Y12 and who received at least 1 dose by day zero (day of randomization) or Day 1 (first full day after randomization, **and** 2) patients randomized to usual care who did not receive a P2Y12 dose on or by the end of first full study day after randomization (Day 1), **and** 3) patients who received at least 1 dose of therapeutic anticoagulation by Day 1 if moderate state cohort, or at least one dose of prophylactic anticoagulation by Day 1 if severe state cohort.

7. Endpoints

The following endpoints will be analyzed, displayed graphically, and summarized with descriptive statistics.

1. **Organ Support-Free Days** (OSFDs)
   - This is the primary endpoint for the study, and is a composite ordinal endpoint reflecting the number of days alive and off organ support, with in-hospital mortality from any cause as the worst possible outcome. Organ support considered is cardiovascular (vasopressor/inotrope support) and respiratory support (high flow nasal cannula, invasive or non-invasive ventilation, or ECMO). In-hospital death is considered a –1 and may occur after study day 21 as long as it occurs during the index hospitalization through day 90.
   - Detailed definitions for OSFDs are specified in the study data dictionary.

- Missing values for organ support-free days will be treated as "missing and will not be included in the analyses". We will conduct a sensitivity analysis on the primary endpoint treating missing values using the "last known status carried forward" approach.

2. **Categorization of the Primary Endpoint - Organ Support-Free Days**
   - Three-level outcome: No organ support without death, organ support without death, death

3. **In-Hospital Mortality**
   - A dichotomous endpoint of in-hospital death from any cause where the death component corresponds to a –1 on the OSFD endpoint. The measurement of in-hospital mortality is truncated at 90 days.

4. **28-Day Mortality**
   - A dichotomous endpoint of death from any cause. The measurement of mortality is not dependent on the location of the patient.

5. **Mortality**
   - This is a time-to-event endpoint through 90 days.
   - Any patient currently in the hospital or transferred on organ support to an alternative care facility will be censored at their last known status alive, regardless of location at the time of that last assessment.
   - Any patient successfully discharged from hospital, alive, without organ support, will be censored at the date of discharge if 90-day mortality data are not yet recorded.

6. **Vasopressor/Inotrope-Free Days to Day 28**
   - An ordinal outcome of the number of days alive and free of vasopressor/inotropes. This is the exact calculation of OSFD, with vasopressor/inotropes as the only organ support category. In-hospital death is considered a 0 (consistent with ACTIV-4A protocol 1.0).
   - All platforms compute vasopressor-free days based on integer days on which vasopressors/inotropes were not received at any time
   - Vasopressor/inotrope-free days will be computed based on the duration of time from the initiation of vasopressors/inotropes to the final cessation of vasopressors/inotropes during the 28-day period; intervening days on which patients are not on vasopressors/inotropes will be ignored

7. **Ventilator-Free Days to Day 28**
   - An ordinal outcome of the number of days alive and free of ventilation. This is the exact calculation of OSFD, with invasive or non-invasive ventilation as the only organ support category. In-hospital death is considered a 0 (consistent with ACTIV-4A protocol 1.0).
   - All platforms compute ventilator-free days based on integer days on which invasive or non-invasive ventilation were not received at any time.
   - Ventilator-free days will be computed based on the duration of time from the initiation of mechanical respiratory-support free days to the final cessation of

invasive or non-invasive ventilation during the 28-day period; intervening days on which patients are not on ventilatory support will be ignored.

8. **Renal Replacement-Free Days to Day 28**
   - Restricted to patients not on Renal Replacement Therapy at baseline
   - An ordinal outcome of the number of days free of renal replacement therapy in the hospital. In-hospital death is considered a 0 (consistent ACTIV-4A protocol 1.0).
   - Compute renal replacement-free days based on integer days on which renal replacement therapy was not received at any time
   - Renal replacement-free days will be computed based on the duration of time from the initiation of renal replacement therapy to the final cessation of renal replacement therapy during the 28-day period; intervening days on which patients are not on renal replacement therapy will be ignored

9. **Time to ICU Liberation**
   - Within the severe cohort
   - Time until the last day in the ICU (including days between ICU stays not in the ICU).This variable will be truncated at 90 days.
   - Deaths in ICU at any time will censored at 90 days.
   - Patients still in the ICU at data snapshot will be considered censored at the time of exposure (at the time the snapshot was taken).

10. **Duration of Hospital Stay**
    - A time-to-event endpoint of leaving the hospital alive. If a patient is known to leave and return to the hospital within 21 days that intervening time will be ignored.
    - This variable will be truncated at 90 days.
    - Patients who die in hospital at any time will be considered censored at 90 days.
    - Patients still in the hospital at data snapshot will be considered censored at the time of exposure (at the time the snapshot was taken).

11. **Deep Venous Thrombosis**
    - A dichotomous endpoint of clinically detected deep venous thrombosis diagnosed at any time during the index hospitalization.

12. **Pulmonary Embolism**
    - A dichotomous endpoint of clinically detected pulmonary embolism diagnosed at any time during the index hospitalization.

13. **Ischemic Cerebrovascular Event**
    - A dichotomous endpoint of ischemic cerebrovascular event (stroke).
    - Assessed during the index hospitalization, up to 28 days

14. **Acute Myocardial Infarction**
    - A dichotomous endpoint of acute myocardial infarction defined according to the universal definition of myocardial infarction.
    - Assessed during the index hospitalization, up to 28 days

15. **Systemic Arterial Thromboembolism**
    - A dichotomous endpoint of clinically diagnosed systemic arterial thrombosis or embolism
    - Assessed during the index hospitalization, up to 28 days

16. **Major Thrombotic Event or Death**
    - A composite dichotomous endpoint of pulmonary embolism, ischemic cerebrovascular event, myocardial infarction, or systemic arterial thromboembolism diagnosed at any time during the index hospitalization or death in hospital

17. **All Thrombotic Events or Death**
    - A composite dichotomous endpoint of deep vein thrombosis, pulmonary embolism, ischemic cerebrovascular event, myocardial infarction, or systemic arterial thromboembolism diagnosed at any time during the index hospitalization or death in hospital

18. **Hospital Re-Admission**
    - A dichotomous endpoint of readmission to hospital within 90 days from randomization
    - This endpoint will be reported descriptively using proportions, including for diagnoses of interest: bleeding, thrombotic event, MI, stroke, ventilatory support

19. **Renal Replacement Therapy**
    - A dichotomous endpoint of initiation of renal replacement therapy during the index hospitalization among patients not on renal replacement therapy at baseline.
    - Censored at day 90

20. **Acute Kidney Injury**
    - A dichotomous endpoint of acute kidney injury (as defined by KDIGO) or requirement of renal replacement therapy during the index hospitalization among patients not on renal replacement therapy at baseline.
    - Censored at day 90

21. **The World Health Organization (WHO) 8-Point Ordinal Scale, Value on Day 14.**
    - A modified WHO ordinal scale will be used:
      0 + 1 + 2 = No longer hospitalized
      3 = Hospitalized, no oxygen therapy
      4 = Oxygen by mask or nasal prongs
      5 = Non-invasive ventilation or high-flow oxygen
      6 = Intubation and mechanical ventilation
      7 = Ventilation + additional organ support: vasopressors, renal replacement therapy (RRT), ECMO
      8 = Death

22. **28-Day Outcome of Mechanical Ventilation or Death**

- Binary variable to indicate whether the patient is on a ventilator on Day 28 or deceased by day 28.
- Those discharged before day 28 who are not on a ventilator will considered to not be on a ventilator at day 28.

23. **Ventilator Support or Death**
    - Dichotomous indicator of requiring all ventilator support (invasive and non-invasive) or death over the index hospital stay
    - Truncated at 90 days

24. **Any Organ Support or Death**
    - Dichotomous indicator of requiring any organ support or death over the index hospital stay
    - Truncated at 90 days

25. **Four-Level Organ Support**
    - Four-level ordinal outcome: No organ support without death, organ support without invasive mechanical ventilation and without death, invasive mechanical ventilation without death, death

26. **Categorization of the Endpoint–Mechanical Ventilatory Support-Free Days**
    - Three-level outcome: No mechanical ventilatory support without death, mechanical ventilatory support without death, death
    - HFNO is not counted as ventilatory support

27. **Major Thrombotic or Critical Bleeding Composite Event by Day 28**
    - At 28 days, a binary indicator of
    - Death or
    - Major thrombotic event or
    - Critical major bleed (fatal, ICH, Bleeding in critical area or organ)

28. **Any Thrombotic or Bleeding Composite Event by Day 28**
    - At 28 days, a binary indicator of
    - Death or
    - Any thrombotic event (major thrombotic event or DVT) or
    - Major ISTH bleed

29. **EQ-5D**
    - At 90 days, continuous measure of function

30. **Symptoms**
    - Binary indicator of any of the following symptoms: fatigue, cough, dyspnea, and chest pain.
    - Repeat analysis for each individually
    - At 90 days
    - Among those that did not have the symptom prior to COVID-19 illness based on the appropriate eCRF

Safety Endpoints

1. **Major Bleeding on or before Day 17**
    - A dichotomous endpoint of major bleeding as defined according to the "International Society of Thrombosis and Hemostasis (ISTH) criteria in non-surgical patients."
    - The endpoint is censored at 17 days to correspond to the intervention duration.

2. **Critical Major Bleeding on or before Day 17**
    - A dichotomous endpoint of critical bleeding as defined by 2 components of the ISTH major bleeding definition (fatal bleeding or bleeding in critical area or organ)
    - The endpoint is censored at 17 days to correspond to the intervention duration.

3. **Intracranial Hemorrhage**
    - A dichotomous endpoint of intracranial hemorrhage.
    - Assessed during the index hospitalization, up to 28 days

4. **Major Bleeding at 28 Days**
    - At 28 days, a binary indicator of any major bleeding (fatal bleeding or bleeding in critical area or organ) or death defined by defined the "International Society of Thrombosis and Hemostasis (ISTH) criteria in non-surgical patients."

5. **Heparin-Induced Thrombocytopenia (HIT)**
    - A dichotomous endpoint of laboratory-confirmed HIT.
    - The endpoint is censored at 17 days to correspond to the intervention duration.

8. Graphical Data Summaries

1. All ordinal endpoints will be plotted using stacked cumulative bar plots and cumulative probability plots.
2. All time-to-event endpoints will be plotted using Kaplan-Meier or cumulative incidence plots. Positive clinical event outcomes will be plotted as the cumulative rate of event, and negative events will be plotted as the cumulative rate of event-free.

9. Descriptive Statistics

1. Ordinal endpoints will be summarized by the cumulative frequency of each outcome. The 5th, 25th, 50th, 75th, and 95th percentiles will be summarized.
2. Dichotomous endpoints will be summarized by the proportion in each category.
3. Time-to-event outcomes will be summarized by the 2.5th, 10th, 25th, 50th, 75th, 90th, and 97.5th percentiles from the Kaplan-Meier or cumulative incidence estimates, as available.

10. Baseline Characteristics and Co-Interventions

The following demographics will be summarized across arms. More may be added as baseline summaries: Age, sex, BMI, race, ethnicity, illness severity at admission, pre-existing

conditions, baseline use of oxygen (no, 2L or less, more than 2L but less than 20 L/min, high flow) non-invasive ventilation, invasive mechanical ventilation, ECMO, vasopressors/inotropes, renal replacement therapy, and miscellaneous physiological values and inflammatory biomarker laboratory values. P2Y12 (specific agent used) by the first full day will be compared between groups. Additionally, exposure to relevant drugs (e.g., aspirin, steroids, immunomodulatory therapies, anticoagulation drug and dose) prior to hospitalization, at baseline, and during the treatment period will be compared between groups.

11. Adherence

Adherence will be assessed based on the proportion of patients receiving a P2Y12 agent consistent with their randomly assigned strategy by the end of the first full study day after randomization.

12. Analytic Approach

Each inferential analysis will be done using a Bayesian model. Some default frequentist methods are used for exploration and description. A summary of the analyses methods is provided below. Events that occur at low frequency will be reported descriptively and not modelled.

12.1. Primary Analysis of Primary Endpoint

The **primary analysis model** is a Bayesian cumulative logistic model for the ordinal primary endpoint. The model is described below.

The primary endpoint has 23 possible ordered outcome values. Let the outcome for a patient labeled as Y_i, with possible values, –1 (death), 0, 1, …, 21. A cumulative logistic model is specified. The model is structured so that an OR >1 implies clinical benefit. The model has factors for:

1. Each level of the ordinal endpoint
2. Each global site, nested within country
3. Age; ≤39, 40-49, 50-59, 60-69, 70-79, 80+
4. Sex
5. Time: 2-week epoch bins of time working backwards from the last enrolled patient.
6. Hx cardiovascular disease, including HTN, and/or diabetes
7. Severe/Moderate cohort
8. For severe (type of organ support, invasive mechanical ventilation vs. not)
9. For moderate (the amount of O2 required, no O2, > 0 but <= 2, >2)
10. An effect for each intervention; the effects for P2Y12 are nested across cohorts
11. All sites within a country that have <5 patients randomized will be combined into a single site within that country.
12. For the primary outcome, if there is an outcome in the ordinal scale that did not occur in the data, then that outcome will be combined the next worse outcome. This is done for model stability. For example, if the outcome 11 never occurred, then a combined outcome of 10 & 11 will be modeled for the analysis.

13. If analyzing a single cohort that does not allow for the hierarchical structure in the model, the hierarchical structure of the treatment parameters will be replaced by a standard normal prior unless otherwise specified.

The primary analysis model will be referenced with certain model assumptions for sensitivity analyses. For example, the “time effects” in the model could be assumed to be 0.

12.2. Proportional Odds Assumption

The primary analysis model is based on an assumption of a proportional effect of treatment across the scale of the ordinal outcome. In order to assess the robustness of the results to this assumption, a dichotomous model is fit to every level of the ordinal outcome across the scale and the OR for each dichotomous break is presented. If the probabilities for the tails of the ordinal endpoint have small probabilities (<5%) they may not be conducted. No statistical test of proportional odds will be conducted.

12.3. Analytic Approach for Secondary Dichotomous Endpoints

A Bayesian logistic regression model will be used for each dichotomous outcome. The model will always specify the “event” as the negative outcome and be parameterized so that an OR >1 implies benefit to patients. The model is the standard logistic link function model:

$$log\ \left(\frac{\pi}{1-\pi}\right) = \alpha - [factors]$$

References will be made to the factors in the model and their prior distribution. Many of these factors will be the same as the primary analysis model, with the same priors, as the parameters have similar interpretation. For example, all in-hospital mortality models should use the Beta prior distribution implied by the Dirichlet prior in the OSFD model. If not otherwise specified, the prior distribution for the main effect is $\alpha \sim N(0, 1.82^2)$ (similar to a uniform prior on the probability scale). Dynamic borrowing is not employed for secondary outcomes, except for survival to hospital discharge.

12.4. Analytic Approach for Secondary Time-To-Event Endpoints

All inferential time-to-event analyses will be done using a Bayesian piecewise exponential model. The Bayesian time-to-event model is intended to mirror a Cox proportional hazards model, with the underlying hazard rate modeled with a piecewise exponential model. The underlying hazard will be modeled with a hazard rate for each 10-day period in the model. The prior distribution for the hazard rate for each day is a gamma distribution with 1 day of exposure and a mean equal to the total exposure divided by the total number of events. This prior will have very little weight but will provide numerical stability to the model. Each factor is incorporated as a proportional hazard rate through an additive linear model of the log-hazard. The default prior for each factor is the same as for the log-odds in the ordinal model. If other non-specified variables are added to the model, then a normal distribution with mean 0 and standard deviation 10 will be utilized.

12.5. Analytical Approach for Cohort Analyses

The analyses for each cohort uses the same analysis for the primary models (ordinal, dichotomous, and time-to-event) with the following differences. For each model the treatment effect is modeled separately and independently in each defined cohort. A single group will be selected as the group to have a main effect treatment effect, modeled with a normal distribution with mean 0 and standard deviation 10 (for the log-odds or log-hazard ratio). This group is either the largest group or the first subgroup. Each additional group will

have an additive effect on the log parametrization scale with independent normal distribution priors with mean 0 and standard deviation 10.

If multiple subtypes are reported in a single analysis, each group within each subtype will be modeled independently without Bayesian borrowing across subtypes for the treatment effect.

12.6. Markov Chain Monte Carlo (MCMC) Model Stability

The Bayesian models have many parameters and there may be a risk of poor model stability, including convergence and mixing behavior of the MCMC sampler. These instabilities may be based on sparse data on the outcome or covariates. The statisticians running the model may make changes that do not affect the overall interpretation but provide reliable model diagnostics and scientific rigor. Any alterations will be noted.

12.7. Model Outputs

The standard model outputs for each treatment effect will be the mean, standard deviation, median, and 95% credible intervals (all credible intervals will be equal-tailed intervals, so 95% credible intervals will range from the 2.5^{th} percentile to the 97.5^{th} percentile of the posterior distribution). For the ordinal endpoints, the odds ratios will be summarized. For the dichotomous endpoints, the odds ratios will be summarized. For the time-to-event endpoints, the hazard ratios will be summarized.

For each inferential model, a posterior probability that one arm is superior will be provided for each comparison between arms. This posterior probability has been identified as the primary analysis metric between arms. A posterior probability greater than 99% of superiority or inferiority has been identified as statistically significant. For futility a threshold of 95% has been specified.

12.8. Exploratory Analyses

Exploratory analyses after unblinding will not be considered inferential and no p-values will be presented. Any post-hoc exploratory analyses will use the following methods:

1. Ordinal endpoints will be compared using a cumulative proportional odds model with summaries of the OR, 95% confidence intervals, and Wilcoxon tests for robustness against a lack of proportional odds.
2. Time-to-Event analyses will utilize a Cox proportional hazards model, summarizing the hazard ratios and 95% confidence intervals.
3. Continuous endpoints will compare means with 95% confidence intervals based on two-sample t-test procedures.
4. Dichotomous proportions will be compared using logistic regression summarizing the OR and 95% confidence intervals. Differences between proportions will be summarized using observed differences and normal approximations for the 95% credible intervals.

12.9. Handling of Missing Data

For the primary endpoint of OSFDs missing primary outcomes will be ignored. A sensitivity analysis is conducted where last status carried forward is used for imputation. Patients with missing age, date of randomization, sex or treatment assignment will be ignored. For additional endpoints those patients missing the endpoint will be ignored (for time to event analyses censoring will be used and aren't considered missing). For the subgroup analyses patients with missing subgroup variables will be lumped into a single group of "missing" in addition to the subgroup classifications.

For time-to-event analyses patients that have a competing event that dictates they cannot achieve a positive outcome (like death for time to hospital discharge), the patient will be assigned a no positive event outcome at the maximum time for the analysis. Patients not achieving an absorbing event that become lost-to-follow up will be censored at the last known time (unless otherwise specified for the analysis).

12.10. Definition of Times

Adherence and per protocol analyses will rely on assessment of drug administration by the end of the first full study day after randomization. Post-randomization day 1 is referred to as study day 1 and constitutes the 24-hour period commencing at midnight of the day after randomization.

12.11. Post-Randomization Analyses

Participants who are randomized to receive one strategy may in fact be treated with another strategy based on health status and provider discretion. Co-interventions during the treatment period (e.g., aspirin, corticosteroids, IL6 antagonists, anticoagulants and their dosing) may modify the benefit or harm of P2Y12. Exploratory analyses will estimate the causal effect of the treatment for these patients using marginal structural modelling techniques. These techniques use inverse probability weighting methods that are based on patient-level covariates to create comparable groups for the analysis.

Anticoagulant treatment will be classified as based on the following dosing equivalents categorization: (1) standard prophylactic, (2) intermediate prophylactic, (3) subtherapeutic, and (4) therapeutic. Based on sample size, may collapse groups for analyses

12.12. Heterogeneity of Treatment Effect

The heterogeneity of the treatment effect will be evaluated in the following a priori defined subgroups:

- Age (< 50 years, 50-70 years, > 70 years)
- Sex
- Invasive mechanical ventilation at baseline for severe cohort
- For moderate (the amount of O2 required, no O2, > 0 but <= 2, >2)
- Heparin dosing during day 1. Categorized as therapeutic if defined as therapeutic at any time during day 0 or 1.

Additional exploratory subgroup analyses will include the following:

- Race/Ethnicity
- D-dimer levels
- Markers of inflammation (CRP, ferritin)
- Baseline troponin
- BMI
- Shock (use of vasopressors or inotropes at baseline)
- Baseline chronic kidney disease
- Steroid administration for COVID-19 at baseline
- Il-6 inhibitor administration for COVID-19 at baseline
- If additional treatments are determined through other research to be beneficial before unblinding subgroups will be defined by those treatments.
- Sites with at least 80% ticagrelor use as assigned P2Y12 inhibitor vs. sites with less than 80% ticagrelor use

- Geographic (Europe, US+Canada, Central and South America)

12.13. Adaptive Analyses

The goal for the P2Y12 arm is to determine if it is a safe and effective treatment for hospitalized patients with COVID-19.

Efficacy and Futility Monitoring Plan for P2Y12 Anti-Platelet treatment
The efficacy goal of the P2Y12 arm is to determine if it improves the clinical outcome for hospitalized patients with COVID-19 as measured by the primary outcome, organ-support free-days (OSFDs). There is also the key secondary efficacy goal which is to understand if P2Y12 reduces major thrombotic events and increases bleeding rates.

The hypothesis that P2Y12 reduces organ dysfunction through inhibition of microthrombi in capillaries led to the selection of the primary OSFD outcome. The thrombotic endpoint is testing the hypothesis that the risk of arterial and venous (small, medium and large vessel) thrombosis is reduced with P2Y12.

Key Secondary Efficacy endpoint: a composite major thrombotic event (TE) endpoint of death, pulmonary embolism, systemic arterial thromboembolism, myocardial infarction, or ischemic stroke collected during hospitalization or at 28 days after enrollment.

Stopping Rules for Efficacy
The trial has been designed to separately test the efficacy of P2Y12 with respect to OSFD within two patient cohorts: patients with moderate or severe illness.

The P2Y12 arm will be declared superior to the usual care arm within a specific cohort if the posterior probability of superiority within the cohort is greater than 0.99.

There are no efficacy stopping boundaries for a cohort based on the key secondary endpoint of major thrombotic events or death. The key secondary endpoint is addressed only when futility has been declared for the organ-support free-day endpoint.

Stopping Rules for Futility
The P2Y12 arm would meet a stopping rule for futility for the primary OFSD endpoint only.

If, within a patient cohort, there is a 5% or less posterior probability that the OFSD odds-ratio for the P2Y12 arm compared to the usual care arm is greater than 1.2, favoring the P2Y12 arm, then P2Y12 would meet futility in the cohort.

Safety Monitoring Plan for P2Y12 Anti-Platelet Treatment
A safety analysis for the two arms will be presented to the DSMB. The safety events of importance for the P2Y12 arm are a potential excess of major bleeding events, defined by the International Society on Thrombosis and Haemostasis (ISTH) criteria, including ICH and fatal bleeds.

ISTH Defined Major Bleeding
Bleeding that:
1. Resulted in death,
2. Intracranial hemorrhage

3. Occurred in a critical location (intraspinal, intraocular, retroperitoneal, intraarticular, intramuscular with compartment syndrome, or pericardial), or
4. Was associated with either a decrease in the hemoglobin level of at least 2 g per deciliter or transfusion of at least 2 units of packed red cells

A potential safety event for P2Y12 is excess thrombotic events and is a key secondary efficacy endpoint.

Major thrombotic events are defined as: A composite at day 28 or hospital discharge (whichever is first) of death, pulmonary embolism (PE), systemic arterial thromboembolism, myocardial infarction (MI), or ischemic stroke.

Pre-specified stopping guidelines for major bleeding events are not specified. Any decision on stopping the P2Y12 arm would need to balance the severe bleeding risk (e.g., fatal bleeding or intracranial bleeding) with any benefit on the primary endpoint, and reduction in TE and the need for organ support. We leave any decision on recommending stopping the P2Y12 arm for safety reasons to the DSMB given the risk/benefit profile. We have not provided a specific absolute or relative difference for the rates of severe bleeding between the groups as criteria for recommending stopping. Fatal bleeding or intracranial bleeding are the safety events of greatest importance.

It may be appropriate to assess risk factors associated with severe adverse events such as fatal bleeding or intracranial hemorrhage and recommend modification of eligibility criteria based on net clinical benefit. Therefore, recommendations might include changing the eligibility criteria to exclude patients at highest risks for these severe adverse events. Due to the uncertainty around rates in COVID-19 patients, it is possible that after seeing the rates in the current trial, the DSMB would request additional information from the unblinded statistician and/or the investigators before making a definitive recommendation about changing eligibility criteria or stopping an arm entirely.

There is a hierarchy of clinical importance of various TEs, with DVT in the absence of PE the least important and PE, stroke, systemic arterial embolism, and MI (all of which will be adjudicated) more clinically important. PE that impacts oxygenation or hemodynamics may impact the primary endpoint. The trial should not stop before a determination of a difference or not on the primary outcome has been reached, unless one of the two arms presents a clear safety risk, outweighing any potential efficacy benefit.

To aid the DSMB in the risk/benefit and safety determination the following analyses will be presented:

Safety Event	Quantity	P2Y12	Standard Care
**ISTH Major Bleeding Events**	Number of Events	X1	X2
	Number At 28-days	N1	N2
	Event Rates	X1/N1	X2/N2
	95% Confidence Interval (CI)	LB1, UB1	LB2, UB2
	Difference in Rates	Difference; 95% CI	
	Odds-Ratio	OR; 95% CI	
**ICH and Fatal Bleeds**	Number of Events	X1	X2
	Number At 28-days	N1	N2
	Event Rates	X1/N1	X2/N2
	95% Confidence Interval (CI)	LB1, UB1	LB2, UB2
	Difference in Rates	Difference; 95% CI	
	Odds-Ratio	OR; 95% CI	
**Major Thrombotic Events (TE)***	Number of Events	X1	X2
	Number At 28-days	N1	N2
	Event Rates	X1/N1	X2/N2
	95% Confidence Interval (CI)	LB1, UB1	LB2, UB2
	Difference in Rates	Difference; 95% CI	
	Odds-Ratio	OR; 95% CI	
**All-Cause Mortality**	Number of Events	X1	X2
	Number At 28-days	N1	N2
	Event Rates	X1/N1	X2/N2
	95% Confidence Interval (CI)	LB1, UB1	LB2, UB2
	Difference in Rates	Difference; 95% CI	
	Odds-Ratio	OR; 95% CI	

*PE, stroke, systemic arterial embolism, and MI

These analyses are in addition to the efficacy and futility analyses that will be presented for the primary efficacy endpoint of organ-support free-days.

13. Models Reporting Outlines

13.1. Primary Analysis of OSFDs

Population	
Endpoint	
Model	
Factors	

The following posterior probabilities will be reported for each subtype being reported:

Quantity of Interest	Posterior Probability
P2Y12 is superior to control	
P2Y12 is futile	
P2Y12 is inferior	

The following will be reported:

Odds Ratio Parameter	Mean	SD	Median	95% Credible Interval
Age < 39				
Age 40, 49				
Age 50, 59				
Age 70-79				
Age 80+				
Female				
Time epoch 1				
…				
Time epoch k-1				
Hx cardiovascular disease/diabetes				
Invasive medical ventilation (severe only)				
Oxygen >0 but <=2 (moderate only				
Oxygen > 2 (moderate only)				
Country				
P2Y12				
