# Supplementary material for: Effect of P2Y12 Inhibitors on Organ Support–Free Survival in Critically Ill Patients Hospitalized for COVID-19: A Randomized Clinical Trial
Source: JAMA Netw Open. 2023 May 25;6(5):e2314428. doi: 10.1001/jamanetworkopen.2023.14428 (PMC10214036; doi:10.1001/jamanetworkopen.2023.14428)
Supplement: Supplement 2. — eTable 1. Comparison Between Bayesian and Frequentist Analyses eTable 2. Participants’ P2Y12 Inhibitor Type and Dosage Used eTable 3. Sensitivity Analyses of the Primary Outcome eTable 4. Subgroup Analysis on the Primary Endpoint (Organ Support Free Days to Day 21) eTable 5. Thrombotic Events Confirmed by Adjudication by Treatment Group eTable 6. Component Criteria on Which Major Bleeding Events Were Confirmed as Meeting the Definition of a Major Bleed Proposed by the International Society on Thrombosis and Hemostasis by Treatment Arm eAppendix 1. Investigators and Collaborators eAppendix 2. Supplemental Methods eReferences [file jamanetwopen-e2314428-s002.pdf]

Supplementary Online Content

Berger JS, Neal MD, Kornblith LZ, et al; ACITV-4a Investigators. Effect of P2Y12 inhibitors on organ support–free survival in critically ill patients hospitalized for COVID-19: a randomized clinical trial. *JAMA Netw Open*. 2023;6(5):e2314428. doi:10.1001/jamanetworkopen.2023.14428

eTable 1. Comparison Between Bayesian and Frequentist Analyses

eTable 2. Participants' P2Y12 Inhibitor Type and Dosage Used

eTable 3. Sensitivity Analyses of the Primary Outcome

eTable 4. Subgroup Analysis on the Primary Endpoint (Organ Support Free Days to Day 21)

eTable 5. Thrombotic Events Confirmed by Adjudication by Treatment Group

eTable 6. Component Criteria on Which Major Bleeding Events Were Confirmed as Meeting the Definition of a Major Bleed Proposed by the International Society on Thrombosis and Hemostasis by Treatment Arm

eAppendix 1. Investigators and Collaborators

eAppendix 2. Supplemental Methods

eReferences

This supplementary material has been provided by the authors to give readers additional information about their work.

© 2023 Berger JS et al. *JAMA Network Open.*

**eTable 1. Frequentist analysis compared to Bayesian analysis of results**[a]			
**Endpoint**	**Bayesian (with borrowing)** [b]	**Bayesian (no borrowing)**[c]	**Frequentist** [c]
Days free of organ support (primary endpoint)	1.07 (0.85 - 1.33)	1.09 (0.86 - 1.38)	1.07 (0.85 - 1.34)
Survival to hospital discharge (up to 90 days)	1.15 (0.84 - 1.55)	1.16 (0.84 - 1.62)	1.14 (0.84 - 1.54)

[a] Models are adjusted for age, sex, enrollment epoch, cardiovascular disease (composite of hypertension, heart failure, coronary artery disease, peripheral artery disease, cerebrovascular disease), SGLT2 assignment, and baseline mechanical ventilation; study country and site are treated as nested random effects.
[b] Bayesian model includes the 949 critically ill patients and borrowing from the 562 noncritically ill patients
[c] models are performed with the 949 critically ill patients

© 2023 Berger JS et al. *JAMA Network Open.*

**eTable 2 P2Y12 inhibitor administered, and dosage used**	
**Characteristic**	**(N=479)**
Antiplatelet drug, No./total (%)	472/479 (98.5)
Ticagrelor	372/472 (78.8)
60mg twice daily	218/351 (62.1)
90mg twice daily	133/351 (37.9)
Clopidogrel	100/472 (21.2)
Loading dose of clopidogrel	82 (82.0)
Percent of time on study drug post-randomization, median (IQR)[a]	100 (100-100)
Percent of time on study drug post-randomization, mean (SD)[a]	89.3 (23.9)
[a] P2Y12 inhibitor was recommended for 14 days or until hospital discharge, whichever was sooner (N=470)	

© 2023 Berger JS et al. *JAMA Network Open.*

eTable 3. Sensitivity Analyses of the Primary Outcome among Critically Ill Participants				
**Sensitivity Analysis[a]**	**P2Y12 Inhibitor (N=475)**	**Usual Care (N=468)**	**Adjusted Median Proportional OR (95% CrI)[b]**	**Posterior Probability of Superiority of P2Y12 Inhibitor**
Examining the primary outcome as a three-category ordinal outcome[c]			1.03 (0.79 - 1.33)	58.3%
Alive with organ support without mechanical ventilation	197 (41.5%)	201 (42.9%)		
Alive with mechanical ventilation	157 (33.1%)	138 (29.5%)		
Death	121 (25.5%)	129 (27.6%)		
Examining the primary outcome as a four-category ordinal outcome[d]			1.03 (0.81 - 1.31)	59.4%
Alive with organ support without mechanical ventilation	197 (41.5%)	201 (42.9%)		
Alive with non-invasive mechanical Ventilation	70 (14.7%)	62 (13.2%)		
Alive with invasive mechanical Ventilation	87 (18.3%)	76 (16.2%)		
Death	121 (25.5%)	129 (27.6%)		
Excluding participants receiving therapeutic anticoagulation[e]	(n=432)	(n=417)	1.00 (0.78 - 1.31)	50.7%
Organ support-free days, Median (IQR)	12.0 (-1.0 - 17.0)	12.0 (-1.0 - 18.0)		

[a] Analysis of all critically ill participants
[b] Effect estimates are adjusted for age, sex, enrollment epoch, cardiovascular disease (composite of hypertension, heart failure, diabetes, coronary artery disease, peripheral artery disease, cerebrovascular disease), SGLT2 assignment, and baseline mechanical ventilation; study country and site are treated as nested random effects.
[c] Levels of the ordinal outcome are: 1) alive with organ support without mechanical ventilation; 2) alive with mechanical ventilation; and 3) death during hospitalization.
[d] Levels of the ordinal outcome are: 1) alive with organ support without mechanical ventilation; 2) alive with non-invasive mechanical ventilation without invasive mechanical ventilation; 3) alive with invasive mechanical ventilation; and 4) death during hospitalization.
[e] 84 participants were excluded who did not receive therapeutic anticoagulation by the end of day 1 after randomization

© 2023 Berger JS et al. *JAMA Network Open.*

eTable 4 Moderator analysis of the effect of P2Y12 inhibitor on primary outcome					
		Unadjusted		Adjusted[d]	
**Subgroup**	**Participants**	**OR (95% CI)[b]**	**P[c]**	**OR (95% CI)[b]**	**P[c]**
Age[i]	N = 949		0.064		0.019
< 65 years	707 (74.5)	1.17 (0.90 to 1.51)		1.23 (0.95 to 1.60)	
≥ 65 years	242 (25.5)	0.71 (0.45 to 1.12)		0.66 (0.41 to 1.04)	
Sex	N = 949		0.71		0.79
Female	346 (36.5)	1.00 (0.69 to 1.45)		1.05 (0.72 to 1.53)	
Male	603 (63.5)	1.09 (0.82 to 1.44)		1.12 (0.84 to 1.49)	
Race	n = 869		0.83		0.96
Black	190 (21.9)	0.99 (0.60 to 1.64)		1.04 (0.63 to 1.74)	
White	561 (64.6)	0.96 (0.72 to 1.29)		0.97 (0.72 to 1.30)	
Other[e]	118 (13.6)	1.19 (0.62 to 2.29)		1.05 (0.54 to 2.03)	
Country	N = 949		0.98		0.92
Brazil	41 (4.3)	0.92 (0.30 to 2.78)		0.98 (0.32 to 2.98)	
Italy	35 (3.7)	0.91 (0.29 to 2.86)		1.33 (0.68 to 2.59)	
Mexico/Spain[f]	96 (10.1)	1.15 (0.59 to 2.24)		1.08 (0.84 to 1.39)	
US	777 (81.9)	1.05 (0.82 to 1.35)		0.89 (0.30 to 2.66)	
Body mass index, kg/m^2	n = 918		0.99		0.73
>30	555 (60.5)	1.06 (0.79 to 1.43)		1.13 (0.84 to 1.52)	
≤30	363 (39.5)	1.06 (0.74 to 1.53)		1.04 (0.71 to 1.51)	
Cardiovascular disease[g]	N = 949		0.30		0.23
Yes	459 (48.4)	0.95 (0.68 to 1.31)		0.94 (0.67 to 1.32)	
No	490 (51.6)	1.20 (0.88 to 1.65)		1.25 (0.91 to 1.70)	
Steroids (baseline)	n = 948		0.69		0.69
Yes	792 (83.5)	1.03 (0.81 to 1.31)		1.07 (0.83 to 1.37)	
No	156 (16.5)	1.16 (0.67 to 2.02)		1.21 (0.69 to 2.10)	
Remdesivir (baseline)	n = 948		0.76		0.78
Yes	574 (60.6)	1.08 (0.81 to 1.44)		1.12 (0.84 to 1.50)	
No	374 (39.4)	1.01 (0.70 to 1.44)		1.05 (0.73 to 1.51)	
Mechanical ventilation (baseline)	N = 947		0.44		0.45
Yes	102 (10.8)	1.38 (0.67 to 2.84)		1.41 (0.70 to 2.84)	
No	845 (89.2)	1.02 (0.81 to 1.30)		1.06 (0.83 to 1.35)	
D-Dimer (baseline)	n = 783		0.34		0.64
≥Twofold the ULN	405 (51.7)	0.96 (0.68 to 1.36)		1.07 (0.75 to 1.53)	
<Twofold the ULN	378 (48.3)	1.23 (0.86 to 1.76)		1.21 (0.84 to 1.73)	
C-reactive protein, mg/L	n = 660		0.06		0.09
>median (118)	329 (49.8)	0.87 (0.59 to 1.28)		0.93 (0.63 to 1.39)	
≤median	331 (50.2)	1.45 (0.99 to 2.13)		1.51 (1.02 to 2.23)	
Site ticagrelor use[h]	n = 944		0.62		0.52
>80%	749 (79.3)	1.08 (0.84 to 1.39)		1.13 (0.88 to 1.46)	
≤80%	195 (20.7)	0.93 (0.57 to 1.53)		0.94 (0.57 to 1.55)	

[a] Data expressed as No. (%).
[b] Model results for the subgroup analyses used a frequentist approach. Odds ratio and 95% confidence interval are presented. Odds ratio >1 corresponds with treatment benefit - associated with increased organ support free days.
[c] Probability value of the interaction term between P2Y12 inhibitor and subgroup.
[d] Adjusted for age, sex, enrollment epoch, cardiovascular disease (composite of hypertension, heart failure, coronary artery disease, peripheral artery disease, cerebrovascular disease), SGLT2 assignment, and baseline mechanical ventilation; study country and site are treated as nested, random effects.
[e] Too few patients within the following racial categories to model separately: Asian, multiple, Native American, Pacific Islander, or unspecified.

© 2023 Berger JS et al. *JAMA Network Open.*

[f] Too few patients in sites located in Mexico to model separately.
[g] Includes hypertension, heart failure, coronary artery disease, peripheral artery disease, or cerebrovascular disease.
[h] Sites were stratified by use of ticagrelor in >80% of participants randomized to a P2Y12 inhibitor.
[i] When age is used as a continuous variable, the Age x P2Y12 treatment interaction was statistically significant as well (P=0.047)

© 2023 Berger JS et al. *JAMA Network Open.*

eTable 5 Adjudicated confirmed thrombotic events during hospitalization				
	**P2Y12 inhibitor (N=479)**		**Usual care (N=470)**	
**Event**	**Patients with at least one event[b]**	**Total events**	**Patients with at least one event[b]**	**Total events**
All thrombotic events, No. (%)[a]	71 (14.8)	92	64 (13.6)	74
Deep vein thrombosis[c]	40 (8.3)	44	41 (8.7)	45
Pulmonary embolism	34 (7.1)	36	21 (4.5)	21
Myocardial infarction	3 (0.6)	3	3 (0.6)	3
Ischemic cerebrovascular	9 (1.9)	9	3 (0.6)	3
Systemic arterial thromboembolism	0 (0.0)	0	2 (0.4)	2

[a] Thrombotic events were adjudicated in a blinded fashion by clinical endpoints committees using consensus definitions (see Supplementary Appendix).
[b] Events are not mutually exclusive.
[c] Excluded from the composite endpoint of major thrombotic events.

© 2023 Berger JS et al. *JAMA Network Open.*

eTable 6 Adjudicated confirmed ISTH major bleeding events during hospitalization		
**Event**	**P2Y12 inhibitor (N=479)**	**Usual care (N=470)**
Major bleeding event, No. (%)	15 (3.1)	14 (3.0)
Overt bleeding causing a fall in hemoglobin of ≥2 g/dL	4 (0.8)	5 (1.1)[c]
Overt bleeding leading to transfusion of ≥2 units of whole blood or red cells	5 (1.0)	8 (1.7)
Symptomatic bleeding in a critical area or organ	3 (0.6)	0 (0.0)
Fatal bleeding	3 (0.6)	2 (0.4)

[a] Bleeding events were adjudicated in a blinded fashion by clinical endpoints committees using consensus definitions (see Supplementary Appendix).

[b] Bleeding criteria are not mutually exclusive for confirmation of ISTH major bleeding.

[c] One patient had two major bleeding events: one meeting Overt bleeding causing a fall in hemoglobin of ≥2 g/dL criteria, the other meeting Overt bleeding leading to transfusion of ≥2 units of whole blood or red cells criteria.

© 2023 Berger JS et al. *JAMA Network Open.*

eAppendix 1 - Investigators and Collaborators

DSMB
Richard C. Becker (Chair); Gregory del Zoppo; Peter Henke; Richard Holubkov; Kim Kerr, Agnes Lee; Fedor Lurie; Sara K. Vesely

Lead Investigators
Judith S. Hochman MD, Matthew D. Neal MD, Jeffrey S. Berger MD

Protocol Development Committee
Judith S. Hochman, MD (Chair), Matthew D. Neal, MD (co-Chair), Jeffrey S. Berger, MD (co-PI), Mikhail Kosiborod, MD, Scott D. Solomon, MD, Harmony Reynolds, MD, Hooman Kamel, MD, Pooja Khatri, MD, Mary Cushman, MD, MSc, Lisa Baumann Kreuziger, MD, Scott Berry, PhD, Michael E. Farkouh, MD, Michelle N. Gong, MD, Kristin Hudock, MD, MSTR, Keri S. Kim, PharmD, Lucy Z. Kornblith, MD, Patrick R. Lawler, MD, MPH, Eric Leifer, PhD, Bryan J. McVerry, MD, Jennifer G. Wilson, MD, Ryan Zarychanski, MD, Jonathan D. Newman, MD, MPH, Marc W. Geraci, MD, Aldo Maggioni, MD, Renato Lopes, MD, PhD, Jose Lopez-Sendon, MD, James F. Luther, Bridget-Anne Kirwan, PhD

National Heart Lung and Blood Institute, National Institutes of Health
Program: W. Keith Hoots, MD, Andrei Kindzelski, MD, PhD, Traci Mondoro, PhD, Antonello Punturieri, MD, PhD, Gail Weinmann, MD, Eric Leifer, PhD, James F. Troendle, PhD, Hannah Lipman, MD

RTI International
Kat Asman, MSPH, Jenny Auman, Shawn Hirsch MPH, Carolyn Huitema, MS, CCRP, RAC, Amy S. Kendrick, RN, MSN, Heather Meier, Tracy L Nolen, DrPH, Kayla Nowak, Sonia Thomas, DrPH,

NYU Grossman School of Medicine/ NYU Langone Health Clinical Coordinating Center and Chairs Office
Judith Hochman (Chair); Jeffrey Berger (PI), Harmony Reynolds, Erinn Hade, Aira Contreras, Stephanie Mavromichalis, Eduardo Iturrate, Margaret Gilsenan, Anna Naumova, Arline Roberts, Jonathan D. Newman, Alair Holden, Britnee Marius, Ewelina Gwiszcz, Samaa Mohamed, Michelle Chang

University of Pittsburgh Data Coordinating Center
Matthew D. Neal (Co-Chair), Christine Leeper, Mary Martinez, Jake Schreiber, Joshua Froess, Andrew D. Althouse, James F. Luther, Edvin Music, Mark W. Geraci, Stephen Wisniewski, Adam Cary, Valena Lundy-Wiggins, Cara Frosch, Bianca Jamar, Leona Saunders

SOCAR Research
Bridget-Anne Kirwan, Sophie de Brouwer, Emilie Perrin, , Giulia Fregni, Gaelle Garcia, Sandra Bula**,** Michael Nelson**,** Céline Daelemans**,** Laurine Paraz

Berry Consultants
Michelle Detry, Anna McGlothlin, Melanie Quintana, Amy Crawford

Adjudication Committee
Brendan Everett, Sean van Diepen, Gregoire Le Gal, Deborah Siegal, Jean-Philippe Galanaud, Sheila Hegde, Yuri Kim, Natalia Rost, Aneesh Singhal

Network Coordinating Centers
ISCHEMIA/MINOCA-HARP/EPPIC-NET: Harmony R. Reynolds (Lead), Aira Contreras, Stephanie Mavromichalis, Margaret Gilsenan, Michelle Chang, Anna Naumova, Danielle Sin, Elhaji Diene, Ewelina Gwiszcz, Isabelle Hogan, Alair Holden, Britnee Marius, Samaa Mohamed, Arline Roberts

PETAL: Michelle Gong (Lead), Nancy Ringwood, Laura Fitzgerald, Haley Morin

Multi-Net: Lucy Kornblith (Lead), Brenda Nunez-Garcia, Valerie Uribe, Carolyn Hendrickson, Christen Barua, M. Margaret Knudson, John Park, Deanna Lee, Viet Nguyen, India Shelley, Christopher Lee, Marcela Matheus

Idi-Paz-FIBHULP: Jose Lopez-Sendon (Lead), Paula Prieto, Rocio Prieto, Irene Montero

RAPID: Mary Cushman, Lisa Baumann Kreuziger, Shannon Broaddrick

IlliNet: Keri S. Kim, John G. Quigley, Neha Atal

© 2023 Berger JS et al. *JAMA Network Open.*

REMAP-CAP: Bryan McVerry (Lead), David Huang, Renee Wunderly, Meredith Buxton, Tracey Roberts, Kelsey Linstrum, Amanda McNamara, Rebecca Speyer, Stephanie Montgomery, Rykae Cooper
StrokeNet: Hooman Kamel, Pooja Khatri, Jamey Frasure, Amy Silken, Kalli Beasley
BCRI: Renato Lopes, Lilian Mazza, Liliane A. T. Arnaldi Seixas, Mayra Akimi Suiama, Daniele de Lima Franco, Ana Paula de Sousa Mesquita, Jéssica Natuline Ianof, Alef Nunes Araujo
Consorzio Futuro in Ricerca (CRF, Italy): Aldo Maggioni, Chiara Manzalini, Stefania Corsi, Gianluca Campo
AVANTI (Mexico): Jorge Escobedo, Andrea Martinez, Pedro Ohara, Douglas Assis

Site Investigators and Research Coordinators

Brazil

Fundação Faculdade Regional De Medicina De São José Do Rio Preto: Lilia Nigro Maia, Nadielly Prado, Osvaldo Silva Junior, Cláudio Jorge, Osana Costa, Mariana Buka, Danielli Frassatto, Paulo Dutra, Larissa da Silva, Nicolly Alessio, Thaise Pontana, Natalia Cordeiro, Thiago Souza, Jaqueline Mastro, Jaqueline Souza, Cassia Pradela, Larissa Moreira, Guizela Pavon, Andreza Rodrigues, Anna Centurione, Joelma Silva, Katia Andreoti, Renan Vectorazzo, Tamires Silva, Juliana Garcia, Ana PaulaDemore Martins

Instituto Dante Pazzanese de Cardiologia: Idelzuita Leandro Laborase, Mayara Cortez Silva, Renata Viana, Nicole dos Santos Gomes da Silva, David de Andrade Nunes, Kelvin Henrique Villalva, Mariane Higa Chincito, Mateus Arantes Trata, Vanessa Puche Salazar, Romario Cismo de Oliveira

Hospital Universitario Sao Francisco de Assis: Murillo de Oliveira Atunes, Jéssica Tavares de Souza, Pedro Henrique Dias Garcia, Mariana Raquel Gonçalves, Lynnie Oberg Arouca, Tibério Augusto Oliveira Costa, Waleska de Brito Antunes

Hospital São Lucas da PUCRS - União Brasileira de Educação e Assistência: Paulo Ricardo Avancini Caramori, Natalia Lamas Bueno, Denise Machado de Oliveira Pellegrini, Otavio Fachinetto Casagrande, Andrielle Dias Pinheiro

Instituto do Coração do Hospital das Clínicas da Faculdade de Medicina da USP-InCor-HCFMUSP: Alexandre de Matos Soeiro, Tatiana de Carvalho Andreuci Torres, Juliana Brandão de Oliveira

Hospital Universitário de Canoas: cristiano Perdeneiras Jaeger, Evelin Cenci, Thainara Oliveira, Helder Amorim

Italy

Università di Ferrara: Marco Contoli, Alberto Papi, Luca Ronzoni, Gianluca Campo, Chiara Manzalini, Martina Viola
Policlinico di Napoli, Napoli: Nicola Coppola, Caterina Monari, Clarissa Camaioni, Laura Occhiello, Mariaantonietta Pisaturo, Antonio Russo, Margherita Macera, Stefania De Pascalis

ASST Santi Paolo e Carlo, Milano: Antonella dArminio Monforte, Federica Miraglia, Diletta Barbanotti, Matteo Sala, Daniele Tesoro

AOU Sassari, Sassari: Sergio Babudieri, Elija Princic, Ivana Maida, Cosimo Di Castri

Mexico

Hospital de Infectologia Centro Medico Nacional La Raza: Eduardo Mateos, Oswaldo Medina, Paulina Carreno, Yessica Sara Perez, Miguel Angel Cortes, Oswaldo Medina

IMSS Regional General Hospital 196: Luis Rey Garcia Cortes, Blanca Estela Herrera Morales, Juan Lara Cruz, Beatriz Villegas Lara, Oswaldo Medina

Spain

Hospital Universitario Ramón y Cajal: Jose Luis Lopez-Sendón Moreno, Fernando Rodríguez Jorge, Sebastián García Madrona, Raquel Morillo Guerrero, Macarena Alpanes Buesa, Rosa Nieto Royo, Raquel Besse Diaz, Sergio Diz Fariña, Andrés Gonzalez Garcia, Almudena Molinera, Vicente Gómez Olmedo, Paula Gonzalez Ferrandiz, Fernando Hidalgo Salinas, Otilia Navarro, Itziar Pozuelo, Svetlana Zhilina Zhilina

© 2023 Berger JS et al. *JAMA Network Open.*

Hospital Universitari Arnau de Vilanova: Fernando Worner, José Luis Morales, Pablo Pastor, Cristina Acosta, Cristina Marzo, Laura Barta, Blanca Navarro, Núria Miranda

Hospital Universitario La Paz: Esteban López de Sá, Ainara Albaladejo, Ester Zamarrón, Carlos Carpio, Rodolfo Alvarez-Sala, Isabel MaríaFernández Navarro, Guiomar Mediavilla, Luis Gómez, Eduardo Armada, Juan Caro, Sandra Rosillo, Ana Pérez Chica

Hospital Universitario de Salamanca: Miguel Marcos Martin, Amparo López-Bernús, Monica Sanchez, Juan Hernandez, Felipe Alvarez Navia, Moncef Belhassen Garcia, Cristina Carbonell Muñoz, Guillermo Hernandez Perez, Jose Angel Martin Oterino

Hospital Clínico Universitario de Santiago de Compostela: Jose Ramon Gonzalez Juanatey, Jose Seijas, Maria Jesus Dominguez Santallas, Antonio Pose Reino, Luis Valdes Cuadrado, Nuria Rodriguez Nunez, Jose Seijas, Pedro Rascado, Sara Blanco, Alba Mieres

Hospital Universitario A Coruña: Jesus Peteiro, Paula Garcia Gonzalez, Patricia Piñeiro, David Vieito, Rita Galeiras, José María Sánchez-Andrade

Hospital Clinic de Barcelona: Jorge Moises, Jeisson Osorio,

Hospital de Emergencias Enfermera Isabel Zendal: Manuel Alvarez-Gonzalez, Fatima Ibanez-Estellez, Celia Rodríguez-Olleros, Maria Begoña Cabrito Lansorena, Francisco Galiano Cuevas, Sergio Henche Rodriguez, Juliana Perez Hoyos, Maria Isabel Murillo Blasco

United States

NYU Langone Health: Jeffrey Berger, Jonathan D. Newman, Norma Keller, Carlos Alviar, Shari Brosnahan, Eugene Yuriditsky, Tania Ahuja, James Horowitz, Alexander Hindenburg, Karen Veloso, Michael Basseyn, Dany Haddad, Tamta Chkhikvadze, Morris Jrada, Britta Kumley, Tania Ahuja, Aaron Damato, Anthony Passarella, Gustavo Hernandez, Farzana Ahmed, Maja Fadzan, Julia Levine, Michela Garabedian, Shane Francheska Sy, Emily Beato, Sarah Zinn, Gabriela Zapata, Carlos Lopez Jimenez, Alex Auchus

Montefiore Medical Center: Michelle Gong, Daniel Ceusters, Omowunmi Amosu, Hiwet Tzehaie, Rahul Nair, Brenda Lopez, Manuel Hache Marliere, Daniel Fein, Obiageli Offor, , Benjamin Galen, Aram Hambardzumyan, Aditi Desai, Mahmuda Akhter, , Sahil Virdi, Roshni Shah, , Jen-Ting Chen, Amira Mohamed William Nkemdirim, Sabah Boujid, Peter Nauka, Parth Pancholi, Elise Ruan, Ari Moskowitz*Duke University Hospital*: Lana Wahid, Oluwayemisi Mohammed, Emily Ko, Valerie Renard, Tara Strigo Smith, Cameron Wolfe, Rowena Dolor, Thomas Ortel, Molly Daughety, Bryan Feger, Stephen Gazda, Maria Manson, Michael Musty, Amber Daye, Sally Taylor, Lorraine Vergara, Jenny Excelbierd, Ryan Jackson, Gloria Pinero, Barbara Bussadori,

Washington University School of Medicine, ACCS Research: Grant Bochicchio, Kelly Bochicchio, Stacey Reese, Ricardo Fonseca, Bryan Sato, Chris Machica, Jennifer McCarthy, Jose Aldana, Rohit Rasane, Melissa Canas, Hussain Afzal,

Rutgers New Jersey Medical School: Yonatan Greenstein, Randall (Randy) Teeter, Michael Plump, Olga Kovalenko, Eliana Obando, Yanille Taveras, Brittany Fanka, Nipun Suri, Sunil Patel, Maninderpal Kaur, Hala Boktor

OSF Little Company of Mary Medical Center (OSF LCM): M. Bassel Atassi, Shamila Garg, Patricia Mayer, James Vrame

University of Michigan: Robert Hyzy, Pauline Park, Jose Victor Jimenez, Jakob McSparron, Bonnie Wang, Kristine Nelson, Norman Olbrich

Wayne State University: Robert Sherwin, James Wooden, Thomas Mazzocco, Theodore Falcon, Samuel Ceckowski, Sarah Meram, Lauren Buck, Jessica Shuck, Abe Lovelace, Justin Sabol, Jeffery Harrison, Farhan Ayaz, Jacob Winkel

University of Illinois at Chicago Health: John G. Quigley, Keri S. Kim, Jeffrey Jacobson, Neha Atal

© 2023 Berger JS et al. *JAMA Network Open.*

Zuckerberg San Francisco General Hospital Lucy Kornblith, Carolyn Hendrickson, Aaron Kornblith, India Shelley, Deanna Lee, Viet Nguyen, Marcella Matheus, Christopher Lee, Brenda Nunez-Garcia, John Park,

Cleveland Clinic Foundation: Abhijit Duggal, Simon Mucha, Omar Mehkri, Kiran Ashok, Nirosshan Thiruchelvam, Matthew Siuba, Steven Minear, Jinesh Mehta, Carla McWilliams, Chinwe Anekwe, Amy Van, Andrea Calderon, Camila De Carvalho Teixeira, Delmy Zelaya

Oregon Health and Science University: Akram Khan, Olivia Krol, Kinjal Mistry, Kelly Nguyen, Zhengchun Lu, Milad Karami Jouzestani, Atinderpal Singh, Madeline Mcdougal, Andrew Salar, Simeon Florea, Raya Adi, Chandni Anadkat, Emmanuel Mills, Zachary Zouyed, Rupali Deshmukh, Catherine Hough, Minn Oh

SUNY Upstate University Hospital: Julius Gene Latorre, Lena Deb, Sigiriya Smolen, Markus Gutsche, Elena Schmidt, Fadar Otite, Timothy Beutler, Housam Hegazy, Birendra Sah, Zachary Shepherd, Kathryn Anderson, Tanya George, Sanchit Panda, Zaher Oueida, Ioana Amzuta, Dragos Manta, Hayas Haseer Koya, Jivan Lamichhane, Anupa Mandava

University of Alabama: Sheetal Gandotra, Mark Dransfield, Elizabeth Westfall, Micah Whitson, Donna Harris, Derek Russell, Siddharth Patel, Micah Whitson, Jamie Bird, Sonya Hardy, Cynthia Kirkesy

The University of Texas at Austin: Hemali Patel, Kristina Adrean, William Brode, Johanna Busch, Liwayway Andrade, Kristin Mondy, Saurin Gandhi, Kevin Ferriter, Emily Hodge

Temple University: Michael Bromberg, Linda Ayres, P Sheril George, Melissa Navarro, Shubhra Srivastava-Malhotra, Nadia Ali, Bolla Sudhir, Ashwin Chandar, Wissam Chatila, Junad Chowdry, Gerard Criner, Melinda Darnell, Sean Duffy, Jonathan Galli, Jamie Garfield, Rohit Gupta, Fredric Jaffe, Keith Johnson, Navjot Kaur, Victor Kim, Joseph Lambert, Chenna Mandapati, Nathaniel Marchetti, Janpreet Mokha, Catherine Myers, Erin Narewski, Osin O'Corragain, Parag Desai, Maulin Patel, Ekamjeet Randhawa, Daniel Sacher, Daniel Salerno, Kartik Shenoy, Jeffrey Stewart, John Travaline, Maria Vega Sanchez, Matthew Zheng, Julie Juhas, Jenna Murray-Kasznel, Milton DeMaria, Julie O'Connor

University of Wisconsin Hospital; Meriter Hospital: John Sheehan, Sarah Stewart, Kraig Kumfer, Rafael Veintimilla, Chris Roginski, Nicole Bonk, Scott Ensminger, Muhammad Shahzeb Munir, Jashan Octain, Ann Sheehy, Alexis Waters, Scott Wilson

Jacobi Medical Center: Eleonora Gashi, Cidney Schultz, Sabahete Zeqiraj, Seth Sokol, Anjali Acharya, Rosy Thachil, Aline Baday, Kelli Roe, Sindy Mora, Olga Diaz, Jennifer Cho, Heesun Huh

University of Pittsburgh: David Huang, Bryan McVerry, Kelsey Linstrum, Nikki Bensen, Dylan Burbee, Aaron Richardson, Amanda McNamara, Dara Stavor, Menna Abaye, Denise Scholl, Renee Wunderley, Anne Yang, Sher Shah Amin, Emily Berryman, Matthew Gilliam, Carrie Pidro, Michael Muir, Kelly Urbanek

UPMC Presbyterian: David Huang, Bryan McVerry, Nikki Bensen, Dylan Burbee, Aaron Richardson, Amanda McNamara, Dara Stavor, Menna Abaye, Denise Scholl, Renee Wunderley, Anne Yang, Sher Shah Amin, Emily Berryman, Matthew Gilliam**,** Kim Basile, Giles Clermont, William Garrard, Christopher Horvat, Kyle Kalchthaler, Andrew J. King, Daniel Ricketts, Salim Malakouti, Oscar Marroquin, Edvin Music, Kevin Quinn, Mark Andreae, William Bain, Ian Barbash, Emily Brant, David Barton, Meghan Fitzpatrick, Christopher A Franz, Ghady Haidar, Mahwish Hussain, Georgios D Kitsios, Florian B Mayr, Brian Malley, Erin McCreary, Kaveh Moghbeli, Brian Rosborough, Andrew Schoenling, Faraaz A Shah, Varun U Shetty, Tomeka Suber, Nadine Talia, Alexandra Weissman, Caitlin Schaefer, Michael Muir, Kelly Lynn Urbanek

Kansas University Medical Center: Lewis Satterwhite, Penelope Harris, Kimberly Lovell, Mohamed Mourad, Charles Bengtson, Tahani Atieh, Kyle Brownback, Carolina Aguiar, Megan White, Karisa Deculus, Lawrence Scott, Lindsey English, Stephanie Greer, Sharon Murry, Lisa Woodring, Usman Nazir, Amanda Truong, Nelda Mallett, Shereesa Williams, Heidi Hellwig, Michael Burton

Penn State Health Milton S. Hershey Medical Center: Steven Moore, Kunal Karamchandani, Pauline Go, Anthony Bonavia, Lonnie Fender, Nancy Campbell, Judie Howrylak, Kevin Gardner, Lisa Fox, Paula Trump, Katie Loffredo, McKenna Snyder, Sharon O`Brien, Lisa Schultz, Shane Kinard

University of Mississippi Medical Center: Matthew Kutcher, James Galbraith, Alan Jones, Utsav Nandi, Vishnu Garla, Rebekah Peacock, Emily Grenn, Savannah Vann, Alexander Kile, Simon Barinas, Lucia Solis

© 2023 Berger JS et al. *JAMA Network Open.*

Queens Medical Center: Stephanie Guo, Beth Blackburn, Todd Seto, Chris Fiack, Brent Tatsuno, May Vawer, Kuo-Chiang Lian, Michael Yee, Richmond Wong, Ynhu Le

MetroHealth System: Vidya Krishnan, Cindy Newman, Pete Leo, Carla Greenwood, Andrew Wright, Edward L. Warren, John Daryl Thornton, Calen Frolkis, Brianna Arko, Gloria McPhee, Tyler Pope

Ascension St. John Clinical Research Institute: Nicholas Hanna, Anuj Malik, Stacie Merritt, Julie Davenport, Kathryn Mears, Jane Bryce, Melanie Arnold, Joy Norwood, Cheryl Urias, Anna Bryan, Susan Mason, Benjamin Rains, Holly Wall

Westchester Medical Center: Stephen Pan, FNU Namrata, Atul Bali, Avisha Shah, Urvashi Hooda, Matthew Seplowe, Serena Wong, Liana Michaud, Ravi Shah, Gita Blitshteyn, Dennis Boyle, Usman Khan, Anthony Maddalena, Rajan Singh

Medical City Ft Worth: Ranjit Nair, Jing Lu, Bhagawathy Sarma, Brenda Tapia

UCSF San Francisco: Michael Matthay, Kirsten, Kangelaris, Hanjing Zhuo, Kimberly Yee, Alejandra Jauregui, Suzanna Chak, Kimia Ashktorab

Cook County Health: Saurabh Malhotra, Arlet Nedeltcheva, Katayoun Rezai, Michael Hoffman, Ruben Hernandez Acosta, Juan Sarmiento, Shreeyala Uday

Emory: Manila Gaddh, Kim Nguyen, Anna Von, Jason Lucas, Stephanie Whitten, Ananya Hooda, Florence Nwokeji, Pamela Hyde, Aaron Gluth

Kaiser Permanente Fontana: Conrad Liang, Catherine Lui, Vanessa Audea, Marissa Barron, Ashima Sharma, Mazen Noufal, Valerie Wyman

Kaiser Permanente Los Angeles: Zahra Ajani, Catherine Lui, Vanessa Audea, Marissa Barron, Ashima Sharma, Parisa Azizad-Pinto, Yervand Chakryan, Nathalie Sanchez, Monica Guerrero

University of Cincinnati Medical Center: Kristin Hudock, R. Duncan Hite, Harshada More, Hammad Tanzeem, Neil Ernst, Dalton Kuebel

UC San Diego Hillcrest: Todd Costantini, Terry Curry, Allison Berndtson

Ronald Reagan UCLA Medical Center: George Lim, Gregory Hendey, Steven Chang, Nida Qadir, Rebecca Beutler, Trisha Agarwal, Julia Vargas, Anna Yap, Dong Han Yao, Santano Rosario, Anna Jackanich, Greg Kojayan, Austin Wu, William Scheving, Torrence Tran, Michael Lin, Julia Vargas, Hena Sihota,

Stanford University Medical Center: Jennifer Wilson, Angela Rogers, Joseph Levitt, Rosemary Vojnik, Cynthia Perez

Hennepin County Medical Center: Matthew Prekker, Michael Puskarich, Brian Driver, Jason Baker, Adam Kolb, Audrey Hendrickson, Ellen Maruggi, Tayne Andersen, William Miller, Radhika Edpuganti, Quinn Ehlen, Grace Leland, Walker Tordsen, Ann Isaksen, Heidi Erickson

Ochsner Clinic Foundation: Mark Effron, Hunter McDaniel, Sarah Cohen, Derek Vonderhaar, Kristen Rogers, Shane Broussard, Hunter McDaniel, Monique Pellegrin, Nilmo Hernandez

University of Arizona: Bellal Joseph, Afshin Sam, Andrea Seach, Heidi Erickson, Cathleen Wilson, Alejandro Cruz, Breanna Featherston, Iliana Romero, Robin Carlson, Isaac Zarif

Hospital of the University of Pennsylvania: Allyson Pishko, Rolando Vega, Steve Pugliese, Todd Hecht

University of Massachusetts: Robert Finberg, Alonso Alvaro, Shauneen Valliere, Melissa Adams, Kevin O'Day, Craig Dooley, Jennifer Germain, Alexander Hamel, Mauricio Leitao, Cynthia Rup, Michael Seigers

University of Arkansas for Medical Sciences: Srikanth Vallurupalli, Laura Boudrie, Cynthia Wotkowski, Vernon Sincere Jr., Renee Shaide, K'La Polite, Heather Moody, Dannis Armikarina, Amanda Stapleton, Amanda Daniell, Kimberly Belz, Mindy Caid, Amy Crisp, Vallon Williams, Ravula Sreelakshmi

University of Florida: Nicole Iovine, Marie-Carmelle Elie, Brittney Roth Manning, Socorro Pata, Matthew Shaw, Rebecca Murray, Travis Murphy, Rohit Patel, Anita Rajasekhar, Marc Zumberg, Melissa Lewis, Amanda

© 2023 Berger JS et al. *JAMA Network Open.*

Hill, Dironada Strange, Tori Tran, ChristaCampbell, Ryleigh Cahill, Brittany Venegas, Elida Benitez, Chris Duncan, Torben Becker, Melissa Terry-White, Richard Madolid, Travis Roundtree, Cristina Crizaldo

Morehouse School of Medicine: Melvin Echols; Kelly Mahfood, Sharion Smith, Olusola Gbingie, Phillip Powers

University Medical Center of Southern Nevada: Chowdhury Ahsan, Jennifer Robinson, Robert Bimbi, Luzviminda Alexander, Christopher Bates, Karen Schultz, Sumitabh Singh, Vinayak Subramanian, Sujitha Vasireddy

Rutgers Robert Wood Johnson Medical School: John Kostis, Nora Cosgrove, William Kostis, Abel Moreyra, Ami Patel, Fei Chen, Logan Cooney, Deborah McCloskey, Dhruti Patel, Olga Kovalenko, Yonatan Greenstein, Eliana Obando, Brittany Fanka, Sunil Patel

VA New York Harbo+C40r Healthcare System: Binita Shah, Leandro Maranan, Alana Choy-Shan, Nathaniel Smilowitz, Robert Donnino, Jeffrey Lorin, Mary Keary, Thomas Shirley

Geisinger Research: Paul Simonelli, Parth Rao, Kristen Forster, Karen Coleman, Michelle Kopfinger, Kayleighia Miller, Prianka Bhattacharya, Joseph Lynch, Priyanka Pathak, Joseph Vadakara, Cathy Miller, Pamela Borek, Mina Makary, Jasmine Martin, Daniel Carlson, Yvonne Dansoa, Anna Griffith, Delnaz Bakht, Anastasia Schuldt, Kristin Oliver,

Baystate Medical Center: Jay Steingrub, Mark Tidswell, Cynthia Kardos, Lesley De Souza, Scott Ouellette, , Sarah Romain, Rae Lynn DeFeo, , Denise Gallant,

University Medical Center-LSU: Matthew Lammi, Bennett deBoisblanc, Kyle Happel, Amita Krishnan, Shane Sanne, Connie Romaine, Paula Lauto, Marie Sandi

Ohio State Universtiy Wexner Medical Center: Matthew Exline, Joshua Englert, Sonal Pannu, Emily Robart, Sarah Karow, Joseph Bednash, Mohammad Sobhanie, Elizabeth Schwartz, Preston So, Madison So

Swedish Hospital: Shane O'Mahony, Julie Wallick, Stephanie Johnson, Julia Karr, Paula Manner, David, Wilson, John Kaneko, Heather Algren, Rebecca Cochran, Alexandria Duven, Dakota Fletcher, Joshua Mark

Lundquist Institute for Biomedical Innovation at Harbor-UCLA Medical Center: Janine Vintch, William Stringer, Leticia Diaz, Agustin Leyva, Indelicato, David Yuchno, Robert Gruhn

Cooper Health: Nitin Puri, Lynette Harris, Christa Schorr, Adam Green, Jason Bartock, Christopher Jones, Phil Dellinger, Shawana Hussain, Naitik Patel, Lianne Kikia-Yang

Henry Ford Medical Center: Scott Kaatz, Stacy Ellsworth, Jacqueline Day, Sheri Renaud, Vinay Shah, Sara Hegab, Beverly Stallings, Bethany Adams

Beaumont Medical Center: Girish Nair, Tammy Osentoski, Sanjay Dogra, Coleen Tessmar

Newark Beth Israel Medical Center: Alice Cohen, Mitali Pradhan, Cynthia Horta, Gabriella Tedesco, Sari Jacoby, Maya Shah, Arjun Gadhiya

University of Vermont: Christos Colovos, Ethan Jones, Mohit Jindal, Roz King, Skyler Lentz, Adam Ackerman

Wake Forest: Ashish Khanna, Lynette Harris, Brandon Reeves, Bruce Cusson, Ryan Maves, Leonard Stallings, Mhorys Pickmans, Kinchit Shah, Hinna Wadhwani, Jessica Reeves, Tanmay Sura, Amelia Eaton

Doctors Medical Center of Modesto, Inc.: James Jaffe, Dorothy Trivedi

Spectrum Health (Butterworth Hospital): Muhib Khan, Heidi Taylor, Leo Gallagher, Brian MacDougall, Heather Benningfield, Beth Carrier, Katie Dykstra, Sasha Richardson, Katherine Root, Marianne Morrissey, Malik Khan

Indiana University Health Methodist Hospital: Rajat Kapoor, Melissa Astin, Damien Patel, Omar Rahman, Meghan Willig, Shawna Prange, Heather Adams, Jean Nash, Terri Strickland, Kimberly McPeak, Srdjan Kurbalija, Anna Smith, Molly Stearns, Dave Estelle, Chelsea McCrory

© 2023 Berger JS et al. *JAMA Network Open.*

eAppendix 2 – Supplemental Methods

Endpoint Definitions

The full list of secondary endpoints is available in the trial protocol. The CEC will consider for adjudication all cases of the following:

- Deep venous thromboembolism
- Pulmonary embolism
- Arterial thromboembolism
- Myocardial infarction
- Stroke
- Major bleeding
- Death due to cardiovascular, non-cardiovascular, and undetermined cause

Deep Venous Thromboembolism

The diagnosis of definite symptomatic deep venous thromboembolism (DVT) requires symptoms of venous thromboembolism with at least one of the following:

- Abnormal compression ultrasound consistent with DVT or abnormal flow pattern or direct clot visualization in veins not amenable to compression.
- One or more new filling defects by venography, CT venography, or MR venography.
- Abnormal compression ultrasound where compression had been normal or, if known to be non-compressible, a substantial increase (≥4mm) in the diameter of a previously non-compressible venous segment.
- Point-of-care ultrasound (POCUS) performed by a provider and documenting DVT in a note.
- An extension of an intraluminal filling defect, or a new intraluminal filling defect, or an extension of non-visualization of veins in the presence of a sudden cut-off on venography.
- Proximal DVT is defined as clot at or proximal to the trifurcation of the popliteal vein (in the lower extremity) OR clot at or proximal to the axillary vein segment (in the upper extremity).
- Distal DVT is defined as clot distal to the trifurcation of the popliteal vein (in the lower extremities) OR clot at or distal to the brachial vein segment (in the upper extremities).
- Non-limb venous thrombosis includes thrombosis of the cerebral, portal, mesenteric, hepatic, gonadal, splenic, renal, or retinal veins, or thrombosis of the superior or inferior vena cava.

The diagnosis of presumed deep venous thromboembolism requires the following:

- In the absence of objective testing, high pre-test probability according to investigator assessment
  - OR adjudicator's gestalt
  - OR Wells score f
- AND a treatment plan for DVT was initiated (initiation of anticoagulation, or escalation of anticoagulation dose, frequency, or duration).

Pulmonary Embolism

The diagnosis of definite pulmonary embolism requires at least one of the following:

- New intraluminal filling defect at CT pulmonary angiography in a subsegmental or larger vessel.
- New intraluminal filling defect, or an extension of an existing defect, or a new sudden cut-off of vessels > 2.5 mm in diameter at pulmonary angiogram
- Inconclusive CT pulmonary angiography, pulmonary angiography, or VQ scan evidence of a new or recurrent PE with demonstration of a new or recurrent DVT in the lower extremities by compression ultrasonography or venography.[4,5]
- New clot or intraluminal filling defect noted in the right heart ("clot in transit") or the pulmonary vasculature at echocardiogram
- High probability (revised PIOPED criteria) on planar ventilation/perfusion (V/Q) scan OR positive PE on SPECT ventilation perfusion (V/Q) scan.
- Pulmonary embolism found at autopsy

© 2023 Berger JS et al. *JAMA Network Open.*

The diagnosis of presumed pulmonary embolism requires the following:

Clinical signs and symptoms of pulmonary embolism, including but not limited to dyspnea, cough, hypoxemia, tachycardia, appropriate electrocardiographic changes, or evidence of right heart strain on echocardiogram; AND chest CT or pulmonary angiography are unable to be performed AND therapeutic dose anticoagulation or fibrinolytic therapy is prescribed by a physician

Arterial Thromboembolism

The diagnosis of arterial thromboembolism is defined as the following:

- A clinical history and presentation consistent with a sudden significant worsening of end organ or limb perfusion AND

EITHER

- Confirmation of arterial obstruction by imaging, hemodynamics, intraoperative findings, or pathological evaluation

OR

- Requirement for thrombolysis, thrombectomy, or urgent bypass.

Note that arterial thromboembolism includes both acute *in situ* thrombotic events and acute embolic events. Note that while ischemic stroke and myocardial infarction can be arterial thromboembolic events, those events will be adjudicated according to the separate standardized criteria included below.

Myocardial Infarction

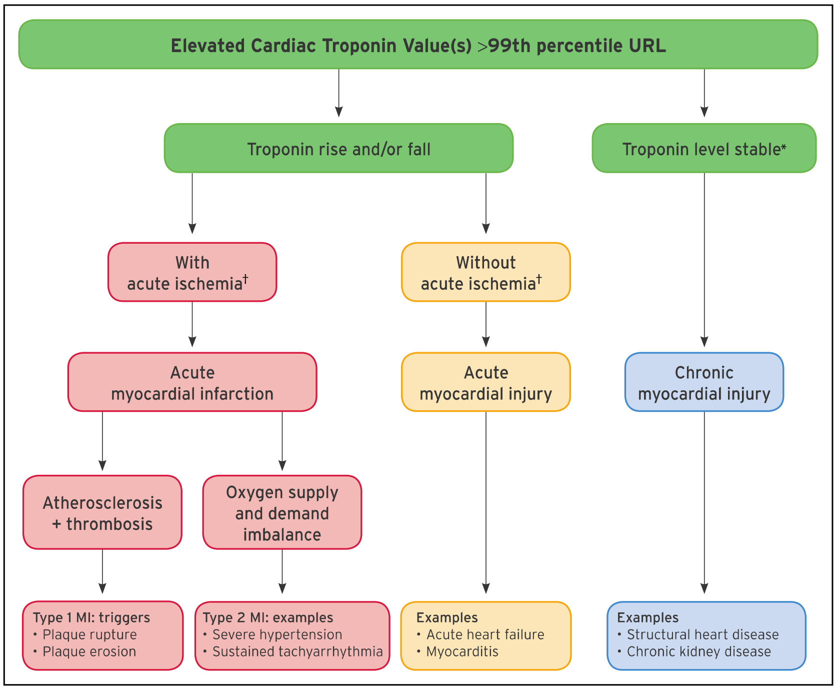

Figure 1. Model for interpreting myocardial injury.[1]

COVID-19 patients are well known to have elevations in cardiac troponin concentrations, and these elevations often do not represent arterial thrombosis and downstream myocardial ischemia. Therefore, the CEC will make an effort to distinguish true myocardial infarction from coronary artery obstruction, typically from atherothrombosis (usually considered a "type 1 myocardial infarction") from myocardial infarction due to demand ischemia (usually defined as a "type 2 myocardial infarction") and myocardial injury (an elevation in cardiac troponin typically without symptoms of chest pain or signs of arterial thrombosis). These definitions will be consistent with the 4th Universal Definition of Myocardial Infarction and will take into considerations suggestions made about classification of certain conditions as type 1 as compared to type 2 myocardial infarction. Regional coronary venous thrombosis with associated regional myocardial infarction has been reported in COVID. If this mechanism is documented, these will be considered a type 1 MI. The trial and CEC are focused on ascertaining and

© 2023 Berger JS et al. *JAMA Network Open.*

adjudicating cases of acute myocardial injury and acute myocardial infarction and classifying those cases as described below. COVID also causes microvascular thrombi which are associated with patchy myocardial necrosis. These will be grouped with myocardial injury.

2. UNIVERSAL DEFINITIONS OF MYOCARDIAL INJURY AND MYOCARDIAL INFARCTION: SUMMARY

Universal definitions of myocardial injury and myocardial infarction

Criteria for myocardial injury

The term myocardial injury should be used when there is evidence of elevated cardiac troponin values (cTn) with at least 1 value above the 99th percentile upper reference limit (URL). The myocardial injury is considered acute if there is a rise and/or fall of cTn values.

Criteria for acute myocardial infarction (types 1, 2 and 3 MI)

The term acute myocardial infarction should be used when there is acute myocardial injury with clinical evidence of acute myocardial ischemia and with detection of a rise and/or fall of cTn values with at least 1 value above the 99th percentile URL and at least 1 of the following:

- Symptoms of myocardial ischemia;
- New ischemic ECG changes;
- Development of pathological Q waves;
- Imaging evidence of new loss of viable myocardium or new regional wall motion abnormality in a pattern consistent with an ischemic etiology;
- Identification of a coronary thrombus by angiography or autopsy (not for types 2 or 3 MIs).

Postmortem demonstration of acute atherothrombosis in the artery supplying the infarcted myocardium meets criteria for *type 1 MI*. Evidence of an imbalance between myocardial oxygen supply and demand unrelated to acute atherothrombosis meets criteria for *type 2 MI*. Cardiac death in patients with symptoms suggestive of myocardial ischemia and presumed new ischemic ECG changes before cTn values become available or abnormal meets criteria for *type 3 MI*.

Criteria for coronary procedure–related myocardial infarction (types 4 and 5 MI)

Percutaneous coronary intervention (PCI)–related MI is termed *type 4a MI*.

Coronary artery bypass grafting (CABG)–related MI is termed *type 5 MI*.

Coronary procedure–related MI ≤48 hours after the index procedure is arbitrarily defined by an elevation of cTn values >5 times for *type 4a MI* and >10 times for *type 5 MI* of the 99th percentile URL in patients with normal baseline values. Patients with elevated preprocedural cTn values, in whom the preprocedural cTn level are stable (≤20% variation) or falling, must meet the criteria for a >5 or >10 fold increase and manifest a change from the baseline value of >20%. In addition with at least 1 of the following:

- New ischemic ECG changes (this criterion is related to *type 4a MI* only);
- Development of new pathological Q waves;
- Imaging evidence of loss of viable myocardium that is presumed to be new and in a pattern consistent with an ischemic etiology;
- Angiographic findings consistent with a procedural flow-limiting complication such as coronary dissection, occlusion of a major epicardial artery or graft, side-branch occlusion-thrombus, disruption of collateral flow or distal embolization.

Isolated development of new pathological Q waves meets the *type 4a MI* or *type 5 MI* criteria with either revascularization procedure if cTn values are elevated and rising but less than the prespecified thresholds for PCI and CABG.

Other types of 4 MI include *type 4b MI* stent thrombosis and *type 4c MI* restenosis that both meet *type 1 MI* criteria.

Postmortem demonstration of a procedure-related thrombus meets the *type 4a MI* criteria or *type 4b MI* criteria if associated with a stent.

Criteria for prior or silent/unrecognized myocardial infarction

Any 1 of the following criteria meets the diagnosis for prior or silent/unrecognized MI:

- Abnormal Q waves with or without symptoms in the absence of nonischemic causes.
- Imaging evidence of loss of viable myocardium in a pattern consistent with ischemic etiology.
- Patho-anatomical findings of a prior MI.

CABG indicates coronary artery bypass grafting; cTn, cardiac troponin; ECG, electrocardiogram; MI, myocardial infarction; PCI, percutaneous coronary intervention; URL, upper reference limit.

Figure 2. Table from the 4th Universal Definition of Myocardial Infarction summarizing the different definitions of myocardial injury and infarction.[1]

© 2023 Berger JS et al. *JAMA Network Open.*

Myocardial Injury: The increasing sensitivity of cardiac troponin (cTn) assays means that ongoing myocardial injury is frequently detected. Myocardial injury is a prerequisite for myocardial infarction (MI), but as noted below, criteria in addition to myocardial injury are necessary to make the diagnosis of MI. Adjudicators must distinguish between acute myocardial injury that is not secondary to ischemia but may be due to other conditions (Table 1).

Table 2. Causes of non-ischemic myocardial injury. [2,3]

Heart failure	Sepsis, infectious disease
Myocarditis	Chronic kidney disease
Cardiomyopathy	Stroke, subarachnoid hemorrhage
Takotsubo syndrome	Pulmonary embolism, pulmonary hypertension
Coronary revascularization procedure	Infiltrative disease, e.g., amyloidosis, sarcoidosis
Cardiac procedure other than revascularization	Chemotherapeutic agents
Catheter ablation	Critically ill patients
Defibrillator shocks	Strenuous exercise
Cardiac contusion	Other

<u>Criteria for Myocardial Injury:</u> Detection of an elevated cTn value above the 99th percentile upper reference limit (URL) is defined as myocardial injury. The injury is considered acute if there is a rise and/or fall of cTn values.[1]

<u>Criteria for Procedure Related Myocardial Injury:</u> Cardiac procedural myocardial injury is arbitrary defined by increased in cTn values (>99th percentile URL) in patients with normal baseline values (<99th percentile URL) or a rise of cTn values >20% of the baseline value when it is the above the 99th percentile URL but is stable or falling.

Myocardial Infarction Type 1: Detection of rise and/or fall of cardiac biomarkers with at least one value above the 99th percentile of the upper reference limit (URL) together with evidence of myocardial ischemia with at least one of the following:

- Symptoms of ischemia
- New ischemic ECG changes indicative of new ischemia (new ST-T changes or new LBBB) *
- Development of pathological Q waves in the ECG**
- Imaging evidence of new loss of viable myocardium or new regional wall motion abnormality in a pattern consistent with an ischemic etiology
- Identification of a coronary thrombus by angiography including intracoronary imaging or by autopsy†
- *ECG manifestation of acute myocardial ischemia (in the absence of LVH and LBBB):
    - ST Elevation: New ST elevation at the J-point in two contiguous leads with the cut-point: ≥ 1 mm in all leads other than leads V2-V3, where the following cut-points apply: ≥2mm in men ≥40 years; ≥2.5 mm in men <40 years; or ≥ 1.5 mm in women regardless of age.
    - ST-depression and T-wave changes: New horizontal or down-sloping ST depression ≥ 0.5 mm in 2 contiguous leads and/or T inversion ≥ 1 mm in two contiguous leads with prominent R waves or R/S ratio >1.
- **Pathological Q waves:
    - Any Q-wave in leads V2-V3 >0.02 seconds or QS complex in leads V2-V3
    - Q-wave ≥ 0.03 seconds and ≥1 mm deep or QS complex in leads I, II, aVL, aVF, or V4-V6 in any 2 leads of a contiguous lead grouping (I, aVL;V1-V6; II, III, aVF; V7-V9).
    - R-wave ≥ 0.04s in V1–V2 and R/S ≥ 1 with a concordant positive T-wave in the absence of a conduction defect
- †Postmortem demonstration of an atherothrombosis in the artery supplying the infarcted myocardium, or a macroscopically large, circumscribed area of necrosis with or without intramyocardial hemorrhage meets the type 1 MI criteria regardless of cTn values.
- Consideration will be given to recent proposals to modify myocardial infarction type 1 to include coronary obstruction by spontaneous coronary artery dissection, coronary embolism, or coronary vasospasm or microvascular dysfunction.[5]

© 2023 Berger JS et al. *JAMA Network Open.*

Myocardial Infarction Type 2: Detection of a rise and/or fall of cTn values with at least 1 value above the 99th percentile URL, and evidence of imbalance between myocardial oxygen supply and demand unrelated to coronary atherothrombosis, requiring at least 1 of the following:

- Symptoms of acute myocardial ischemia
- New ischemic ECG changes
- Development of pathological Q waves;
- Imaging evidence of new loss of viable myocardium or new regional wall motion abnormality in a pattern consistent with ischemic etiology

Myocardial Infarction Type 3: Patients who suffer cardiac death, with symptoms suggestive of myocardial ischemia accompanied by presumed new ischemic ECG changes or ventricular fibrillation but die before blood samples for biomarkers can be obtained, or before increases in cardiac biomarkers can be identified, or MI is detected by autopsy examination.

Myocardial infarction Type 4a and 4b (myocardial infarction associated with percutaneous coronary intervention): Criteria for percutaneous coronary intervention (PCI)-related MI ≤48 hours after the index procedure are as follows: Coronary intervention-related MI is arbitrarily defined by an elevation of cTn values >5 times the 99the percentile URL in patients with normal baseline values. In patients with elevated preprocedural cTn in whom the cTn levels are stable (≤20% variation) or falling, the post procedure cTn must rise by >20%. However, the absolute procedural value must still be at least 5 times the 99th percentile URL. In addition, 1 of the following elements is required:

- New ischemic ECG changes
- Development of new pathological Q waves; note that the development of new pathological Q waves meets the criteria for procedure-related MI if the cTn values are elevated and rising but <5 times the 99th percentile URL.
- Imaging evidence of new loss of viable myocardium or new regional wall motion abnormality in a pattern consistent with an ischemic etiology
- Angiographic findings consistent with a procedural flow-limiting complication such as coronary dissection, occlusion of a major epicardial artery or side branch occlusion/thrombus, disruption of collateral flow, or distal embolization.
- Type 4a MI is an MI associated with PCI
- Type 4b MI is an MI associated with stent/scaffold thrombosis

Myocardial Infarction Type 4c: A type 4c MI is an MI associated with restenosis associated with prior PCI. Possible Type 4c MI is evaluated using the same criteria as Type 1 MI.

Myocardial Infarction Type 5: Criteria of coronary artery bypass grafting (CABG)-related MI ≤ 48 hours after the index procedure. CABG-related MI is arbitrarily defined as elevation of cTn values >10 times the 99th percentile URL in patients with normal baseline cTn values. In patients with elevated preprocedural cTn in whom cTn are stale (≤20% variation) or falling, the post procedure cTn must rise by >20%. However, the absolute postprocedural values must still be >10 times the 99th percentile URL. In addition, one of the following elements is required:

- Development of new pathological Q waves; note that the development of new pathological Q waves meets the criteria for procedure-related MI if the cTn values are elevated and rising but <10 times the 99th percentile URL.
- Angiographically documented new graft occlusion or new native coronary artery occlusion;
- Imaging evidence of new loss of viable myocardium or new regional wall motion abnormality in a pattern consistent with an ischemic etiology

© 2023 Berger JS et al. *JAMA Network Open.*

Special or unusual circumstances: Further guidance on distinguishing myocardial injury from myocardial infarction in the context of non-cardiac surgery, heart failure, myocarditis, Takotsubo syndrome, kidney disease, and in critically ill patients, and myocardial infarction nonobstructive coronary arteries is included in the 4th Universal Definition of MI.[1]

Stroke

The definition of stroke used here is drawn from the definitions proposed by Hicks et al. and Sacco et al.[6,7] Stroke is defined as the acute onset of focal neurological dysfunction caused by brain, spinal cord, or retinal vascular injury as a result of hemorrhage or infarction.

A stroke is the acute onset of a new persistent neurological deficit attributed to an obstruction in cerebral blood flow with no apparent nonvascular cause (e.g., tumor, trauma, infection). Available neuroimaging studies will be considered to support the clinical impression and to determine if there is a demonstrable lesion compatible with an acute stroke. To the extent possible, all strokes will be classified as ischemic, hemorrhagic, or unknown.

For the diagnosis of stroke, the following criteria should be fulfilled:

1. Rapid onset of a focal neurological deficit not related to any other known non-cerebrovascular process with at least one of the following:
- Change in level of consciousness
- Hemiplegia
- Hemiparesis
- Numbness or sensory loss affecting one side of the body
- Dysphasia/aphasia
- Hemianopia
- Other new neurological sign/symptom(s) consistent with stroke
- If the timing of onset is uncertain, a diagnosis of stroke may be made provided that there are no plausible non-stroke causes for the clinical presentation.

AND

2. Duration of a focal/global neurological deficit that is:
- EITHER ≥ 24 hours,
- OR < 24 hours if:
    - Resolution of symptoms is due to least one of the following interventions:
        1. Pharmacologic: intravenous or intraarterial thrombolysis
        2. Non-pharmacologic: (i.e., neuro-interventional procedure such as intracranial angioplasty)
    - OR available MRI clearly documents a new hemorrhage or infarct
    - OR available head CT clearly documents a new hemorrhage or infarct or excludes a mimic of stroke
    - OR the neurological deficit results in death.

Ideally, at least one of should be present to confirm the diagnosis of stroke:

- Confirmation by neurology or neurosurgery specialist
- Brain imaging procedure (at least one of the following): CT scan, MRI scan, or cerebral vessel angiography
- Lumbar puncture (i.e., spinal fluid analysis diagnostic of intracranial hemorrhage)

If the acute focal signs represent a worsening of a previous deficit, these signs must persist for more than 24 hours and be accompanied by an appropriate new MRI or CT scan finding.

Strokes are sub-classified as follows:

Ischemic (non-hemorrhagic): An acute episode of focal cerebral, spinal, or retinal dysfunction caused by infarction of central nervous system tissue. Hemorrhage may be a consequence of ischemic stroke. In this situation, the stroke is an ischemic stroke with hemorrhagic transformation and not a hemorrhagic stroke but would also be listed as a major bleeding safety event.

© 2023 Berger JS et al. *JAMA Network Open.*

Hemorrhagic: An acute episode of focal or global cerebral or spinal dysfunction caused by intraparenchymal, intraventricular, or subarachnoid hemorrhage. Hemorrhage in the brain is documented by neuroimaging or autopsy or lumbar puncture. Note that subdural hematomas are intracranial hemorrhagic events and not strokes.

Undetermined: An acute episode of focal or global neurological dysfunction caused by presumed brain, spinal cord, or retinal vascular injury as a result of hemorrhage or infarction but with insufficient information to allow categorization as either ischemic or hemorrhagic.

Major Bleeding

Major bleeding is defined as acute clinically overt bleeding associated with one or more of the following (as per ISTH guidelines):[8-10]

Decrease in hemoglobin of 2 g/dL or more;

- Transfusion of 2 units or more of packed red blood cells;
- Bleeding that occurs in at least one of the following critical sites:
    - Intracranial
    - Intraspinal
    - Intraocular (within the corpus of the eye. A conjunctival bleed is not an intraocular bleed)
    - Pericardial
    - Intraarticular
    - Retroperitoneal
    - Intramuscular with compartment syndrome
- Bleeding that leads to death (primary cause of death or contributes directly to death)

eReferences

1. Thygesen K, Alpert JS, Jaffe AS, et al. Fourth Universal Definition of Myocardial Infarction (2018). *Circulation.* 2018;138(20):e618-e651.
2. de Lemos JA, Newby LK, Mills NL. A Proposal for Modest Revision of the Definition of Type 1 and Type 2 Myocardial Infarction. *Circulation.* 2019;140(22):1773-1775.
3. Cuker A, Tseng EK, Nieuwlaat R, et al. American Society of Hematology living guidelines on the use of anticoagulation for thromboprophylaxis in patients with COVID-19: May 2021 update on the use of intermediate intensity anticoagulation in critically ill patients. *Blood Adv.* 2021.
4. Agnelli G, Becattini C, Meyer G, et al. Apixaban for the Treatment of Venous Thromboembolism Associated with Cancer. *N Engl J Med.* 2020;382(17):1599-1607.
5. Investigators E-P, Buller HR, Prins MH, et al. Oral rivaroxaban for the treatment of symptomatic pulmonary embolism. *N Engl J Med.* 2012;366(14):1287-1297.
6. Sacco RL, Kasner SE, Broderick JP, et al. An updated definition of stroke for the 21st century: a statement for healthcare professionals from the American Heart Association/American Stroke Association. *Stroke.* 2013;44(7):2064-2089.
7. Hicks KA, Tcheng JE, Bozkurt B, et al. 2014 ACC/AHA Key Data Elements and Definitions for Cardiovascular Endpoint Events in Clinical Trials: A Report of the American College of Cardiology/American Heart Association Task Force on Clinical Data Standards (Writing Committee to Develop Cardiovascular Endpoints Data Standards). *J Am Coll Cardiol.* 2015;66(4):403-469.
8. Giannitsis E, Katus HA. Cardiac troponin level elevations not related to acute coronary syndromes. *Nat Rev Cardiol.* 2013;10(11):623-634.
9. Schulman S, Kearon C, Subcommittee on Control of Anticoagulation of the S, Standardization Committee of the International Society on T, Haemostasis. Definition of major bleeding in clinical investigations of antihemostatic medicinal products in non-surgical patients. *J Thromb Haemost.* 2005;3(4):692-694.
10. Kaatz S, Ahmad D, Spyropoulos AC, Schulman S, Subcommittee on Control of A. Definition of clinically relevant non-major bleeding in studies of anticoagulants in atrial fibrillation and venous thromboembolic disease in non-surgical patients: communication from the SSC of the ISTH. *J Thromb Haemost.* 2015;13(11):2119-2126.

© 2023 Berger JS et al. *JAMA Network Open.*
