# Supplementary material for: Effect of P2Y12 Inhibitors on Organ Support–Free Survival in Critically Ill Patients Hospitalized for COVID-19: A Randomized Clinical Trial
Source: JAMA Netw Open. 2023 May 25;6(5):e2314428. doi: 10.1001/jamanetworkopen.2023.14428 (PMC10214036; doi:10.1001/jamanetworkopen.2023.14428)
Supplement: Supplement 3. — Nonauthor Collaborators [file jamanetwopen-e2314428-s003.pdf]

Supplemental Online Content: Nonauthor Collaborators
*First name, last name, and suffix (if applicable) are required and will appear in PubMed.

*Group Name(s): ACTIV-4a Investigators							
***First Name and Middle Initial(s)**	***Last Name**	***Suffix (eg, Jr, III)**	Academic Degrees	Institution	Location (city, state/province, country)	Role or Contribution, eg, chair, principal investigator	Group (if more than 1 Group listed in the byline) and/or Subgroup (eg, Steering Committee)
Judith S.	Hochman			NYU Langone Health	New York, NY, USA	Chair	
Matthew D.	Neal			University of Pittsburgh	Pittsburgh, PA, USA	Co-Chair	
Jeffrey S.	Berger			NYU Langone Health	New York, NY, USA	PI	
Mikhail	Kosiborod					PI - SGLT2	
Scott	Solomon					PI - Criza	
Mark	Geraci			University of Pittsburgh	Pittsburgh, PA, USA		
Mary	Cushman			University of Vermont	Burlington, VT, USA		
Scott	Berry			Berry Consultants	Austin, TX, USA		
Michael	Farkouh			University Health Network	Toronto, ON, Canada		
Michelle	Gong			Montefiore Medical Center	Bronx, NY, USA		
Kristin	Hudock			University of Cincinnati	Cincinnati, OH, USA		
Keri S.	Kim			University of Illinois at Chicago	Chicago, IL, USA		
Lucy Z.	Kornblith			University of California, San Francisco	San Francisco, CA, USA		
Patrick R.	Lawler			University Health Network	Toronto, ON, Canada		
Aldo	Maggioni			ANMCO Research Center	Florence, Italy		
Renato	Lopes			Duke Clinical Research Institute	Durham, NC, USA		
Jose	Lopez-Sendon			Hospital Universitario La Paz	Madrid, Spain		
Bridget-Anne	Kirwan			SOCAR Research	Nyon, Vaud, Switzerland		
Hooman	Kamel			Cornell University	Ithaca, NY, USA		
Ewan	Goligher			University Health Network	Toronto, ON, Canada		
Ryan	Zarychanski			Cancer Care Manitoba	Winnipeg, MB, Canada		
Pooja	Khatri			University of Cincinnati	Cincinnati, OH, USA		
Eric	Leifer			NHLBI	Bethesda, MD, USA		
Bryan J.	McVerry			University of Pittsburgh Medical Center	Pittsburgh, PA, USA		
Harmony R.	Reynolds			NYU Langone Health	New York, NY, USA		
Jennifer G.	Wilson			Stanford University	Standford, CA, USA		
Erinn	Hade						

Supplemental Online Content: Nonauthor Collaborators
*First name, last name, and suffix (if applicable) are required and will appear in PubMed.

***First Name and Middle Initial(s)**	***Last Name**	***Suffix (eg, Jr, III)**	Academic Degrees	Institution	Location (city, state/province, country)	Role or Contribution, eg, chair, principal investigator	Group (if more than 1 Group listed in the byline) and/or Subgroup (eg, Steering Committee)
Cheng	Yu						
Charles	Lowenstein						
Orly	Vardeny						
Otavio	Berwanger						
Andrew	Althouse						
Ali	Javaheri						
Hooman Kamel	Kamel						
Aurea	Gagliardotto						
Justine	Eisenberg						
Cheri	Barnette						
Ankeet	Bhatt						
Brenden	Everett			Brigham and Women's Hospital	Boston, MA, USA		
Aira	Contreras			NYU Langone Health	New York, NY, USA		
Stephanie	Mavromichalis			NYU Langone Health	New York, NY, USA		
Eduardo	Iturrate			NYU Langone Health	New York, NY, USA		
Margaret	Gilsenan			NYU Langone Health	New York, NY, USA		
Anna	Naumova			NYU Langone Health	New York, NY, USA		
Arlene	Roberts			NYU Langone Health	New York, NY, USA		
Jonathan	Newman			NYU Langone Health	New York, NY, USA		
Julia	Levine			NYU Langone Health	New York, NY, USA		
Michelle	Chang			NYU Langone Health	New York, NY, USA		
Alair	Holden			NYU Langone Health	New York, NY, USA		
Stephen	Wisniewski			University of Pittsburgh	Pittsburgh, PA, USA	Co-Chair	
Christine	Leeper			University of Pittsburgh	Pittsburgh, PA, USA		
Derek	Angus			University of Pittsburgh	Pittsburgh, PA, USA		
Mary	Martinez			University of Pittsburgh	Pittsburgh, PA, USA		
Jake	Schreiber			University of Pittsburgh	Pittsburgh, PA, USA		
Valena	Lundy-Wiggins			University of Pittsburgh	Pittsburgh, PA, USA		
Joshua	Froess			University of Pittsburgh	Pittsburgh, PA, USA		
Ashita Sai	Vadlamudi			University of Pittsburgh	Pittsburgh, PA, USA		

Supplemental Online Content: Nonauthor Collaborators
*First name, last name, and suffix (if applicable) are required and will appear in PubMed.

***First Name and Middle Initial(s)**	***Last Name**	***Suffix (eg, Jr, III)**	Academic Degrees	Institution	Location (city, state/province, country)	Role or Contribution, eg, chair, principal investigator	Group (if more than 1 Group listed in the byline) and/or Subgroup (eg, Steering Committee)
Frank	Sciurba			University of Pittsburgh	Pittsburgh, PA, USA		
Alison	Morris			University of Pittsburgh	Pittsburgh, PA, USA		
Edvin	Music			University of Pittsburgh	Pittsburgh, PA, USA		
Bridget-Anne	Kirwan			SOCAR Research	Nyon, Vaud, Switzerland		
Sophie	de Brouwer			SOCAR Research	Nyon, Vaud, Switzerland		
Emilie	Perrin			SOCAR Research	Nyon, Vaud, Switzerland		
Caroline	Gombault			SOCAR Research	Nyon, Vaud, Switzerland		
Sandra	Bula			SOCAR Research	Nyon, Vaud, Switzerland		
Michael	Nelson			SOCAR Research	Nyon, Vaud, Switzerland		
Céline	Daelemans			SOCAR Research	Nyon, Vaud, Switzerland		
Laurine	Paraz			SOCAR Research	Nyon, Vaud, Switzerland		
Michelle	Detry			Berry Consultants	Austin, TX, USA		
Anna	McGlothlin			Berry Consultants	Austin, TX, USA		
Melanie	Quintana			Berry Consultants	Austin, TX, USA		
Amy	Crawford			Berry Consultants	Austin, TX, USA		
Elizabeth	Lorenzi			Berry Consultants	Austin, TX, USA		
Lindsay	Berry			Berry Consultants	Austin, TX, USA		
Jocelyn	Chen						
Anna	Heath						
Harmony R.	Reynolds			ISCHEMIA/MINOCA-HARP/EPPIC-NET			
Aira	Contreras			ISCHEMIA/MINOCA-HARP/EPPIC-NET			
Stephanie	Mavromichalis			ISCHEMIA/MINOCA-HARP/EPPIC-NET			
Margaret	Gilsenan			ISCHEMIA/MINOCA-HARP/EPPIC-NET			
Anna	Naumova			ISCHEMIA/MINOCA-HARP/EPPIC-NET			
Danielle	Sin			ISCHEMIA/MINOCA-HARP/EPPIC-NET			

Supplemental Online Content: Nonauthor Collaborators
*First name, last name, and suffix (if applicable) are required and will appear in PubMed.

***First Name and Middle Initial(s)**	***Last Name**	***Suffix (eg, Jr, III)**	Academic Degrees	Institution	Location (city, state/province, country)	Role or Contribution, eg, chair, principal investigator	Group (if more than 1 Group listed in the byline) and/or Subgroup (eg, Steering Committee)
Elhaji	Diene			ISCHEMIA/MINOCA-HARP/EPPIC-NET			
Ewelina	Gwiszcz			ISCHEMIA/MINOCA-HARP/EPPIC-NET			
Isabelle	Hogan			ISCHEMIA/MINOCA-HARP/EPPIC-NET			
Alair	Holden			ISCHEMIA/MINOCA-HARP/EPPIC-NET			
Michelle	Gong			PETAL			
Nancy	Ringwood			PETAL			
Laura	Fitzgerald			PETAL			
Haley	Morin			PETAL			
Lucy	Kornblith			MULTINET			
Brenda	Nunez-Garcia			MULTINET			
Aaron	Kornblith			MULTINET			
Carolyn	Hendrickson			MULTINET			
Deanna	Lee			MULTINET			
Viet	Nguyen			MULTINET			
India	Shelley			MULTINET			
India	Loar			ATTACC/REMAP			
Mary	Cushman			RAPID			
Lisa	Baumann Kreuziger			RAPID			
Stephanie	Jones			RAPID			
Keri S.	Kim		PharmD	ILLINET		Network co-lead	
John G.	Quigley		MD	ILLINET		Network co-lead	
Neha	Atal		MBA, MIIS	ILLINET		Network coordinator	
Bryan	McVerry			REMAP-CAP			
David	Huang			REMAP-CAP			
Renee	Wunderly			REMAP-CAP			
Meredith	Buxton			REMAP-CAP			
Tracey	Roberts			REMAP-CAP			

*First name, last name, and suffix (if applicable) are required and will appear in PubMed.

*First Name and Middle Initial(s)	*Last Name	*Suffix (eg, Jr, III)	Academic Degrees	Institution	Location (city, state/province, country)	Role or Contribution, eg, chair, principal investigator	Group (if more than 1 Group listed in the byline) and/or Subgroup (eg, Steering Committee)
Kelsey	Linstrum			REMAP-CAP			
Amanda	McNamara			REMAP-CAP			
Alexandra	Weissman			REMAP-CAP			
Dylan	Barbee			REMAP-CAP			
Emily	Berryman			REMAP-CAP			
Hooman	Kamel			STROKENET			
Pooja	Khatri			STROKENET			
Jamey	Frasure			STROKENET			
Amy	Sulken			STROKENET			
Kalli	Beasley			STROKENET			
Narlina	Lalani			VA			
Ashlea	Mayberry			VA			
Windsor	Sheryl			MAHI			
Roche	Sindy			MAHI			
Renato	Lopes			BCRI (Brazil)			
Jéssica Natuline	Ianof			BCRI (Brazil)			
Lilian	Mazza			BCRI (Brazil)			
Julia	Morata			BCRI (Brazil)			
Carolina Martins	Cafarella			BCRI (Brazil)			
Mayra Akimi	Suiama			BCRI (Brazil)			
Daniele	de Lima Franco			BCRI (Brazil)			
Jorge	Escobedo			AVANTI (Mexico)			
Andrea	Martinez			AVANTI (Mexico)			
Pedro	Ohara			AVANTI (Mexico)			
Douglas	Assis			AVANTI (Mexico)			
Aldo	Maggioni			CRF (Italy)			
Chiara	Manzalini			CRF (Italy)			
Stefania	Corsi			CRF (Italy)			
Gianluca	Campo			CRF (Italy)			
Jose	Lopez-Sendon			FIBHULP (Spain)			
Paula	Prieto			FIBHULP			

Supplemental Online Content: Nonauthor Collaborators
*First name, last name, and suffix (if applicable) are required and will appear in PubMed.

***First Name and Middle Initial(s)**	***Last Name**	***Suffix (eg, Jr, III)**	Academic Degrees	Institution	Location (city, state/province, country)	Role or Contribution, eg, chair, principal investigator	Group (if more than 1 Group listed in the byline) and/or Subgroup (eg, Steering Committee)
Rocio	Prieto			FIBHULP			
Rebecca	Wakeman		BA, CCRC, M	University of Florida Health	Gainesville, FL 32610		
Christopher J.	Duncan		BS	University of Florida Health	Gainesville, FL 32610		
Srikar	Savaram		BS	University of Florida Health	Gainesville, FL 32610		
Alexandra Marie	Taylor			University of Florida Health	Gainesville, FL 32610		
Valerie J.	Renard		MSN, AGAN	Duke University Health System	Durham NC		
Grace R.	Dreyer		PA-C	Duke Regional Hospital	Durham NC		
Tatyana	Der		MD, MHSc	Duke Raleigh Hospital	Raleigh, NC		
Emily R.	Ko		MD, PhD	Duke Regional Hospital	Durham NC		
Neil	Stafford		MD	Duke Regional Hospital	Durham NC		
Andrea	Archibald		MD, FACP	Duke Regional Hospital	Durham NC		
Oluwayemisi	Mohammed		MBChB, MS	Duke University Medical Center	Durham NC		
Nkiruka	Azuogalanya		CRC	Duke University Medical Center	Durham NC		
Thomas L.	Ortel		MD, PhD	Duke University Medical Center	Durham NC		
Ari	Moskowitz		MD	Montefiore Medical Center			
Amira	Mohammed			Montefiore Medical Center			
R. Duncan	Hite		MD	University of Cincinnati & University of Cincinnati Medical Center	Cincinnati, OH, USA		
Harshada	More		MS	University of Cincinnati	Cincinnati, OH, USA		
Hammad	Tanzeem		MD	University of Cincinnati	Cincinnati, OH, USA		
Neil	Ernst		Pharm D	University of Cincinnati Medical Center	Cincinnati, OH, USA		
Dalton	Kuebel		Pharm D	University of Cincinnati Medical Center	Cincinnati, OH, USA		
Julia	Vargas		B.S.	UCLA	Los Angeles, CA, USA		
Hena	Sihota		B.S.	UCLA	Los Angeles, CA, USA		
Micah R.	Whitson		MD	University of Alabama Birmingham	Birmingham, Alabama, USA		
Donna S.	Harris		BSN	University of Alabama Birmingham	Birmingham, Alabama, USA		

Supplemental Online Content: Nonauthor Collaborators
*First name, last name, and suffix (if applicable) are required and will appear in PubMed.

***First Name and Middle Initial(s)**	***Last Name**	***Suffix (eg, Jr, III)**	Academic Degrees	Institution	Location (city, state/province, country)	Role or Contribution, eg, chair, principal investigator	Group (if more than 1 Group listed in the byline) and/or Subgroup (eg, Steering Committee)
Jeffrey R	Jacobson		MD	University of Illinois at Chicago	Chicago, IL, USA		
Neha	Atal		MBA, MIIS	University of Illinois at Chicago	Chicago, IL, USA		
María I.	Murillo Blasco		RN	Hospital Zendal	Madrid, Spain		
Francisco	Galiano Cuevas		RN	Hospital Zendal	Madrid, Spain		
Michael	Plump		DO	Rutgers New Jersey Medical School	Newark, NJ, USA		
Bruce	Cusson		RN	Atrium Health Wake Forest Baptist Medical Cente	Winston Salem, NC United States		
Amelia	Eaton		RN	Atrium Health Wake Forest Baptist Medical Cente	Winston Salem, NC United States		
Lynnette	Harris		RN	Atrium Health Wake Forest Baptist Medical Cente	Winston Salem, NC United States		
Mhorys	Pickmans		MD	Atrium Health Wake Forest Baptist Medical Cente	Winston Salem, NC United States		
Jessica	Reeves		RN	Atrium Health Wake Forest Baptist Medical Cente	Winston Salem, NC United States		
Brandon	Reeves		RN	Atrium Health Wake Forest Baptist Medical Cente	Winston Salem, NC United States		
Kinchit	Shah		MD	Atrium Health Wake Forest Baptist Medical Cente	Winston Salem, NC United States		
Tanmay	Sura			Atrium Health Wake Forest Baptist Medical Cente	Winston Salem, NC United States		
Hinna	Wadhwani		MD	Atrium Health Wake Forest Baptist Medical Cente	Winston Salem, NC United States		
Ryan	Maves		MD	Atrium Health Wake Forest Baptist Medical Cente	Winston Salem, NC United States		
Leonard	Stallings		MD	Atrium Health Wake Forest Baptist Medical Cente	Winston Salem, NC United States		
Lilia	Nigro Maia		MD	Fundação Faculdade Regional De Medicina De São José Do Rio Preto	São José do Rio Preto, SP		

*First name, last name, and suffix (if applicable) are required and will appear in PubMed.

***First Name and Middle Initial(s)**	***Last Name**	***Suffix (eg, Jr, III)**	Academic Degrees	Institution	Location (city, state/province, country)	Role or Contribution, eg, chair, principal investigator	Group (if more than 1 Group listed in the byline) and/or Subgroup (eg, Steering Committee)
Nadielly	Prado			Fundação Faculdade Regional De Medicina De São José Do Rio Preto	São José do Rio Preto, SP		
Osvaldo	Silva Junior			Fundação Faculdade Regional De Medicina De São José Do Rio Preto	São José do Rio Preto, SP		
Cláudio	Jorge			Fundação Faculdade Regional De Medicina De São José Do Rio Preto	São José do Rio Preto, SP		
Osana	Costa			Fundação Faculdade Regional De Medicina De São José Do Rio Preto	São José do Rio Preto, SP		
Mariana Buka	Buka			Fundação Faculdade Regional De Medicina De São José Do Rio Preto	São José do Rio Preto, SP		
Danielli	Frassatto			Fundação Faculdade Regional De Medicina De São José Do Rio Preto	São José do Rio Preto, SP		
Paulo	Dutra			Fundação Faculdade Regional De Medicina De São José Do Rio Preto	São José do Rio Preto, SP		
Larissa	da Silva			Fundação Faculdade Regional De Medicina De São José Do Rio Preto	São José do Rio Preto, SP		
Nicolly	Alessio			Fundação Faculdade Regional De Medicina De São José Do Rio Preto	São José do Rio Preto, SP		
Thaise	Pontana			Fundação Faculdade Regional De Medicina De São José Do Rio Preto	São José do Rio Preto, SP		

Supplemental Online Content: Nonauthor Collaborators
*First name, last name, and suffix (if applicable) are required and will appear in PubMed.

***First Name and Middle Initial(s)**	***Last Name**	***Suffix (eg, Jr, III)**	Academic Degrees	Institution	Location (city, state/province, country)	Role or Contribution, eg, chair, principal investigator	Group (if more than 1 Group listed in the byline) and/or Subgroup (eg, Steering Committee)
Natalia	Cordeiro			Fundação Faculdade Regional De Medicina De São José Do Rio Preto	São José do Rio Preto, SP		
Thiago	Souza			Fundação Faculdade Regional De Medicina De São José Do Rio Preto	São José do Rio Preto, SP		
Jaqueline	Mastro			Fundação Faculdade Regional De Medicina De São José Do Rio Preto	São José do Rio Preto, SP		
Jaqueline	Souza			Fundação Faculdade Regional De Medicina De São José Do Rio Preto	São José do Rio Preto, SP		
Cassia	Pradela			Fundação Faculdade Regional De Medicina De São José Do Rio Preto	São José do Rio Preto, SP		
Larissa	Moreira			Fundação Faculdade Regional De Medicina De São José Do Rio Preto	São José do Rio Preto, SP		
Guizela Pavon	Pavon			Fundação Faculdade Regional De Medicina De São José Do Rio Preto	São José do Rio Preto, SP		
Andreza	Rodrigues			Fundação Faculdade Regional De Medicina De São José Do Rio Preto	São José do Rio Preto, SP		
Anna	Centurione			Fundação Faculdade Regional De Medicina De São José Do Rio Preto	São José do Rio Preto, SP		
Joelma	Silva			Fundação Faculdade Regional De Medicina De São José Do Rio Preto	São José do Rio Preto, SP		

Supplemental Online Content: Nonauthor Collaborators
*First name, last name, and suffix (if applicable) are required and will appear in PubMed.

***First Name and Middle Initial(s)**	***Last Name**	***Suffix (eg, Jr, III)**	Academic Degrees	Institution	Location (city, state/province, country)	Role or Contribution, eg, chair, principal investigator	Group (if more than 1 Group listed in the byline) and/or Subgroup (eg, Steering Committee)
Katia	Andreoti			Fundação Faculdade Regional De Medicina De São José Do Rio Preto	São José do Rio Preto, SP		
Renan	Vectorazzo			Fundação Faculdade Regional De Medicina De São José Do Rio Preto	São José do Rio Preto, SP		
Tamires Silva	Silva			Fundação Faculdade Regional De Medicina De São José Do Rio Preto	São José do Rio Preto, SP		
Juliana	Garcia			Fundação Faculdade Regional De Medicina De São José Do Rio Preto	São José do Rio Preto, SP		
Ana Paula	Demore			Fundação Faculdade Regional De Medicina De São José Do Rio Preto	São José do Rio Preto, SP		
Alberto	Papi		MD	University of Ferrara	Ferrara FE, Italy		
Luca	Ronzoni		MD	University of Ferrara	Ferrara FE, Italy		
Jose Luis	Lopez-Sendon Moreno		M.D.	Hospital Ramón y Cajal (IRYCIS)	Madrid. SPAIN.	Principal Investigator	
Itziar Pozuelo	Echegaray		PhD	Hospital Ramón y Cajal (IRYCIS)	Madrid. SPAIN.	Sub-Investigator	
Chowdhury	Ahsan		MD	University Medical Center of Southern Nevada	Las Vegas, NV	Principal Investigator	
Aaron E.	Kornblith		MD	University of California, San Francisco	San Francisco, CA, USA	co-investigator	
Carolyn M.	Hendrickson		MD	University of California, San Francisco	San Francisco, CA, USA	co-investigator	
Fernando	Worner		PhD	Hospital Universitari Arnau de Vilanova. IRBLLEIDA	Lleida. SPAIN	Principal Investigator	
P	Pastor		MD	Hospital Universitari Arnau de Vilanova. IRBLLEIDA	Lleida. SPAIN	Investigator	

Supplemental Online Content: Nonauthor Collaborators
*First name, last name, and suffix (if applicable) are required and will appear in PubMed.

***First Name and Middle Initial(s)**	***Last Name**	***Suffix (eg, Jr, III)**	Academic Degrees	Institution	Location (city, state/province, country)	Role or Contribution, eg, chair, principal investigator	Group (if more than 1 Group listed in the byline) and/or Subgroup (eg, Steering Committee)
JL	Morales		MD	Hospital Universitari Arnau de Vilanova. IRBLLEIDA	Lleida. SPAIN	Investigator	
Jesus	Peteiro		MD	Complejo Universitario Universitario de A Coruña (CHUAC)		Principal Investigator	
Rita Galeiras	Vázquez			Complejo Universitario Universitario de A Coruña (CHUAC)	A Coruña, Spain	Subinvestigator	
José María	Sanchez-Andrade Bolaños			Complejo Universitario Universitario de A Coruña (CHUAC)	A Coruña, Spain	Subinvestigator	
Muhib A	Khan		MD	Spectrum Health (Butterworth Hospital)	Grand Rapids, Michigan, U	Site Principal-Investigator	
Malik MHS	Khan		MD	Spectrum Health (Butterworth Hospital)	Grand Rapids, Michigan, U	Site Sub-Investigator	
Heather C	Brooks		BSN, CCRC	Spectrum Health (Butterworth Hospital)	Grand Rapids, Michigan, U	Site Clinical Research Nurse	
Matt	Prekker			Hennepin County Medical Center	Minneapolis, Minnesota, USA	PI	
Zahra A.	Ajani		MD	Kaiser Permanente Los Angeles	Los Angeles, CA, USA	Site PI/ Clinical Investigator	
Jorge	Moisés		MD, PhD	Hospital Clínic de Barcelona	Barcelona - Spain	PI	
Jeisson	Osorio		MD	Hospital Clínic de Barcelona-IDIBAPS	Barcelona - Spain	study coordinator	
Jesús	Aibar		MD, PhD	Hospital Clínic de Barcelona	Barcelona - Spain	sub-investigator	
Vidya	Krishnan		MD MHS	Case Western Reserve University - MetroHealth campus	Cleveland, OH, USA	PI	
Matthew C	Exline		MD MPH	Ohio State University Wexner Medical Center	Columbus, OH, USA	Site Principal Investigator	
Elizabeth L	Schwartz		BSPH	Ohio State University Wexner Medical Center	Columbus, OH, USA	Site Coordinator	

Supplemental Online Content: Nonauthor Collaborators
*First name, last name, and suffix (if applicable) are required and will appear in PubMed.

***First Name and Middle Initial(s)**	***Last Name**	***Suffix (eg, Jr, III)**	Academic Degrees	Institution	Location (city, state/province, country)	Role or Contribution, eg, chair, principal investigator	Group (if more than 1 Group listed in the byline) and/or Subgroup (eg, Steering Committee)
Matthew	Kutcher		MD MS FA(	University of Mississippi Medical Center	Jackson, MS	Site Principal Investigator	
Emily	Grenn			University of Mississippi Medical Center	Jackson, MS	Study Coordinator	
Taylor	Shaw			University of Mississippi Medical Center	Jackson, MS	Study Coordinator	
Simon	Barinas			University of Mississippi Medical Center	Jackson, MS	Lead Study Coordinator	
Stephanie C.	Guo		MD	Queen's Medical Center	Honolulu, HI. USA	Site Principal investigator	
Todd B.	Seto		MD	Queen's Medical Center	Honolulu, HI. USA	Co-investigator	
Lewis	Satterwhite		MD	Kansas University Medical Center	Kansas City, KS, USA	Site Principal investigator	
Julius Gene	Latorre		M.D.,M.P.H	SUNY Upstate University Hospital	Syracuse, NY, USA	Site Principal Investigator	
Lena F.	Deb		B.A.	SUNY Upstate University Hospital	Syracuse, NY, USA	Research Coordinator	
Conrad W	Liang		MD, PhD	Kaiser Permanente Fontana Medical Center	Fontana, CA, USA	Site PI	
Vanessa F	Audea			Kaiser Permanente Fontana Medical Center	Fontana, CA, USA	Research Coordinator	
Nitin K	Puri		MD	Cooper Medical School of Rowan University Cooper University Hospital	Camden, New Jersey, USA	Site -Primary Investigator	
Adam	Green		MD, MBA	Cooper Medical School of Rowan University Cooper University Hospital	Camden, New Jersey, USA	Sub investigator	
Abhijit	Duggal		MD	Cleveland Clinic Foundation Main	Cleveland, OH, USA	Site PI	
Girish B.	Nair		MD, MS	William Beaumont University Hospital	Royal Oak, Michigan	Site Principal Investigator	

*First name, last name, and suffix (if applicable) are required and will appear in PubMed.

***First Name and Middle Initial(s)**	***Last Name**	***Suffix (eg, Jr, III)**	Academic Degrees	Institution	Location (city, state/province, country)	Role or Contribution, eg, chair, principal investigator	Group (if more than 1 Group listed in the byline) and/or Subgroup (eg, Steering Committee)
Sanjay	Dogra		MD	William Beaumont University Hospital	Royal Oak, Michigan	Co-I	
Jordan B	Schooler		MD, PhD	Penn State Milton S Hershey Medical Center	Hershey, PA, USA	Site Principal Investigator	
Steven C	Moore		MD	Penn State Milton S Hershey Medical Center	Hershey, PA, USA	Site Principal Investigator	
Scott	Kaatz			Henry Ford Medical Center	Detroit, MI, USA	Principal Investigator	
Manila	Gaddh			Emory University School of Medicine	Atlanta, GA, USA.	Site Principal Investigator	
Bassel	Atassi		M.D.	OSF Little Company of Mary Medical Center (OSF LCM)	Evergreen Park, Illinois	study PI	
Rajat	Kapoor		MD, MBA	Indiana University School of Medicine	Indianapolis, Indiana, USA	Site Principal Investigator	
Alvaro	Alonso A		MD, MSCR,	University of Massachusetts Chan Medical School and UMassMemorial Medical Center	Worcester, MA, USA	PI	
Alexander P.	Hamel		BS	University of Massachusetts Chan Medical School and UMassMemorial Medical Center	Worcester, MA, USA	Research Coordinator	
Mauricio	Leitao		MD, MBA	University of Massachusetts Chan Medical School and UMassMemorial Medical Center	Worcester, MA, USA	Research Coordinator	
Angela J.	Rogers		MD. MPH	Stanford University School of Medicine	Palo Alto, California	Site Principal Investigator	
Sergio	Babudieri			AOU Sassari, Sassari	Sassari, Italy	Site Principal Investigator	
Johanna I	Busch		MD, PhD, M	Dell Medical School at the University of Texas at Austin	Austin, TX	Site Principal Investigator	

*First name, last name, and suffix (if applicable) are required and will appear in PubMed.

*First Name and Middle Initial(s)	*Last Name	*Suffix (eg, Jr, III)	Academic Degrees	Institution	Location (city, state/province, country)	Role or Contribution, eg, chair, principal investigator	Group (if more than 1 Group listed in the byline) and/or Subgroup (eg, Steering Committee)
Hemali	Patel		MD	Dell Medical School at the University of Texas at Austin	Austin, TX	Site Principal Investigator	
John	Kostis			Rutgers Robert Wood Johnson Medical School	New Brunswick, NJ	Site Principal Investigator	
Binita	Shah		MD, MS	VA NY Harbor Health System and NYU School of Medicine	New York, NY, USA	Site Principal Investigator	
Michael	Matthay		MD	University of California, San Francisco	San Francisco, CA, USA	Site Principal Investigator	
Rachel M.	Gropper		BS	University of California, San Francisco	San Francisco, CA, USA	Clinical Research Coordinator	
Anika	Agrawal		BA	University of California, San Francisco	San Francisco, CA, USA	Clinical Research Coordinator	
Kimia	Ashktorab		BS	University of California, San Francisco	San Francisco, CA, USA	Clinical Research Coordinator	
Bellal	Joseph		MD	University of Arizona	Tucson, AZ	Site Principal Investigator	
Janine R E.	Vintch		MD	The Lundquist Institute for Biomedical Innovation at Harbor-UCLA Medical Center	Torrance, California, USA	Site Principal Investigator	
David	Yuchno		MD	The Lundquist Institute for Biomedical Innovation at Harbor-UCLA Medical Center	Torrance, California, USA	co-PI	
Cristiano	Perdeneiras Jaeger			Hospital Universitário de Canoas	São José, Brazil	Site Principal Investigator	
Antonella	dArminio Monforte			ASST Santi Paolo e Carlo, Milano	Milano, Italy	Site Principal Investigator	
Luis R	Garcia-Cortes		Master in C	Instituto Mexicano del Seguro Social	Naucalpan, State of Mexico East, Mexico		
Blanca E	Herrera-Morales		Master of H	Instituto Mexicano Del Seguro Social, Regional General Hospital 196	Ecatepec State of Mexico East, Mexico		
Eleonora	Gashi		DO, MPhil,	Jacobi Medical Center	Bronx, New York	Site Principal Investigator	

Supplemental Online Content: Nonauthor Collaborators
*First name, last name, and suffix (if applicable) are required and will appear in PubMed.

***First Name and Middle Initial(s)**	***Last Name**	***Suffix (eg, Jr, III)**	Academic Degrees	Institution	Location (city, state/province, country)	Role or Contribution, eg, chair, principal investigator	Group (if more than 1 Group listed in the byline) and/or Subgroup (eg, Steering Committee)
Seth	Sokol		MD	Jacobi Medical Center	Bronx, New York	Site Co-PI	
Paul	Simonelli			Geisinger Research	Danville, PA	Site Principal Investigator	
Robert	Sherwin			Detroit Receiving Hospital	Detroit, MI, USA	Site Principal Investigator	
Adam	Ackerman		MD	University of Vermont Medical Center	Burlington, VT USA	Site Principal Investigator	
Christos	Colovos		MD PhD	University of Vermont Medical Center	Burlington, VT USA	Former Principle Investigator	
Alexandre	de Matos Soeiro			Instituto do Coração do Hospital das Clínicas da Faculdade de Medicina da USP-InCor-HCFMUSP	São Paulo, Brazil	Site Principal Investigator	
Murillo	de Oliveira Antunes		MO, MD, P	Hospital Universitário São Francisco Na Providencia de Deus	Bragança Paulista-sp,Brazil	Site Principal Investigator	
Costa	Tibério Augusto Oliveira		MD	Hospital Universitário São Francisco Na Providencia de Deus	Bragança Paulista-sp,Brazil	Sub investigator	
Melvin R.	Echols		MD	Morehouse School of Medicine	Atlanta, GA	Site Principal Investigator	
John P.	Sheehan		MD	University of Wisconsin School of Medicine and Public Health	Madison, WI, USA	Site Principal Investigator	
Kraig T.	Kumfer		MD-PhD	University of Wisconsin School of Medicine and Public Health	Madison, WI, USA	Co-Investigator	
Paulo	Caramori		MD	The Catholic University of Rio Grande do Sul	Rio Grande do Sul, Brazil		
Otávio Fachinetto	Casagrande		MD, PhD	The Catholic University of Rio Grande do Sul	Rio Grande do Sul, Brazil		
Carlos	Carpio		MD	Hospital Universitario La Paz		Site Principal Investigator	
Ester	Zamarrón		MD	Hospital Universitario La Paz		SI	
Rodolfo	Álvarez-Sala		MD	Hospital Universitario La Paz	Madrid, Spain	SI	
Nicola	Coppola		PhD	Policlinico di Napoli, Napoli	Napoli, Italy	Site Principal Investigator	
Antonio	Russo		PhD	University of Campania	Naples, Italy	Co-Investigator	

Supplemental Online Content: Nonauthor Collaborators
*First name, last name, and suffix (if applicable) are required and will appear in PubMed.

*First Name and Middle Initial(s)	*Last Name	*Suffix (eg, Jr, III)	Academic Degrees	Institution	Location (city, state/province, country)	Role or Contribution, eg, chair, principal investigator	Group (if more than 1 Group listed in the byline) and/or Subgroup (eg, Steering Committee)
Jeffrey	Berger			NYU Langone Brooklyn	Brooklyn, NY	Site Principal Investigator	
Mark A.	Tidswell		MD	UMASS Chan Medical School-Baystate	Springfield MA USA	Site Principal Investigator	
Jay S.	Steingrub		MD	UMASS Chan Medical School-Baystate	Springfield MA USA	Site Co-Investigator	
Shane	O'Mahony			Swedish Hospital	Seattle, Washington	Site Principal Investigator	
David T.	Huang		MD, MPH	University of Pittsburgh		Site Principal Investigator	
Nitin K	Puri		MD	Cooper Health			
Idelzuita	Leandro Liporace			Instituto Dante Pazzanese de Cardiologia	São Paulo, Brazil	Site Principal Investigator	
Stephen	Pan		MD MS	Westchester Medical Center / NY Medical College	Valhalla, NY USA		
Ravi J.	Shah		MD	Westchester Medical Center / NY Medical College	Valhalla, NY USA		
Michelle	Gong			Montefiore Medical Center - Weiler	Bronx, NY	Site Principal Investigator	
Akram	Khan		MD (MBBS	Oregon Health & Science University	Portland, Oregon		
Minn	Oh		Ph.D	Oregon Health & Science University	Portland, Oregon		
Matthew	Lammi			University Medical Center-LSU	New Orleans, LA	Site Principal Investigator	
Shane E	Sanne		DO	LSU Health New Orleans	New Orleans, LA	Co-Investigator	
Todd	Costantini		MD	UC San Diego School of Medicine	San Diego, CA, USA	Site Principal Investigator	
Allison E.	Berndtson		MD	UC San Diego School of Medicine	San Diego, CA, USA	Site co-investigator	
Eduardo	Mateos			Hospital de Infectologia Centro Medico Nacional La Raza	Mexico City< Mexico	Site Principal Investigator	
Srikanth	Vallurupalli		MD	University of Arkansas for Medical Sciences		Site Principal Investigator	

Supplemental Online Content: Nonauthor Collaborators
*First name, last name, and suffix (if applicable) are required and will appear in PubMed.

***First Name and Middle Initial(s)**	***Last Name**	***Suffix (eg, Jr, III)**	Academic Degrees	Institution	Location (city, state/province, country)	Role or Contribution, eg, chair, principal investigator	Group (if more than 1 Group listed in the byline) and/or Subgroup (eg, Steering Committee)
Srilakshmi	Ravula		MD	University of Arkansas for Medical Sciences		Site co-investigator	
Abhijit	Duggal			Cleveland Clinic Foundation Weston	Weston, FL	Site Principal Investigator	
Michael	Bromberg			Temple University			
Mark B	Effron		MD	University of Queensland – Ochsner Clinical School	New Orleans, LA, USA	Site Principal Investigator	
Allyson M.	Pishko		MD MSCE	Perelman School of Medicine; University of Pennsylvania	Philadelphia, PA	Site Principal Investigator	
Alice	Cohen		M.D., F.A.C	Newark Beth Israel Medical Center & Children's Hospital of New Jersey	Newark, NJ	Site Principal Investigator	
Cynthia	Horta		RN	Newark Beth Israel Medical Center & Children's Hospital of New Jersey	Newark, NJ	Lead Study Coordinator	
James	Jaffe			Doctors Medical Center of Modesto, Inc.	Modesto, CA	Site Principal Investigator	
