# Supplementary material for: Effect of P2Y12 Inhibitors on Organ Support–Free Survival in Critically Ill Patients Hospitalized for COVID-19: A Randomized Clinical Trial
Source: JAMA Netw Open. 2023 May 25;6(5):e2314428. doi: 10.1001/jamanetworkopen.2023.14428 (PMC10214036; doi:10.1001/jamanetworkopen.2023.14428)
Supplement: Supplement 4. — Data Sharing Statement [file jamanetwopen-e2314428-s004.pdf]

Data Sharing Statement

Berger. Effect of P2Y12 Inhibitors on Organ Support–Free Survival in Critically Ill Patients Hospitalized for COVID-19. *JAMA Netw Open*. Published May 25, 2023.
doi:10.1001/jamanetworkopen.2023.14428

Data
Data available: Yes
Data types: Deidentified participant data
How to access data: Study data will be shared through the NHBLI data repository, BioData Catalyst, in six months. www.ncbi.nlm.nih.gov
When available: beginning date: 05-10-2023

Supporting Documents
Document types: Statistical/analytic code
How to access documents: Supplemental Materials
When available: With publication

Additional Information
Who can access the data: Researchers whose proposed use of the data has been approved.
Types of analyses: For a specified purpose.
Mechanisms of data availability: After approval of a proposal, or with a signed data access agreement.
